# Estimating taxonomic and functional structure along a tropical estuary: linking metabolic traits and aspects of ecosystem functioning

**DOI:** 10.1128/spectrum.03886-23

**Published:** 2024-08-20

**Authors:** Héctor A. Levipan, L. Felipe Opazo, Sara Arenas-Uribe, Hernán Wicki, Francisca Marchant, Lennin Florez-Leiva, Ruben Avendaño-Herrera

**Affiliations:** 1 Departamento de Ciencias y Geografía, Facultad de Ciencias Naturales y Exactas, Laboratorio de Ecopatología y Nanobiomateriales, Universidad de Playa Ancha, Valparaíso, Chile; 2 Ocean, Climate and Environment Research Group (OCE), Environmental Academic Corporation, University of Antioquia, Medellín, Colombia; 3 Departamento de Ecología, Facultad de Ciencias, Universidad Católica de la Santísima Concepción, Concepción, Chile; 4 Institute of Ecology and Biodiversity (IEB), Santiago, Chile; 5 Departamento de Ecología, Facultad de Ciencias Biológicas, Pontificia Universidad Católica de Chile, Santiago, Chile; 6 Programa de Magíster en Ecología Marina, Universidad Católica de la Santísima Concepción, Concepción, Chile; 7 Facultad de Ciencias de la Vida, Laboratorio de Patología de Organismos Acuáticos y Biotecnología Acuícola, Universidad Andrés Bello, Viña del Mar, Chile; 8 Centro FONDAP, Interdisciplinary Center for Aquaculture Research (INCAR), Universidad Andrés Bello, Viña del Mar, Chile; 9 Centro de Investigación Marina Quintay (CIMARQ), Universidad Andrés Bello, Quintay, Chile; Dominican University New York, Orangeburg, New York, USA

**Keywords:** Colombia’s Caribbean Sea, Atrato River, Gulf of Urabá, 16S amplicon sequencing, functional diversity, functional space, multiple-trait approach

## Abstract

Microbial life forms are among the most ubiquitous on Earth, yet many remain understudied in Caribbean estuaries. We report on the prokaryote community composition of the Urabá Estuary in the Colombian Caribbean using 16S rRNA gene-transcript sequencing. We also assessed potential functional diversity through 38 metabolic traits inferred from 16S rRNA gene data. Water samples were collected from six sampling stations at two depths with contrasting light-penetration conditions along an approximately 100 km transect in the Gulf of Urabá in December 2019. Non-metric multidimensional scaling analysis grouped the samples into two distinct clusters along the transect and between depths. The primary variables influencing the prokaryote community composition were the sampling station, depth, salinity, and dissolved oxygen levels. Twenty percent of genera (i.e., 58 out 285) account for 95% of the differences between groups along the transect and among depths. All of the 38 metabolic traits studied showed some significant relationship with the tested environmental variables, especially salinity and except with temperature. Another non-metric multidimensional scaling analysis, based on community-weighted mean of traits, also grouped the samples in two clusters along the transect and over depth. Biodiversity facets, such as richness, evenness, and redundancy, indicated that environmental variations—stemming from river discharges—introduce an imbalance in functional diversity between surface prokaryote communities closer to the estuary’s head and bottom communities closer to the ocean. Our research broadens the use of 16S rRNA gene transcripts beyond mere taxonomic assignments, furthering the field of trait-based prokaryote community ecology in transitional aquatic ecosystems.

**IMPORTANCE:**

The resilience of a dynamic ecosystem is directly tied to the ability of its microbes to navigate environmental gradients. This study delves into the changes in prokaryote community composition and functional diversity within the Urabá Estuary (Colombian Caribbean) for the first time. We integrate data from 16S rRNA gene transcripts (taxonomic and functional) with environmental variability to gain an understanding of this under-researched ecosystem using a multi-faceted macroecological framework. We found that significant shifts in prokaryote composition and in primary changes in functional diversity were influenced by physical-chemical fluctuations across the estuary’s environmental gradient. Furthermore, we identified a potential disparity in functional diversity. Near-surface communities closer to the estuary’s head exhibited differences compared to deeper communities situated farther away. Our research serves as a roadmap for posing new inquiries about the potential functional diversity of prokaryote communities in highly dynamic ecosystems, pushing forward the domain of multi-trait-based prokaryote community ecology.

## INTRODUCTION

Estuarine ecosystems account for less than 1% of the global ocean area (1), but they are recipients of substantial amounts of organic and inorganic materials originating from human wastes, agriculture, and other anthropogenic sources (2). Pollutants of high concern to human health, such as atrazine (3), dichloro-diphenyl-trichloroethane compounds (4), and other emerging toxic xenobiotis (e.g., nonylphenol ethoxylates, organophosphate flame retardants) (5, 6), are commonly detected in estuarine water and sediment samples. In addition, most of the organic load reaching tropical continental margins—including their estuaries—is released into the atmosphere as carbon dioxide, facilitated by biological respiration and microbial-loop-mediated decomposition (7). In turn, estuarine uptake of carbon dioxide from the atmosphere is not restricted to photosynthesis and chemolithoautotrophy (8, 9), as it may be importantly mediated by heterotrophic inorganic carbon fixation (10). This process is thought to occur in estuaries via anaplerotic reactions (11), which utilize enzymatic components of the pyruvate metabolism to generate intermediaries in major biological reactions (7).

Prokaryote communities, encompassing both Bacteria and Archaea, serve as pivotal components of the microbial loop in estuaries (12). These communities demonstrate a highly dynamic composition and functionality (13, 14), influenced by environmental gradients such as pH, salinity, temperature, organic matter, and nutrient influxes. These gradients arise from combining chemically distinct water sources (15, 16). For instance, changes in the composition and metabolic profiles of prokaryotic communities across river-to-sea transects have been associated with changes in metabolic fluxes in sediment samples from one of the largest estuarine bays in Australia (17). In the same study, the presence of mannitol was associated with fructose and mannose metabolism, while the detection of succinic acid was considered indicative of propanoate metabolism, butanoate metabolism, the citrate cycle, and glyoxylate and dicarboxylate metabolism. In addition, the detection of valine and α-ketoisovaleric acid was attributed to the metabolism of complex amino acids such as valine, leucine, and isoleucine possibly via propanoate metabolism, butanoate metabolism, pyruvate metabolism, the citrate cycle, or -alanine pathways. The performance of these metabolic pathways along the river-to-sea transitions likely depends on specific microbial adaptation strategies, such as the expression of genes encoding potassium, sodium, and glycine betaine transporters for osmoregulation (18).

Understanding the spatial dynamics of prokaryote communities in habitats subject to changing conditions and strong human pressures (e.g., urbanization, mining, and chemical pollution) is crucial for finding indicators of water quality and for determining the ecological condition of habitats for conservation and management purposes (19). Remarkably, the increasing body of knowledge on estuarine ecosystems worldwide (7, 20, 21) contrasts with the lack of studies on free-living prokaryote communities in tropical estuaries of the South American continental margin. This is the case for the Urabá Estuary in the Caribbean Sea of Colombia, an ecosystem with increasing human-driven disturbances (22, 23) and unknown prokaryote communities in terms of taxonomic composition and functioning. Only one study has investigated bacteriological aspects linked to environmental health and sanitary quality in this area (24). Currently, more information is available on the Gulf of Urabá for primary producers that have been affiliated with 7 divisions, 9 classes, 18 orders, 21 families, and 22 genera such as *Anabaena*, *Pseudoanabaena*, and *Oscillatoria* (25) in addition to genera that covary with environmental gradients (26).

The rapid advancement of molecular and bioinformatic tools has facilitated the creation of increasingly expansive prokaryote diversity databases. However, understanding and predicting microbial processes remains a significant challenge, often referred to as the “holy grail” of microbial ecology. For example, discerning the relationships between microbial diversity and ecosystem functioning could lead to substantial progress in this field. This could be achieved by creating new frameworks (27) and/or adopting approaches inspired by classical macroecological theory (28). Functional diversity, a facet of biodiversity, offers insights into the abundance and composition of physiological and/or ecological traits present in biological communities within a specific ecosystem (29). Within this framework, community components are seen as interacting entities. These entities possess functional and metabolic strategies tailored to specific environmental variables. As a result, their environmental impacts can be discerned from functional traits rather than solely from taxonomic diversity (30–32). Predictions based on character-environment relationships can shed light on how environmental factors influence the distribution of an organism’s functional traits along environmental gradients (33).

However, the inherent challenges in distinguishing morphological traits among prokaryote community members and difficulties in discerning their physiological features compared to higher macrobiota pose challenges. Additionally, the low cultivability of prokaryotes (34) further complicates the application of this approach to natural communities. To address this, some researchers have turned to eco-physiological traits (28, 35, 36). The ability of estuarine prokaryotes to adapt to environmental gradients might be linked to metabolic traits inferred from 16S rRNA gene sequences (37, 38). This approach can help estimate functional diversity descriptors (39) and ecosystem functioning across various temporal and spatial scales. For example, 16S rRNA gene sequencing coupled with metabolomics data revealed high functional redundancy associated with key metabolic pathways in bacterial communities from estuarine sediments that could not be recognized based on metabolite detection and identification alone (40). Among the predicted pathways, nucleotide metabolism, terpenoid and polyketide metabolism, lipid metabolism, glycan biosynthesis, energy metabolism, and carbohydrate and amino-acid metabolism were highlighted.

In this study, we focused on the understudied and human-impacted Urabá Estuary. We studied this tropical Caribbean estuary (during the dry season) as a transitional water system between land and sea to investigate how prokaryote community composition and functional diversity are affected along environmental gradients. Our goal was to provide a taxonomic characterization of Caribbean estuarine prokaryote communities using 16S rRNA gene sequencing. In addition, we aimed to expand the use of 16S rRNA gene data to understand estuarine ecosystem functioning through a multiple-trait approach. We hope that the use of multiple functional traits in microbial ecology will be helpful in interpreting prokaryote community responses to gradients of environmental variables in transitional water ecosystems. We anticipated finding prokaryote communities with taxonomic compositions influenced by horizontal and depth gradients. Furthermore, we hypothesized that the contribution of metabolic traits to ecosystem functioning varies in response to environmental gradients along the Urabá Estuary, with prokaryote communities at the head of the estuary characterized by a high degree of functional redundancy due to freshwater inputs.

## RESULTS

### Environmental setting

**Fig 1:**
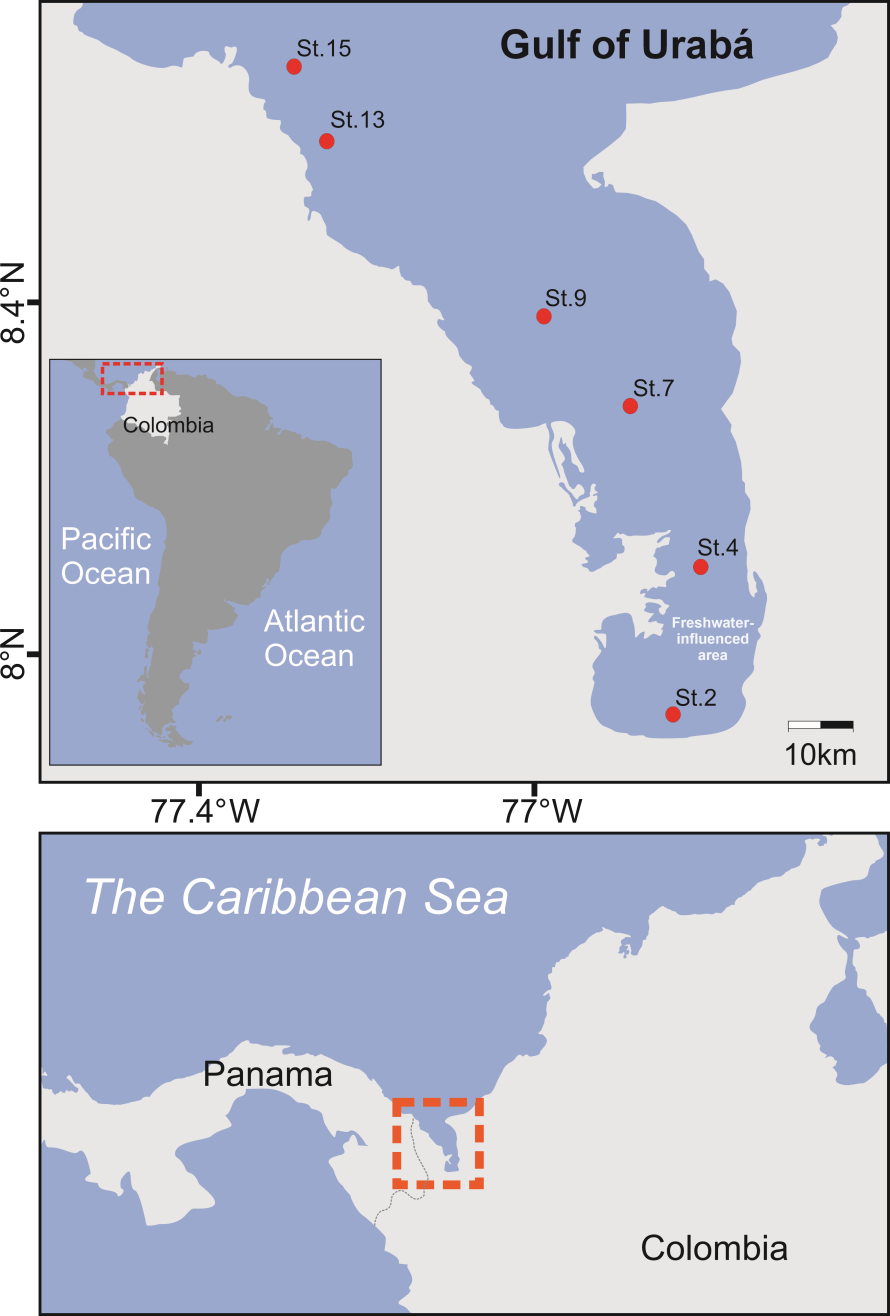
Study area and locations of the sampling sites. Sampling stations (red dots) are distributed along the gulf of the Urabá Estuary. Map of the study zone plotted in Ocean Data View (ODV).

A transect of almost 100 km along the Urabá Estuary was arranged from a freshwater-influenced area (also referred to the estuary’s head) at St2 toward the open ocean (Fig. 1). The brackish plume, caused by the freshwater input into the head of the estuary, was perceptible until approximately St9 (Fig. S1). Temperature and salinity profiles exhibited clines normally detected in the first 5 m of depth (Table 1) (Fig. S1A and B) and were associated with concentrations of dissolved oxygen ranging from approximately 7.1 to 8.8 mg L^−1^ (Table 1). Vertical profiles of environmental variables across the water column showed conditions that favored vertical stratification, as supported by Brunt-Väisälä frequency values that indicated the influence of river discharges over seawater (Fig. S1C).

**TABLE 1:** Numerical identities, coordinates, and characteristics of the sampling stations in the Urabá Estuary

Station	Lat. (N)	Long. (W)	Max. depth (m)	Secchi disk depth (m)	Halocline (m)	Thermocline (m)	Oxygen ($\overset{-}{x}$ ± SD) (mg L^−1^)	
							0.5 m	11 m
2	7.9324	−76.8331	20	<1	10	5	7.77 ± 0.11	8.05 ± 0.07
4	8.0999	−76.8003	19	<1	8–10	5	8.03 ± 0.04	8.05 ± 0.08
7	8.2829	−76.8819	43	1	5.4	2	7.70 ± 0.23	7.79 ± 0.18
9	8.3836	−76.9837	52	1.2	5.4	2	7.68 ± 0.10	7.47 ± 0.22
13	8.5850	−77.2470	44	2.1	5.5	2–5	8.34 ± 0.34	7.89 ± 0.18
15	8.6670	−77.2860	25	2.5	5	2–5	7.59 ± 0.07	7.26 ± 0.19

^*a*^ Halocline and thermocline represent average values between seasons.

### Taxonomic diversity of prokaryote estuarine communities

**Fig 2:**
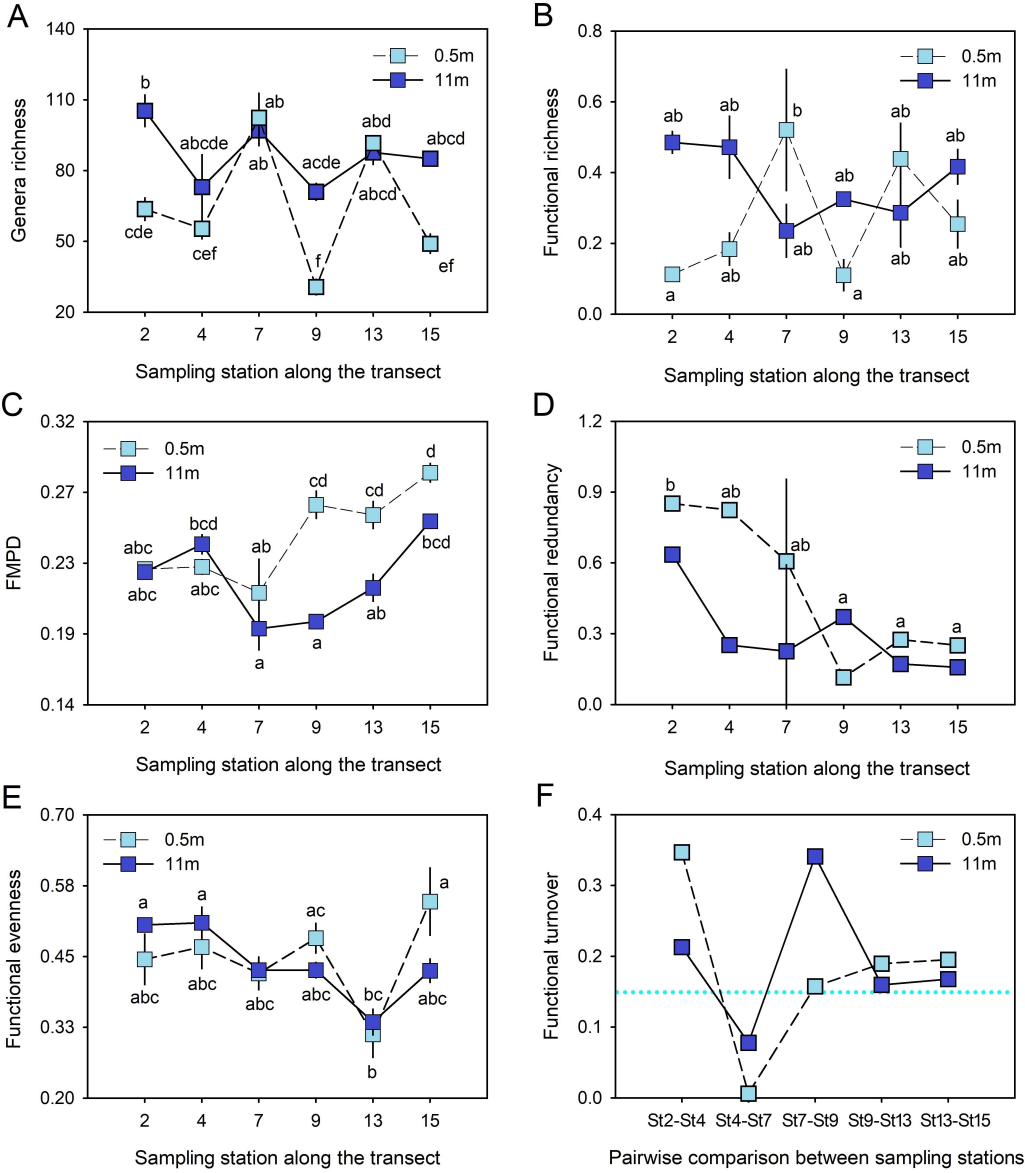
Spatial variation in genera richness and functional facets of prokaryote communities across the Urabá Estuary. (**A**) Genera richness, (**B**) functional richness, (**C**) functional mean pairwise distance (FMPD), (**D**) functional redundancy, (**E**) functional evenness, and (**F**) beta turnover diversity contrasted with the average beta turnover curve estimated from all pools [dotted line (••••)]. Data are averages ± standard deviations of triplicate samples. Significant pairwise differences identified by Tukey’s HDS test are indicated with different letters.

A total of 536 prokaryote genera were distributed across the transect from St2 to St15 (Fig. 1). These genera accounted for a total of 102 orders and were highlighted by an abundance *Alteromonadales*, *Betaproteobacteriales*, Chloroplast (representatives from *Nostocales*, *Pseudanabaenales*, *Limnotrichales*, and *Phormidesmiales*), *Flavobacteriales*, *Oceanospirillales*, *Rhodobacterales*, *Rhodospirillales*, SAR11 and SAR86 clades, and *Synechococcales* (Fig. S2). Fluctuations in average genera richness showed an important coincidence between depths along the transect; these fluctuations were larger at 0.5 m depth (Fig. 2A). Taxa richness tended to decrease slightly along the transect at the depths of 0.5 m (from $\overset{-}{x}$ ~ 63 ± 8.14 to $\overset{-}{x}$ ~ 49 ± 7) and 11 m (from $\overset{-}{x}$ ~ 105 ± 11.5 to $\overset{-}{x}$ ~ 85 ± 4) (Fig. 2A). Aphotic samples (at 11 m depth) held a significantly higher number of genera ($\overset{-}{x}$ ~ 86.5 ± 13.2) compared to surficial samples ($\overset{-}{x}$ ~ 65.5 ± 26.8) (Table 2) and were associated with depth-related differences at St2, St9, and St15 (Table S1).

**TABLE 2:** Two-way ANOVA testing for the effects of the distance along the transect (station) and depth on genera richness and functional descriptors of prokaryote communities^*a*^

Variable	Effect	SS	DF	MS	*F*
Genera richness	(Intercept)	207,784.0	1	207,784.0	930.50***
	Station	10,041.1	5	2,008.2	8.99***
	Depth	3,990.0	1	3,990.0	17.86***
	Station × Depth	3,533.1	5	706.6	5.76***
	Error	6,475.8	29	223.3	
Functional richness	(Intercept)	3.688	1	3.688	201.461***
	Station	0.098	5	0.019	1.076^n.s^.
	Depth	0.089	1	0.089	4.915*
	Station × Depth	0.507	5	0.105	5.548**
	Error	0.439	24	0.018	
Functional mean pairwise distance (FMPD)	(Intercept)	1.958	1	1.958	8,077.918***
	Station	0.016	5	0.003	13.478***
	Depth	0.006	1	0.006	26.957***
	Station × Depth	0.007	5	0.001	6.140***
	Error	0.005	24	0.0002	
Functional redundancy	(Intercept)	5.609	1	5.609	87.570***
	Station	1.388	5	0.277	4.334**
	Depth	0.307	1	0.307	4.804*
	Station × Depth	0.598	5	0.119	1.867^n.s^.
	Error	1.537	24	0.064	
Functional evenness	(Intercept)	7.023	1	7.023	2,324.057***
	Station	0.118	5	0.023	7.842***
	Depth	0.0005	1	0.0005	0.186^n.s^.
	Station × Depth	0.035	5	0.007	2.353^n.s^.
	Error	0.0725	24	0.003	

^*a*^ SS, sum of square; DF, degree of freedom; MS, mean square, *F*, *F*-statistic. The level of statistical significance for each effect is denoted by asterisks (*P < 0.05, **P < 0.01 ***P < 0.001), n.s., not significant effect.

**Fig 3:**
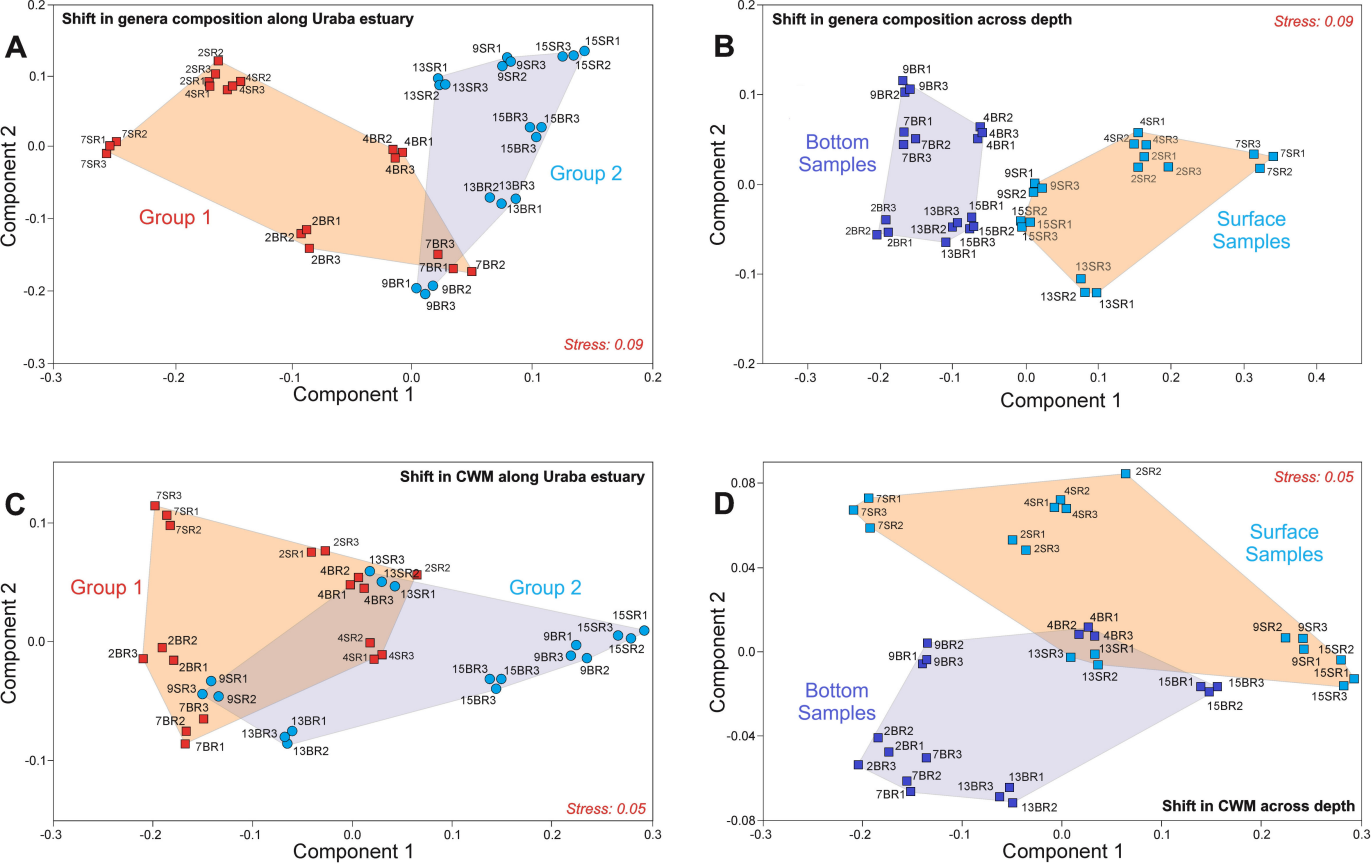
Changes in genera composition and metabolic traits of prokaryote communities in the Urabá Estuary. NMDS ordination plots based on 12 samples in triplicate illustrate the differences in genus composition and community-weighted means (CWMs) (**A, C**) along the Urabá Estuary and (**B, D**) at two depths. Significant distinctive clusters (PERMANOVA, *P*  =  0.001) are identified at genera and trait composition levels across transect and depth (convex hulls). All ordinations were performed using the Euclidean distance.

The Non-metric multidimensional scaling (NMDS) ordination indicated two distinctive groups of samples based on the changes in prokaryote community composition (at genus level) along the transect and among depths (Fig. 3A and B). Samples from St2 to St7 formed the first cluster along the transect, while samples from St9 to St15 made up the second one (Fig. 3A). Permutational multivariate analysis of variance (PERMANOVA) detected significant differences (*P* < 0.001) in genera composition along the transect and between depths for identified groups, as well as a significant effect of the interaction between these two factors on clustering patterns (Table 3). The similarity-percentage (SIMPER) analysis typified the genera that contributed to these differences along the transect and the most distinctive genera responsible for differences between depths (Tables S2 and S3). The mean dissimilarity in genera composition between the two groups along the transect (Fig. 3A) was 81% (Table S2). Twenty percent of the prokaryote genera (i.e., 58 out 285) contributed to 95% of the differences between groups along the transect; however, only five genera accounted for approximately 50% of these differences (i.e., *Alteromonas*, *Synechococcus*, *Cyanobium*, *Prochlorococcus*, and *Pseudomonas*) (Table S2). The mean dissimilarity in genera composition among depths (Fig. 3B) was 74%, and genera such as *Synechococcus*, *Cyanobium*, *Prochlorococcus*, and *Alteromonas* contributed approximately 54% to these differences (Table S3).

**TABLE 3:** Summary of the two-way mixed-effect PERMANOVA to test the changes in prokaryote community composition and CWM of traits across the transect and over the depth^*a*^

Variable	Effect	SS	DF	MS	*F*
Genera composition	Station	2.892	5	0.578	26.583***
	Depth	0.643	1	0.643	29.56***
	Station × Depth	1.517	5	0.303	13.942***
	Residual	0.522	24	0.021	
	Total	5.575	35		
CWM of traits	Station	166.407	5	33.281	58.727***
	Depth	54.961	1	54.961	96.983***
	Station × Depth	63.566	5	12.713	22.434***
	Residual	13.601	24	0.566	
	Total	298.54	35		

^*a*^ SS, sum of square; DF, degree of freedom; MS, mean square; *F, F*-statistic. The level of statistical significance for each effect is indicated by asterisks (***P < 0.001). The *P*-values were obtained after 9,999 permutations.

**Fig 4:**
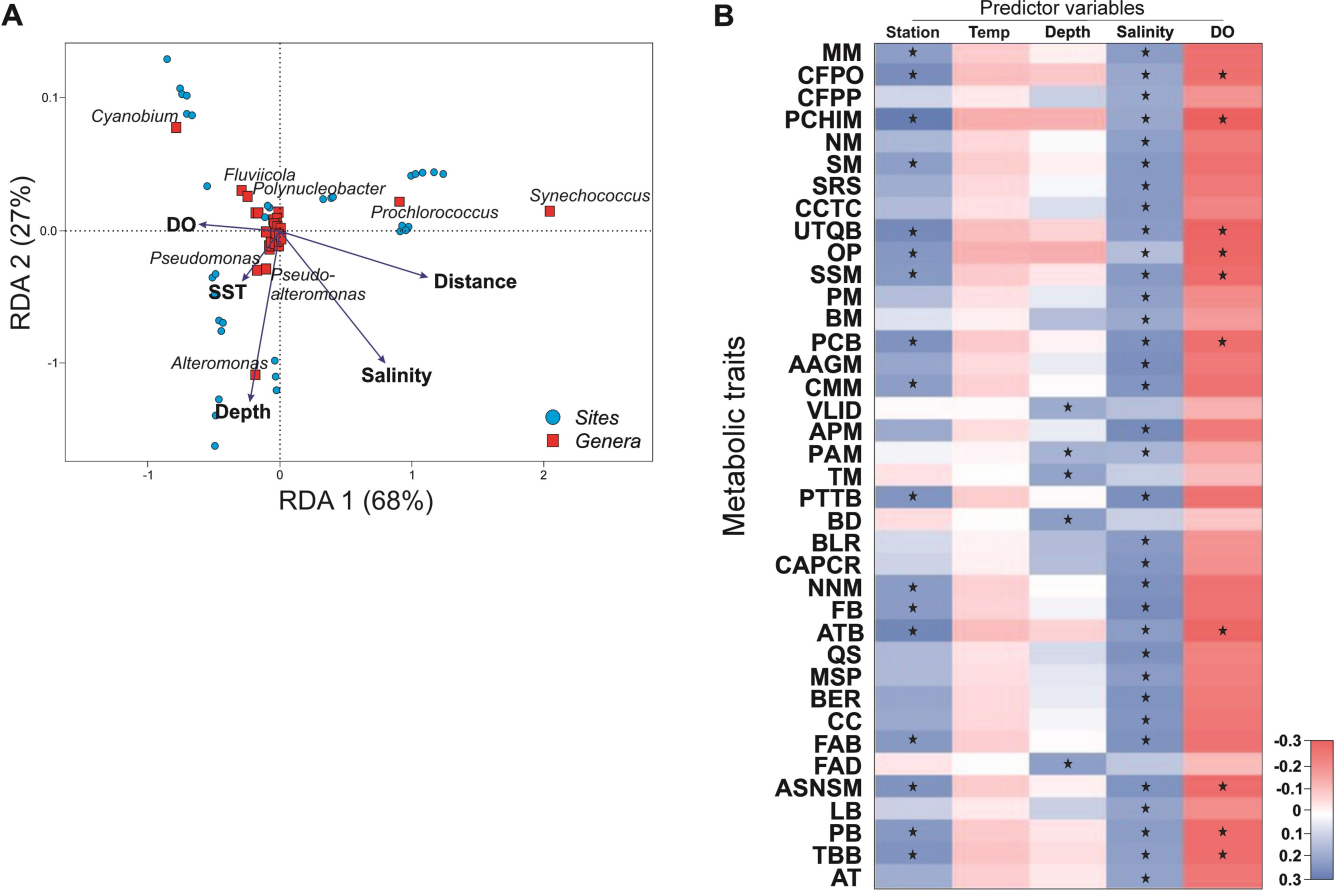
Distance-based dbRDA plot showing the relationship between genera composition (in red) and environmental variables in the studied sites (in blue) and fourth-corner analysis for variable-trait combinations. (**A**) The dbRDA ordination plot is based on the Hellinger distance of prokaryote genus abundance and illustrates the sample and genus scores from triplicate samples plotted against forward-selected environmental variables. (**B**) Traits and their acronyms in the fourth-corner plot are described and defined in Table 4, respectively. Fourth-corner coefficients for traits are shown in blue for a positive trait-variable association and in red for a negative association. Significant variable-trait associations are indicated with a star (*P* < 0.05). DO, dissolved oxygen.

The distance-based redundancy analysis (dbRDA) indicated that the constraining variables explained 63% of the variance in prokaryote community composition (Fig. 4A). Temperature was excluded from the model after performing a stepwise forward variable selection, and the highest scores explained 62% of the variability in taxonomic structure (*P*_adjusted_ ≤ 0.05). In addition, the first two axes of the dbRDA plot explained 95% of the data variability, while the remaining axes explained 5% of the variance. The sampling station (i.e., distance along the transect) was the main variable that constrained the prokaryote community structure, explaining 30% of the total variation (pseudo-*F* = 24.35; *P*_adjusted_ = 0.01). This was followed by depth (16%), salinity (10%), and dissolved oxygen (7%) (Fig. 4A).

### Metabolic traits and functional diversity of prokaryote estuarine communities

A total of 285 prokaryote genera with different extents of contribution to selected metabolic traits were used to compute functional diversity descriptors. Significant differences in functional richness among depths were detected (Table 2), with higher functional richness for aphotic samples ($\overset{-}{x}$~ 0.37 ± 0.10) than photic ones ($\overset{-}{x}$ ~ 0.27 ± 0.17) (Fig. 2B). This pattern was associated with depth-related differences in the amount of functional space occupied by the members of prokaryote communities (Fig. S3 and S4). The functional space ($\overset{-}{x}$ ~ 0.32 ± .14) did not record significant changes along the transect, indicating that the sampling station (as a predictor factor) did not have a significant effect on this facet (Table 2). The functional mean pairwise distance (FMPD), which estimates functional similarity between genera, tended to increase significantly along the transect at the two depths (Fig. 2C), which was also associated with a significant effect of the interaction between tested factors (station × depth) on FMPD (Table 2). This trend was clear from the St7 to the open ocean (Fig. 2C), with differences between photic and aphotic samples associated with higher scores at 0.5 m ($\overset{-}{x}$ ~ 0.25 ± 0.03) compared to 11 m depth ($\overset{-}{x}$ ~ 0.22 ± 0.03) (Table S1). Functional redundancy was not significantly affected by the interaction between depth and station (Table 2), but it decreased significantly along the transect (Fig. 2D), with photic samples (0.5 m) showing higher values ($\overset{-}{x}$ ~ 0.48 ± 0.06) compared to bottom samples ($\overset{-}{x}$ ~ 0.30 ± 0.06). If functional evenness represents how traits are distributed in a niche space, then lower evenness values would reflect an under-utilized niche (Fig. 2E). We only detected significant differences along the transect (Table 2) given by erratic fluctuation, without a clear trend (Fig. 2E). However, mean values of functional evenness ($\overset{-}{x}$ ~ 0.44 ± 0.06) (Table S1) indicated a regular trait distribution along the functional space at each sampling site and along the transect (Fig. S3 and S4). In addition, functional turnover covaried between depths along the transect, while the strong drop of the functional β-diversity at St4-St7 (Fig. 2F) indicated that prokaryote communities exhibited important changes in trait composition between both stations.

The NMDS ordination based on community-weighted means (CWMs) of traits clustered samples along the transect and among depths (Fig. 3C and D), following the composition patterns described above (Fig. 3A and B). PERMANOVA (based on trait CWMs) also indicated significant differences (*P* < 0.001) between resulting clusters along the transect and over depth (Table 3). The SIMPER analysis typified metabolic traits that contributed significantly to the dissimilarity among groups of samples along the transect and among depths (Tables S4 and S5). The average dissimilarity of groups of samples along the transect was 8.33% (Table S4). In fact, 10 traits (i.e., porphyrin and chlorophyll metabolism [PCHlM]; benzoate degradation [BD]; valine, leucine, and isoleucine degradation [VLID]; carbon fixation pathways in prokaryotes [CFPP]; butanoate metabolism [BM]; oxidative phosphorylation [OP]; fatty acid degradation [FAD]; tryptophan metabolism [TM]; lipopolysaccharide biosynthesis [LB]; and pyruvate metabolism [PM]) (Table 4) contributed to approximately 54% of these differences. The mean dissimilarity between samples over depth driven by differences in trait composition was 8.14%, but only 26% of the traits (i.e., PCHlM, OP, BD, VLID, cationic antimicrobial peptide CAMP resistance [CAPCR], BM, aminoacyl tRNA biosynthesis [ATB], carbon fixation in photosynthetic organisms [CFPO], quorum sensing [QS], and beta lactam resistance [BLR]) accounted for 52% of these differences (Table S5).

**TABLE 4:** Metabolic traits used to compute functional diversity facets^*a*^

Category	Trait	Acronym	Biological role
Biogeochemical cycle	Methane metabolism	MM	Methane is metabolized principally by methanotrophs and methanogens in the global carbon cycle
	Carbon fixation in photosynthetic organisms	CFPO	Inorganic carbon fixation by living organisms from the atmosphere to generate organic compounds
	Carbon fixation pathways in prokaryotes	CFPP	Carbon fixation is an important pathway for autotrophs. Cyanobacteria fix CO_2_ into organic compounds via Calvin cycle. Autotrophic prokaryotes can perform carbon fixation by using five additional pathways
	Porphyrin and chlorophyll metabolism	PCHlM	Reactions involved in the synthesis, use, and/or degradation porphyrins (chemical compounds with the backbone ring structure consisting of four linked pyrrole units) and chlorophyll
	Nitrogen metabolism	NM	Biological process carried out by many microorganisms involving different reactions where nitrogen is varying between various oxidation states. Nitrogen is an essential component of all living organisms
	Sulfur metabolism	SM	The metabolism of sulfur compounds plays an important role in the global sulfur cycle and is essential for life. Microorganisms drive sulfur transformations. For instance, the capacity for oxidation of sulfur is quite widespread among prokaryotes
	Sulfur relay system	SRS	Prokaryotic sulfur-relay enzymes control a wide range of physiological processes based on the transfer of sulfur and multiple sulfur mediators. For instance, the biogenesis of 2-thiouridine derivatives at the wobble position 34 of bacterial tRNA requires a sulfur-relay system
Metabolic process	Citrate cycle TCA cycle	CCTC	The TCA cycle (or Krebs cycle) is the final step for aerobic respiration of carbohydrates and fatty acids and generates energy and reducing power
	Ubiquinone and other terpenoid quinone biosynthesis	UTQB	Electron carriers involved in oxidative phosphorylation and photosynthesis
	Oxidative phosphorylation	OP	The phosphorylation of ADP to ATP coupled to oxidation of a metabolite through the respiratory chain. This leads to a proton gradient across the membrane and ATP synthesis
	Starch and sucrose metabolism	SSM	Metabolic capacities (e.g., degradation) of prokaryotes based on carbohydrate metabolism pathways
	Pyruvate metabolism	PM	Metabolic capacities based on pyruvate as a key intermediate in energy metabolism of prokaryotes
	Butanoate metabolism	BM	The chemical reactions and pathways involving any butyrate, the anions of butyric acid (butanoic acid), a saturated unbranched aliphatic acid
	Pantothenate and CoA biosynthesis	PCB	Pantothenate (vitamin B5) is the key precursor for the biosynthesis of coenzyme A, which is an essential cofactor in prokaryotes and all living organisms
Amino acid metabolism	Alanine, aspartate, and glutamate metabolism	AAGM	These are the most common D-amino acids in living organisms including prokaryotes. They can support microbial growth
	Cysteine and methionine metabolism	CMM	Cysteine derivatives are important for protection against the oxidative stress in bacteria, while methionine is an essential amino acid
	Valine, leucine, and isoleucine degradation	VLID	Metabolic reactions involved in the degradation of valine, leucine, and isoleucine
	Arginine and proline metabolism	APM	Arginine participates widely in metabolic processes. For instance, Anabaena catabolizes arginine to produce proline and glutamate, with concomitant release of ammonium, as major products
	Phenylalanine metabolism	PAM	Metabolic reactions involved in the degradation and biosynthesis of phenylalanine. This is utilized to synthesize proteins
	Tryptophan metabolism	TM	Many bacteria can metabolize tryptophan. For instance, it is incorporated into bacterial enzymes and serves as a precursor of the cofactor NAD
	Phenylalanine, tyrosine, and tryptophan biosynthesis	PTTB	Phenylalanine, tyrosine, and tryptophan are aromatic amino acids involved in protein synthesis
Chemical degradation	Benzoate degradation	BD	Most bacteria are capable of benzoate degradation as a sign of the ability to degrade aromatic compounds
	Beta lactam resistance	BLR	The beta-lactam antibiotics are the most widely used antibiotic family. Bacterial resistance to beta-lactams can be achieved by different mechanisms
	Cationic antimicrobial peptide CAMP resistance	CAPCR	Cationic antimicrobial peptides have important roles in microbial ecology and defense of higher hosts. Many bacteria have developed strategies to counteract the efficacy of CAMPs such as defensins and bacteriocins
Biological process	Nicotinate and nicotinamide metabolism	NNM	NAD is a coenzyme for redox reactions and a substrate of NAD-dependent enzymes that are involved in a variety of cell processes
	Folate biosynthesis	FB	Prokaryotic cells require folate cofactors for the biosynthesis of diverse components
	Aminoacyl tRNA biosynthesis	ATB	The synthesis of aminoacyl-tRNAs is a ubiquitous cellular process that provides the substrates for translation
	Quorum sensing	QS	Quorum sensing is a cell-to-cell communication process that allows bacteria to share information about cell density and gene expression to generate and regulate different phenotypes (e.g., virulence and biofilm formation)
	mRNA surveillance pathway	MSP	Is a quality control mechanism that detects and degrades defective mRNAs
	Base excision repair	BER	Is a mechanism that corrects small DNA lesions
	Cell cycle	CC	Cell cycle progression (e.g., division cycle, DNA replication)
Cell wall	Fatty acid biosynthesis	FAB	The reactions of fatty acid biosynthesis are essential, and the main fate of these macromolecules in prokaryotes is the formation of membrane lipids
	Fatty acid degradation	FAD	Fatty acid degradation yields acetyl-coenzyme A (CoA) that can be metabolized to obtain energy and precursors for biosynthesis pathways
	Amino sugar and nucleotide sugar metabolism	ASNSM	Prokaryotes can take up and utilize various sugars for either glycolysis or cell wall biosynthesis
	Lipopolysaccharide biosynthesis	LB	The LPS biosynthesis affects the cell membrane assembly and many features of prokaryotes such as cell survival, virulence, cell surface hydrophobicity, among others
	Peptidoglycan biosynthesis	PB	Metabolic reactions involved in the polymerization of the major structural component in most prokaryotic cell walls
	Terpenoid backbone biosynthesis	TBB	Terpenoids or isoprenoids are a class of natural products consisting of isoprene units. In cyanobacteria, they are synthesized from the methylerythritol-phosphate pathway
	ABC transporters	AT	ATP-binding cassette (ABC) transporters couple ATP hydrolysis with the uptake and outflow of solutes through the prokaryote cell membranes

^*a*^ Functional categories, acronyms, and the biological role (and/or relevance) of each trait are given.

A fourth-corner analysis indicated that all functional traits showed significant interactions with tested environmental variables, except for temperature (Fig. 4B). Salinity and sampling station exhibited statistically significant (*P*_adjusted_ < 0.05) positive correlations with 89% and approximately 45% of the metabolic traits, respectively (Fig. 4B) (Table S6). Significant positive associations between salinity and CWM of the metabolic traits indicated that most traits increased in relative importance from the head of the estuary toward the ocean (Fig. 4B), which was especially true for alanine, aspartate, and glutamate metabolism (AAGM); arginine and proline metabolism (APM); phenylalanine, tyrosine, and tryptophan biosynthesis (PTTB); folate biosynthesis (FB);and fatty acid biosynthesis (FAB). Similarly, 17 traits showed a positive association between station and CWMs, indicating that the relative importance of traits such as CFPO; PCHlM; ubiquinone and other terpenoid quinone biosynthesis (UTQB); pantothenate and CoA biosynthesis (PCB); and ATB increased from the head of the estuary toward the open ocean (Fig. 4B). The relative importance of traits such as VLID, PM, TM, BD, and FAD (Table 4) increased with increasing depth (Fig. 4B). In turn, significant negative associations were found between dissolved oxygen and traits CFPO, PCHlM, UTQB, OP, starch and sucrose metabolism (SSM), PCB, ATB, amino sugar and nucleotide sugar metabolism (ASNSM), peptidoglycan biosynthesis (PB), and terpenoid backbone biosynthesis (TBB) (Table S6).

## DISCUSSION

### Prokaryote estuarine communities

The ecosystem variability along estuarine salinity gradients plays an important role in shaping spatial patterns of physical properties, biogeochemical cycles, and biota at different levels of biological organization, from microbes and eukaryotes to multispecies networks (41). While brackish-water environments like tropical estuaries and coastal lagoons can exhibit a decreasing trend in species richness of macrobiota with an increase in salinity (42, 43), changes in microbial richness can deviate from this pattern in response to drivers such as niche availability, community mixture from freshwater and seawater with autochthonous brackish communities, and/or ecosystem disturbances (13, 44).

The footprint of river runoff in the Urabá Estuary was detectable as a salinity gradient that increased along a transect from the southernmost part of the estuary toward the open ocean (Fig. 1). We detected a slight decreasing trend in the average genera richness of prokaryote communities (Fig. 2A) with increasing salinity along the estuarine transect (Fig. S1B). This result aligns with findings across a salinity gradient in the Delaware Bay, where the highest and lowest bacterial richness were detected at low and high salinity, respectively (45). However, studies with higher spatio-temporal resolution are needed to clarify the trend in genera richness detected along the estuarine gradient in the Gulf of Urabá. For instance, a study conducted along a 2,000 km salinity gradient (from ~0 to 31 psu) in the Baltic Sea found no discernible trends in both the number of bacterial OTUs and the Shannon index across the transect (46). At the phyla level, the top 10 taxonomic groups in decreasing order of abundance in the Urabá Estuary were Proteobacteria, Cyanobacteria, Bacteroidetes, Marinimicrobia (SAR406_clade), Chloroflexi, Verrucomicrobia, Planctomycetes, Actinobacteria, Nitrospinae, and Margulisbacteria (data not shown). The dominance of Proteobacteria and other abundant phyla (e.g., Bacteroidetes, Actinobacteria, Cyanobacteria, Planctomycetes, and Verrucomicrobia) followed a biogeographic pattern previously described in estuarine environments from Columbia, Delaware, Jiulong, Pearl, and Hangzhou, among others (16). Alphaproteobacteria and Betaproteobacteriales showed similar abundances in the two depths analyzed in our study, but the former dominated toward the open ocean. Betaproteobacteriales tended to increase in abundance in the central part of the estuary (St7). In turn, surface Gammaproteobacteria dominated in the central part of the Urabá Estuary and, in the bottom layer, tended to increase in abundance toward the open ocean (data not shown). Similarly, a compositional succession of heterotrophic bacteria has been reported in two temperate estuaries of the Chesapeake Bay; these changes were characterized by a dominance of Alphaproteobacteria in saltwater areas and Betaproteobacteria in freshwater-influenced areas in addition to sporadic peaks of Gammaproteobacteria along the estuarine transects (47). The abundances of orders such as *Flavobacteriales* and *Rhodobacterales* in the Urabá Estuary (Fig. S2) were similar to those determined in marine samples from the Caribbean Sea near Curaçao (16S rRNA gene databases available at https://www.gbif.org/dataset/454cba05-03a6-4947-84a0-56c7e75d565b), while *Synechococcales* and *Rhodospirillales* were less abundant (48) compared to our study area. In addition, recent findings on coral reef microbiomes in Curaçao (49) aligned with our results by reporting communities primarily composed of taxa belonging to *Alphaproteobacteria* (with several SAR11 clades), *Gammaproteobacteria*, *Bacteroidetes*, and *Actinobacteria*, as well as the important presence of *Synechococcales*.

Significant differences in prokaryote community composition were detected along horizontal and vertical environmental gradients in the Urabá Estuary. Samples from St2 to St7 and St9 to St15 formed two significantly distinct groups along the estuarine transect in terms of genera composition (Fig. 3A); the same was observed between samples collected from the surface and bottom layers (Fig. 3B) (Table 3). These results were consistent with previous studies that reported large differences in prokaryote community networks along salinity gradients (50) and at small vertical-scale profiles in estuaries (51). Bacteria of the gammaproteobacterial genera *Alteromonas* and *Pseudomonas*, along with members of *Synechococcus*, *Cyanobium*, and *Prochlorococcus*, together contributed to 50% of the differences between the two identified groups along the estuarine transect (Table S2). Similarly, the three cyanobacteria plus *Alteromonas* together contributed approximately 54% of the total compositional differences between surface and bottom samples (Table S3) (Fig. 3B). Cultivable representatives of *Alteromonas* and *Pseudomonas* have recently been reported in deep-sea sediments from the Colombian Caribbean Sea as potential hydrocarbon degraders (52). The main explanatory variables of the total variation in prokaryote community composition were distance (30%), depth (16%), salinity (10%), and oxygen (7%) (Fig. 4A). The changes explained by distance and depth could be an indirect reflection of the overall environmental variability in the horizontal and vertical planes of the water column in the Urabá Estuary, which could depend on changes in variables other than those measured in our study (e.g., organic matter, chl-a, nutrients). Here, the variability of genera such as *Alteromonas*, *Pseudomonas*, and *Pseudoalteromonas* was mostly associated with depth (Fig. 4A), suggesting that vertical organic matter gradients may be important in the Urabá Estuary (53). Indeed, it has been reported that members of the genus *Alteromonas* are potentially important copiotrophs in the carbon cycle of most marine environments (54) by using *r*-strategies for niche differentiation (55). In addition, bacteria of the genus *Pseudomonas* are widely distributed in marine and estuarine environments (56, 57); they have been considered indicators of pathogenic risk (58, 59) and are linked to environmental pollution and hydrocarbon degradation activity (60), as well as to the spread of multidrug resistance in estuaries (61).

The genus *Synechococcus* was one of the most abundant members of *Synechococcales*. This finding is consistent with previous studies showing a worldwide distribution of *Synechococcus* from estuaries to the open ocean and from equatorial to polar latitudes (62, 63). Furthermore, *Prochlorococcus* was mainly detected between St9 and St15, which is consistent with its biogeographic distribution in oceanic areas (64). The presence of *Prochlorococcus* in the bottom waters of St4 was likely associated with the intrusion of the marine plume towards the head of the estuary (Fig. S1). The dbRDA analysis (Fig. 4A) suggested that environmental factors other than those tested in the present study could explain the variability of *Prochlorococcus* and *Synechococcus* (64). Although this is the first report about the presence of both phototrophs on the Gulf of Urabá, these picocyanobacteria have previously been detected at the CARMABI reef and other Caribbean Sea areas (49, 65). Members of the genus *Cyanobium* have been considered ecologically important in different Caribbean regions (66, 67), but there are no previous records for this group in the Urabá Estuary. Here, this genus became dominant toward the head of the estuary, being detected at both depths along the estuarine transect (Fig. S3 and S4), but mostly at St2 and St4. Indeed, members of this genus are often associated with freshwater and low-salinity environments (68) and as being responsible for estuarine blooms explained mainly by changes in temperature and salinity (69). It has been reported that high temperature and low salinity are satisfactory descriptors of the overall abundance patterns of picocyanobacteria (70, 71). In the present study, the total variability of the genus *Cyanobium* was partially associated with variations in oxygen concentrations and low salinity, but not with temperature (Fig. 4A).

### Functional diversity of prokaryote communities

Similar to genera composition results, samples from St2 to St7 and St9 to St15 formed two significantly distinct clusters for CWMs along the transect and over depth (Fig. 3C and D) (Table 3). Ten traits together contributed between 52% and 54% of the differences between the two groups identified either along the transect or among depths (Tables S4 and S5). Five out of these 10 traits (i.e., PCHlM, VLID, OP, BD, and BM) drove the cluster differentiation in both water-column planes. The ubiquity of porphyrin in aquatic environments (72), which includes chlorophyll and products derived from its degradation, supports the involvement of reactions for the synthesis, utilization, and/or degradation of these compounds (PCHlM) in cluster differentiation. The contribution of complex amino acid degradation (VLID) and energetic metabolism (OP) to cluster differentiation was probably dependent on the availability (in quantity and quality) of dissolved organic matter as a substrate for microbial food webs in estuarine waters (7). In addition, benzoate degradation (BD), VLID, and other traits (e.g., phenylalanine metabolism [PAM], TM) were significantly positively associated with depth (Fig. 4B), suggesting a higher contribution of these attributes to light-independent microbial metabolism in the aphotic layer. For instance, aromatic hydrocarbons may contribute significantly to the sedimentary carbon budget in the Urabá Estuary (73) and most bacteria capable of BD, such as those belonging to *Alteromonas*, *Pseudomonas*, and *Pseudoalteromonas* (74–76), have the potential to degrade aromatic compounds.

No information exists about how estuarine environmental variability modulates the functional diversity of prokaryote communities. Clustering patterns were associated with an important drop in functional β-diversity between St4 and St7 (Fig. 2F), the weakening of the vertical stratification beyond St9, and its strengthening between St2 and St7, as influenced by freshwater inputs on the surface layer (Fig. S1). The stratification is largely explained by the Atrato River’s high discharges into the estuary, as revealed by previous field data and numerical modeling (77). In general, salinity is one of the major abiotic factors that govern multispecies assemblage life processes in estuarine environments (18, 78, 79). Indeed, salinity and other environmental factors (e.g., pH, dissolved oxygen, and nutrients) can explain the phytoplanktonic abundance in the Gulf of Urabá (26). In the present study, most of the metabolic traits studied at whole prokaryote community level were significantly and positively associated with salinity (34 out 38 traits) and sampling site (17 out 38 traits) (Fig. 4B). Autotrophic carbon fixation pathways (CFPO, CFPP) became more prominent with increasing salinity along the estuarine transect (Fig. 4B). This result is consistent with reports on increased expression levels of Calvin-cycle genes in brackish waters across an estuarine gradient in New Zealand (18), and higher dark carbon fixation rates at middle- and high-salinity zones compared to low-salinity zones in China’s Yangtze Estuary (80). In addition, heterotrophic CO_2_ fixation based on anaplerotic reactions is considered a significant process in estuaries with high organic matter inputs (7), an environmental condition also present in the Urabá Estuary (73), where the pyruvate pathway (PM), which uses some of the enzymes required for anaplerosis, had a higher presence at high salinity (Fig. 4B). The overall influence of each trait on prokaryote community functioning increased with increasing salinity, regardless of whether salinity had negative effects on specific community members, such as those that could be expected for representatives of the genus *Cyanobium* (81) and freshwater bacteria affiliated to genera *Fluviicola* (82) and *Polynucleobacter* (83) (Fig. 4A). While the modulating effect of salinity on the microbial community structure is well known, its role in the spread of antibiotic resistance requires further investigation (84). For example, there is contrasting evidence from estuarine sediments indicating that salinity may (85) or may not (59) be involved in shaping antibiotic resistance spread patterns. Other studies have reported that the combined effect of antibiotics and salinity may increase the selection pressure and, thus, promote the spread of antibiotic resistance genes in estuaries (86), which, in turn, has been linked to the adaptability of antibiotic-resistant bacteria during the migration from rivers to oceans (87). Consistent with these findings, a significant positive relationship between salinity and CAPCR or BLR was detected in the Urabá Estuary (Fig. 4B). In addition, the molecular processes involved in the regulation of bacterial phenotypes appeared to be favored at higher salinity. Indeed, BM with a recently recognized role in biofilm formation (88), and QS were significantly positively associated with salinity (Fig. 4B).

Dissolved oxygen was significantly negatively associated with ten metabolic traits (Fig. 4B). The negative relationship between this variable and CFPO could result from the balance of contributions from different carbon fixation pathways with different degrees of oxygen sensitivity, including the ubiquitous Calvin-Benson-Bassham cycle. Recent genomic evidence supports this notion, indicating that distinct autotrophic carbon fixation pathways may be differentially distributed along a redox gradient (from oxic to anoxic conditions) in oceanic waters (89). Bacterial metabolism often relies on the transfer of electrons to oxygen as the final electron acceptor during respiration. Consequently, traits closely associated with the degradation and/or biosynthesis of organic components in obligate aerobes, such as PCHlM, UTQB, OP, SSM, PCB, ATB, and PB (Fig. 4B), are expected to be strongly coupled to energetic metabolism and oxygen consumption (90).

Functional richness (Fig. 2B) and functional spaces (Fig. S3 and S4) were greater in deeper communities than in the surficial stratum. This observation aligns with the idea that ecosystem functioning enhances when prokaryote diversity is higher (37, 91). In fact, a tight covariation between genera richness and functional richness was observed at a 0.5 m depth (Fig. 2A and B), indicating that functional traits increased as genera diversity also increased in the surficial stratum. Moreover, it would be expected that any depletion in diversity would constrain ecosystem functioning if there is no functional redundancy in the relationship between diversity and functioning (92). Indeed, strict functional redundancy is expected to be sustained by “core functions” less sensitive to prokaryote diversity loss due to ecosystem disturbances. Such functions are shared by a wide range of microbes, as opposed to “narrow functions” performed by a few specialist microbes (93–95). Based on CWMs, which indicate the prevalence of a particular trait within a community, PCHlM, CFPP, OP, cysteine and methionine metabolism (CMM), QS, cell cycle (CC), and ASNSM (Fig. S5) are proposed as “core traits” of prokaryote communities in the Urabá Estuary, as well as candidate traits for assessing functional redundancy (Fig. 2D) in this area. However, partial functional redundancy for shared ecosystem functions among microbes with different ecological needs (e.g., PCHlM) that enable coexistence cannot be ruled out (96). Surficial communities held significantly higher functional redundancy than deeper communities (Fig. 2D). This facet of diversity also decreased significantly along the transect at both depths (Table 2). These results were likely related to specific trait combinations (e.g., PCHlM-CFPP-OP) that importantly contributed to the dissimilarity among samples (Tables S4 and S5) and were more recurrent in surficial communities and closer to the head of the Urabá Estuary than deeper communities in areas farther from this zone (Fig. S5). Lower functional evenness values toward open ocean, especially for the surface of St13 (Fig. 2E), indicated that some parts of the niche space were underutilized. This has been associated with lower ecosystem productivity and stronger selection pressure on rare functional traits (97), which in our case could be those with lower CWM scores (98), such as some of the traits in the cell wall category (see PB and TBB in Fig. S5).

### Conclusions

The taxonomic composition of the prokaryote communities in the Urabá Estuary during the sampling period was significantly influenced by environmental variability related to the sampling station along the transect (distance), depth, and salinity gradient. Sampling station and depth were the main explanatory variables, suggesting that variables other than those analyzed in our study would be important environmental drivers along the horizontal and vertical gradients. Consequently, two recommendations are given for future microbial studies in the Urabá Estuary (i) to expand the environmental variables to be studied by evaluating the effects of organic matter, dissolved organic carbon, nutrients, and Chlo-a and (ii) to improve temporal resolution. Temperature was not a significant explanatory variable for changes in the taxonomic and functional diversity of the prokaryote communities, including cyanobacteria. The major phyla present in the Urabá Estuary followed a pattern of abundance described in several estuarine environments elsewhere, with some taxonomic entities detected here also reported in the few sites where microbial data are available off the South American boundary of the Caribbean Sea. The genera *Alteromonas*, *Pseudomonas*, *Synechococcus*, *Cyanobium*, and *Prochlorococcus* jointly contributed nearly half of the total dissimilarity between sample clusters along the estuarine transect and among depths.

A trait‐based approach has seldom been applied to natural environments to determine functional diversity using 16S rRNA genes or metagenomic data (38, 96, 99, 100). Our study provides a guide for addressing new questions about the potential functional diversity of prokaryote communities in transitional aquatic ecosystems, such as the Urabá Estuary, and advances the field of multiple trait-based prokaryote community ecology. Our multi-trait approach was intended to assess ecosystem functioning by using traits ranging from core to cell fitness attributes and, thereby, to capture a broad range of functional diversity. In general, there was an increasing influence of traits on prokaryote community functioning with increasing salinity. However, functional facets (i.e., richness, evenness, FMPD, and redundancy) indicated a potential imbalance in functional diversity between surface communities closer to the head of the estuary and bottom communities closer to the open ocean. Indeed, five traits were the main contributors to the metabolic differentiation between the two groups identified along the transect (CFPP, FAD, TM, LB, and PM) and over depth (CAPCR, ATB, CFPO, QS, and BLR), in addition to PCHlM, VLID, OP, BD, and BM, which simultaneously contributed to this cluster differentiation in both planes of the water column. Finally, the higher functional redundancy in surface waters of the Urabá Estuary’s head (with respect to the ocean-influenced zone) is expected to be driven by specific trait combinations, such as PCHlM-CFPP-OP.

## MATERIALS AND METHODS

### Study area and sampling description

The Gulf of Urabá, spanning approximately 1,800 km^2^ with a mean depth of around 50 m, is characterized as a tropical estuary. It features a stratified water column and horizontal salinity gradients, primarily influenced by freshwater inflows from several rivers in the region, predominately the Atrato River, which merges with the saltier ocean waters (53). This estuary is situated in the southernmost part of the Colombian Caribbean (Fig. 1), encompassed within the geographic coordinates of 7°55′N–8°40′N and 76°53′W–77°23′W. The region predominately experiences two climatic periods, the dry season from December to April and the rainy season from May to November (101).

On 4 December 2019, the Urabá Estuary was surveyed. Triplicate water samples (800 mL each) were independently collected using 1.2 L Niskin water samplers (Model 1010; General Oceanics Inc., FL, USA) from six distinct sampling stations at depths of 0.5 and 11 m (Table 1). These stations were numbered in increasing order along the estuarine transect of almost 100 km, beginning at the head of the estuary at Station 2 (St2) and ending in the oceanic zone at St15 (Fig. 1). The present study visited the same sampling sites and used the same station numbering as in a previous study (26). All collected samples were stored in carboys prewashed with 10 N HCl. Samples were stored in darkness and maintained at surface water temperature throughout transport and until arrival at the coastal laboratory.

Temperature, salinity, and density data were collected from each sampling station with a CASTAWAY-CTD device (V1.50, Xylem Inc, CA, USA). Water transparency was gauged with a Secchi Disc, determining the aphotic layer’s depth at approximately 11 m. Dissolved oxygen concentrations were ascertained from triplicate water samples using the Winkler method (102).

### RNA extraction and complementary DNA synthesis

For RNA extraction, triplicate 100 mL water samples were filtered using sterilized 60 mL syringes and sterile 25 mm Swinnex filter holders for PVDF filters (0.22 µm pore size and 25 mm diameter; GVWP02500, Millipore). Post-filtration, the PVDF filters were air-dried and placed in sterile cryovials containing 300 µL of RNAlater solution (Ambion, Austin, TX, USA). These samples were then stored at −20°C until subsequent analysis.

During processing, the filters were gradually thawed on ice and extracted using the Ambion RNA Extraction Kit (AM1560, Ambion). The RNA extraction followed the manufacturer’s guidelines, incorporating an additional disruption step. This involved using 200 µm diameter zirconium beads (Low Binding Zirconium Beads, OPS Diagnostics, Lebanon, NJ) and the TissueLyser II device (Qiagen, Hilden, Germany) set for 2 × 1 min at a frequency of 27 Hz, with a 1 min pause between sessions. The RNA extracts’ concentration, quality, and integrity were assessed using a Qubit 4 fluorometer using the Qubit RNA HS and Qubit RNA IQ Assay Kits per the manufacturer’s instructions (Thermo Fisher Scientific, MA, USA).

All total RNA extracts underwent treatment with the TURBO DNA free Kit (Applied Biosystems, Austin, TX, USA) before initiating complementary DNA (cDNA) synthesis using the ImProm-II Reverse Transcription System, following the manufacturer’s protocol (Promega, WI, USA). The reverse transcription reactions utilized 10 ng of DNase-treated RNA and the random primer set supplied with the ImProm-II Reverse Transcription Kit. The resulting cDNAs were quantified with the Qubit ssDNA Assay Kit and then stored at −20°C, awaiting 16S rRNA gene amplicon sequencing.

### 16S amplicon-based sequencing and metabolic traits

Next-generation sequencing libraries and bioinformatic analyses were conducted at Genoma Mayor SpA (http://www.genomamayor.com/) to determine the taxonomic composition of both bacterial and archaeal communities. In brief, amplicon-based libraries were constructed from triplicate cDNA samples with the primer pair 515F-Y (5′-GTGYCAGCMGCCGCGGTAA) and 806RB (5′-GGACTACNVGGGTWTCTAAT) targeting the V4 hypervariable region of 16S rRNA genes. These primers were modified with specific Illumina adapters and barcodes, following standard protocols (http://www.earthmicrobiome.org/protocols-and-standards/16s/). PCRs utilized the 2 × KAPA HiFi HotStart Ready Mix (KAPA Biosystems), and PCR products were purified with AMPure XP (Beckman Coulter, CA, USA). PCR product size verification was undertaken using the DNF-900 Kit on a Fragment Bioanalyzer (Advanced Analytical Technologies Inc., IA, USA). This was done prior to quantification with the Quant-iT PicoGreen dsDNA Assay Kit (Invitrogen) on a HOEFER DQ300 fluorometer (Hoefer Inc., USA). DNA pooling and sequencing preparation used standard protocols, adhering to the Illumina Denature and Dilute Libraries Guide. Sequencing was executed on the Illumina HiSeq 250PE platform using approximately 100,000 reads per library. The raw sequence data can be accessed in the NCBI SRA database under the BioProject ID PRJNA988159.

For processing, low-quality sequences were filtered, and raw data underwent quality trimming using the DADA2 v1.26.0 R package (103). Based on the USEARCH method (104), quality-processed paired-end reads facilitated the generation of amplicon sequence variants via the DADA2 pipeline (103). Each amplicon sequence variant was identified using the Ribosomal Database Project Naive Bayesian Classifier algorithm, trained for V4 sequences of 16S rRNA genes (105). Data visualization employed the phyloseq package from the R package (106). An abundance × prokaryotic genus matrix for all samples was constructed by the sum of the read number of all amplicon sequence variants associated with the same genus. This matrix was divided per replicate, and the resulting matrices served as inputs for the iVikodak pipeline, adhering to the instructions available on the web platform (https://web.rniapps.net/iVikodak/). The Global Mapper module was set to “Co-metabolism”. A genus × trait matrix was obtained using a pathway exclusion cut-off (PEC) threshold of 80% (107). The PEC threshold is a filter that affects the functional inference process and allows iVikodak to report a pathway as “present” only if the proportion of its inferred constituent enzymes exceeds a minimum quorum (107) (80% in our case). The genus × trait matrix encompassed all prokaryote genera contributing collectively to each trait and their respective contribution values to metabolic traits. Contribution values can be defined as the computed abundance of a metabolic pathway (or trait) under the assumption that different microbes in a given environment drive the assigned pathway and collectively (but differentially) contribute to the functioning of that pathway. Traits were grouped into six functional categories: biogeochemical cycle, metabolic process, amino acid metabolism, chemical degradation, biological process, and cell wall (Table 4). Spearman’s correlation coefficients between traits (i.e., between contribution values of genera to traits) within the same functional category served as a trait selection criterion. Traits exhibiting lower contribution values and higher covariance with other traits of the same functional category were discarded. Biological relevance also played a role in trait selection, ensuring a balance between microbial characteristics, environmental preferences, and metabolic capabilities (37). Traits ranged from core attributes (e.g., tricarboxylic acid cycle and oxidative phosphorylation) to ecosystem functioning features (e.g., methane, porphyrin, and chlorophyll metabolism), even encompassing specific traits impacting cell fitness (e.g., chemical degradation and quorum sensing). Ultimately, 38 metabolic traits (Table 4) linked to 285 prokaryote genera were selected.

### Statistical analysis

Taxonomic diversity was calculated as the raw genus richness standardized by sample. In addition, we used five distinct functional diversity facets to evaluate the changes in trait distribution along the environmental gradient. First, functional richness (108, 109) represents the size of the functional space. It is estimated as the convex hull that encompasses the taxa in the community (110) and is subsequently represented with a principal component analysis ordination plot by four-axis reduction (109, 110). Second, FMPD (111) is the mean weighted distance between all taxa pairs. The index uses dendrogram branch lengths (clustering based on unweighted pair group method with arithmetic mean) estimated over a Gower Trait Distance Matrix (110, 112). This posits that lower FMPD scores indicate functionally closer communities (113). Third, functional redundancy is the difference between taxa diversity (using Simpson’s index) and the RaoQ index. This assumes that functional redundancy is the fraction of taxa diversity not explained by functional diversity (114–116). Fourth, the minimum spanning tree method calculates functional evenness, which evaluates taxa trait distribution in the functional space. This is so that values closer to one indicate a regular distribution of trait abundance in the functional space (109). Finally, the functional turnover was measured using the Jaccard’s dissimilarity index (116) between contiguous sampling stations along the estuary to detect how environmental variability influences the shifts in functional structure by using the average beta turnover as threshold value. To evaluate significant changes in taxonomic richness, functional richness, FMPD, functional redundancy, and functional evenness along the estuary, two-way Analysis Of Variances (ANOVAs) were implemented. This tested the effects of distance along the estuarine transect (sampling station) and among depths (0.5 and 11 m) (Fig. 1). Subsequent post-hoc analysis using Tukey’s Honest Significant Difference test detected significant differences between each factor’s levels. All these analyses were performed using the vegan package (117) in R v4.3.0 (118). Functional diversity parameters were obtained with the “mFD” package (110).

Furthermore, the community-weighted mean (CWM) was calculated to gauge the relative importance of each metabolic trait across prokaryote communities along the transect and between depths. The CWM is the average trait value of each genus by sampling station adjusted for abundance (33). This provides a dominance measurement, such that, the more abundant the genus, the greater their weight in the community (119). Community-weighted means were calculated using the R package “FD” (120). Changes in taxonomic composition and functional traits along the estuary were detected with NMDS ordination by using the Euclidian distance to detect clusters of samples separated by genus and CWMs of functional traits across the different stations and depths. PERMANOVA based on the Euclidean distance with 999 permutations (121) was implemented to evaluate significant changes in genera composition and CWMs between homogenous groups generated from NMDS ordinations along the transect and depths. SIMPER analyses were performed to identify traits and genera that contributed to the differences between treatments. A distance-based dbRDA was implemented with the Euclidean distance and combined with a stepwise forward selection function to explore linear correlations between prokaryote community composition and environmental variables (i.e., temperature, depth, station, salinity, and dissolved oxygen). This allows for the selection of the best environmental variables that explain most variations in community composition. Taxa abundances were Hellinger-transformed to improve linear ordinations, while the environmental variables were *Z*-score transformed for normality (122). The significance of the dbRDA model was tested using the “permutest” function in the vegan package of R with 999 permutations. The significance of a constrained axis and the effects and significances of each variable were also assessed, defining the “axis” and “margin” to avoid the sequential test of terms (123). All analyses were performed using the vegan package in R v4.3.0 (2023) (117). To quantify the relation between genera trait values (i.e., CWMs) and environmental variables, a fourth-corner analysis was implemented in a fourth matrix using three input matrices (i.e., R, abiotic variables; L, genera abundances; and Q, traits) (124). These analyses were performed using the “ade4” package (125) in the R environment v4.3.0 (2023).

## SUPPLEMENTAL MATERIAL

Supplemental material spectrum.03886-23-s0001.docx Tables S1 to S6; Fig. S1 to S5.

ASM does not own the copyrights to Supplemental Material that may be linked to, or accessed through, an article. The authors have granted ASM a non-exclusive, world-wide license to publish the Supplemental Material files. Please contact the corresponding author directly for reuse.

## References

[1] Woodwell GM, Rich PH, Mall CSA. 1973. Carbon in the biosphere. Proceedings of the 24th brookhaven symposium in biology. , p 221–240, USAEC, Springfield, Virginian

[2] Chilton D, Hamilton DP, Nagelkerken I, Cook P, Hipsey MR, Reid R, Sheaves M, Waltham NJ, Brookes J. 2021. Environmental flow requirements of estuaries: providing resilience to current and future climate and direct anthropogenic changes. Front Environ Sci 9:764218. doi:10.3389/fenvs.2021.764218

[3] Triassi M, Montuori P, Provvisiero DP, De Rosa E, Di Duca F, Sarnacchiaro P, Díez S. 2022. Occurrence and spatial-temporal distribution of atrazine and its metabolites in the aquatic environment of the Volturno River estuary, southern Italy. Sci Total Environ 803:149972. doi:10.1016/j.scitotenv.2021.149972 34482142

[4] Xing YN, Guo Y, Xie M, Shen RL, Zeng EY. 2009. Detection of DDT and its metabolites in two estuaries of South China using a SPME-based device: first report of p,p’-DDMU in water column. Environ Pollut 157:1382–1387. doi:10.1016/j.envpol.2008.11.034 19117651

[5] Jonkers N, Laane RWPM, de Voogt P. 2003. Fate of nonylphenol ethoxylates and their metabolites in two dutch estuaries: evidence of biodegradation in the field. Environ Sci Technol 37:321–327. doi:10.1021/es020121u 12564904

[6] Huang QY, Hou R, Xu R, Lin L, Li HX, Liu S, Qian PY, Cheng YY, Xu XR. 2024. Organophosphate flame retardants and their metabolites in the Pearl River Estuary: occurrence, influencing factors, and ecological risk control strategies based on a mass balance model. Environ Int 184:108478. doi:10.1016/j.envint.2024.108478 38330749

[7] Crump BC, Bowen JL. 2024. The microbial ecology of estuarine ecosystems. Annu Rev Mar Sci 16:335–360. doi:10.1146/annurev-marine-022123-101845 37418833

[8] Frankenbach S, Ezequiel J, Plecha S, Goessling JW, Vaz L, Kühl M, Dias JM, Vaz N, Serôdio J. 2020. Synoptic spatio-temporal variability of the photosynthetic productivity of microphytobenthos and phytoplankton in a tidal estuary. Front Mar Sci 7:170. doi:10.3389/fmars.2020.00170

[9] Qi L, Zheng Y, Hou L, Liu B, Zhou J, An Z, Wu L, Chen F, Lin Z, Yin G, Dong H, Li X, Liang X, Liu M. 2023. Potential response of dark carbon fixation to global warming in estuarine and coastal waters. Glob Change Biol 29:3821–3832. doi:10.1111/gcb.16702 37021604

[10] Braun A, Spona-Friedl M, Avramov M, Elsner M, Baltar F, Reinthaler T, Herndl GJ, Griebler C. 2021. Reviews and syntheses: heterotrophic fixation of inorganic carbon – significant but invisible flux in environmental carbon cycling. Biogeosciences 18:3689–3700. doi:10.5194/bg-18-3689-2021

[11] Signori CN, Valentin JL, Pollery RCG, Enrich-Prast A. 2018. Temporal variability of dark carbon fixation and bacterial production and their relation with environmental factors in a tropical estuarine system. Estuar Coast 41:1089–1101. doi:10.1007/s12237-017-0338-7

[12] Bauer JE, Bianchi TS. 2011. Dissolved organic carbon cycling and transformation, p 7–67. In Wolanski E, McLusky DS (ed), Treatise on estuarine and coastal science, 1st ed. Elsevier Academic Press, London, UK.

[13] Hewson I, Fuhrman JA. 2004. Richness and diversity of bacterioplankton species along an estuarine gradient in Moreton Bay, Australia. Appl Environ Microbiol 70:3425–3433. doi:10.1128/AEM.70.6.3425-3433.2004 15184140 PMC427769

[14] Levipan HA, Alarcón WO, Saldías GS. 2012. Fingerprinting analysis of the prokaryote community along a marine–freshwater transect in central-southern Chile. Ann Microbiol 62:1121–1140. doi:10.1007/s13213-011-0353-z

[15] Jeffries TC, Schmitz Fontes ML, Harrison DP, Van-Dongen-Vogels V, Eyre BD, Ralph PJ, Seymour JR. 2015. Bacterioplankton dynamics within a large anthropogenically impacted Urban Estuary. Front Microbiol 6:1438. doi:10.3389/fmicb.2015.01438 26858690 PMC4726783

[16] Ghosh A, Bhadury P. 2019. Exploring biogeographic patterns of bacterioplankton communities across global estuaries. Microbiologyopen 8:e00741. doi:10.1002/mbo3.741 30303297 PMC6528645

[17] Shah RM, Crosswell J, Metcalfe SS, Carlin G, Morrison PD, Karpe AV, Palombo EA, Steven ADL, Beale DJ. 2019. Influence of human activities on broad-scale estuarine-marine habitats using omics-based approaches applied to marine sediments. Microorganisms 7:419. doi:10.3390/microorganisms7100419 31590307 PMC6843362

[18] Tee HS, Waite D, Lear G, Handley KM. 2021. Microbial river-to-sea continuum: gradients in benthic and planktonic diversity, osmoregulation and nutrient cycling. Microbiome 9:190. doi:10.1186/s40168-021-01145-3 34544488 PMC8454136

[19] Ma F, Wang C, Zhang Y, Chen J, Xie R, Sun Z. 2022. Development of microbial indicators in ecological systems. Int J Environ Res Public Health 19:13888. doi:10.3390/ijerph192113888 36360768 PMC9654993

[20] Brankovits D, Pohlman JW, Niemann H, Leigh MB, Leewis MC, Becker KW, Iliffe TM, Alvarez F, Lehmann MF, Phillips B. 2017. Methane- and dissolved organic carbon-fueled microbial loop supports a tropical subterranean estuary ecosystem. Nat Commun 8:1835. doi:10.1038/s41467-017-01776-x 29180666 PMC5703975

[21] Anderson SR, Harvey EL. 2022. Estuarine microbial networks and relationships vary between environmentally distinct communities. PeerJ 10:e14005. doi:10.7717/peerj.14005 36157057 PMC9504456

[22] Vallejo Toro PP, Vásquez Bedoya LF, Correa ID, Bernal Franco GR, Alcántara-Carrió J, Palacio Baena JA. 2016. Impact of terrestrial mining and intensive agriculture in pollution of estuarine surface sediments: Spatial distribution of trace metals in the Gulf of Urabá, Colombia. Marine Pollution Bulletin 111:311–320. doi:10.1016/j.marpolbul.2016.06.093 27423441

[23] Toro López M, Van den Broeck P. 2021. Analysing (in) justice in the interplay of urbanisation and transport: the case of agrarian extractivism in the region of Urabá in Colombia. Quaest Geogr 40:35–61. doi:10.2478/quageo-2021-0011

[24] Velásquez FAG, Ramírez NJA, Urhán JB, Botero MT. 2008. Distribución de dos indicadores bacterianos de calidad de agua en el Golfo de Urabá, Caribe Colombiano. Gestión y Ambiente 11:87–95.

[25] Bonilla NS. 2020. Data from “comunidad fitoplanctónica presente en el Golfo de Urabá 2018". v1.0. Instituto de investigaciones ambientales del pacifico John Von Neumann (IIAP). Occurrence Dataset Repository. Available from: 10.15472/r1ln4m

[26] Córdoba-Mena N, Florez-Leiva L, Atehortúa L, Obando E. 2020. Changes in phytoplankton communities in a tropical estuary in the colombian Caribbean Sea. Estuaries Coast 43:2106–2127. doi:10.1007/s12237-020-00750-z

[27] Massing JC, Fahimipour AK, Bunse C, Pinhassi J, Gross T. 2023. Quantification of metabolic niche occupancy dynamics in a Baltic Sea bacterial community. mSystems 8:e0002823. doi:10.1128/msystems.00028-23 37255288 PMC10312292

[28] Bittleston LS, Freedman ZB, Bernardin JR, Grothjan JJ, Young EB, Record S, Baiser B, Gray SM. 2021. Exploring microbiome functional dynamics through space and time with trait-basedtheory. mSystems 6:e0053021. doi:10.1128/mSystems.00530-21 34427534 PMC8407496

[29] Díaz S, Cabido M. 2001. Vive la différence: plant functional diversity matters to ecosystem processes. Trends Ecol Evol 16:646–655. doi:10.1016/S0169-5347(01)02283-2

[30] Kraft NJB, Adler PB, Godoy O, James EC, Fuller S, Levine JM. 2015. Community assembly, coexistence and the environmental filtering metaphor. Funct Ecol 29:592–599. doi:10.1111/1365-2435.12345

[31] Lamanna C, Blonder B, Violle C, Kraft NJB, Sandel B, Šímová I, Donoghue JC II, Svenning J-C, McGill BJ, Boyle B, Buzzard V, Dolins S, Jørgensen PM, Marcuse-Kubitza A, Morueta-Holme N, Peet RK, Piel WH, Regetz J, Schildhauer M, Spencer N, Thiers B, Wiser SK, Enquist BJ. 2014. Functional trait space and the latitudinal diversity gradient. Proc Natl Acad Sci U S A 111:13745–13750. doi:10.1073/pnas.1317722111 25225365 PMC4183280

[32] Várbíró G, Borics G, Novais MH, Morais MM, Rimet F, Bouchez A, Tapolczai K, Bácsi I, Usseglio-Polatera P, B-Béres V. 2020. Environmental filtering and limiting similarity as main forces driving diatom community structure in mediterranean and continental temporary and perennial streams. Sci Total Environ 741:140459. doi:10.1016/j.scitotenv.2020.140459 32887020

[33] de Bello F, Lavorel S, Hallett LM, Valencia E, Garnier E, Roscher C, Conti L, Galland T, Goberna M, Májeková M, Montesinos-Navarro A, Pausas JG, Verdú M, E-Vojtkó A, Götzenberger L, Lepš J. 2021. Functional trait effects on ecosystem stability: assembling the jigsaw puzzle. Trends Ecol Evol 36:822–836. doi:10.1016/j.tree.2021.05.001 34088543

[34] Sapp J. 2005. The prokaryote-eukaryote dichotomy: meanings and mythology. Microbiol Mol Biol Rev 69:292–305. doi:10.1128/MMBR.69.2.292-305.2005 15944457 PMC1197417

[35] Krause S, Le Roux X, Niklaus PA, Van Bodegom PM, Lennon JT, Bertilsson S, Grossart H-P, Philippot L, Bodelier PLE. 2014. Trait-based approaches for understanding microbial biodiversity and ecosystem functioning. Front Microbiol 5:251. doi:10.3389/fmicb.2014.00251 24904563 PMC4033906

[36] Bewick S, Gurarie E, Weissman JL, Beattie J, Davati C, Flint R, Thielen P, Breitwieser F, Karig D, Fagan WF. 2019. Trait-based analysis of the human skin microbiome. Microbiome 7:101. doi:10.1186/s40168-019-0698-2 31277701 PMC6612184

[37] Escalas A, Hale L, Voordeckers JW, Yang Y, Firestone MK, Alvarez-Cohen L, Zhou J. 2019. Microbial functional diversity: from concepts to applications. Ecol Evol 9:12000–12016. doi:10.1002/ece3.5670 31695904 PMC6822047

[38] Johnson DR, Pomati F. 2020. A brief guide for the measurement and interpretation of microbial functional diversity. Environ Microbiol 22:3039–3048. doi:10.1111/1462-2920.15147 32608092

[39] Liu L, Wang S, Chen J. 2021. Anthropogenic activities change the relationship between microbial community taxonomic composition and functional attributes. Environ Microbiol 23:6663–6675. doi:10.1111/1462-2920.15702 34347346

[40] Shah RM, Stephenson S, Crosswell J, Gorman D, Hillyer KE, Palombo EA, Jones OAH, Cook S, Bodrossy L, van de Kamp J, Walsh TK, Bissett A, Steven ADL, Beale DJ. 2022. Omics-based ecosurveillance uncovers the influence of estuarine macrophytes on sediment microbial function and metabolic redundancy in a tropical ecosystem. Sci Total Environ 809:151175. doi:10.1016/j.scitotenv.2021.151175 34699819

[41] Cloern JE, Jassby AD, Schraga TS, Nejad E, Martin C. 2017. Ecosystem variability along the estuarine salinity gradient: examples from long‐term study of San Francisco Bay. Limnol Oceanogr 62:S272–S291. doi:10.1002/lno.10537

[42] Sosa‐López A, Mouillot D, Ramos‐Miranda J, Flores‐Hernandez D, Chi TD. 2007. Fish species richness decreases with salinity in tropical coastal lagoons. J Biogeogr 34:52–61. doi:10.1111/j.1365-2699.2006.01588.x

[43] Rahman MK, Hossain MB, Majumdar PR, Mustafa MG, Noman MA, Albeshr MF, Bhat EA, Arai T. 2022. Macrobenthic assemblages, distribution and functional guilds from a freshwater-dominated tropical estuary. Diversity 14:473. doi:10.3390/d14060473

[44] Parvathi A, Catena M, Jasna V, Phadke N, Gogate N. 2021. Influence of hydrological factors on bacterial community structure in a tropical monsoonal estuary in India. Environ Sci Pollut Res 28:50579–50592. doi:10.1007/s11356-021-14263-0 33963997

[45] Campbell BJ, Kirchman DL. 2013. Bacterial diversity, community structure and potential growth rates along an estuarine salinity gradient. ISME J 7:210–220. doi:10.1038/ismej.2012.93 22895159 PMC3526181

[46] Herlemann DP, Labrenz M, Jürgens K, Bertilsson S, Waniek JJ, Andersson AF. 2011. Transitions in bacterial communities along the 2000 km salinity gradient of the Baltic Sea. ISME J 5:1571–1579. doi:10.1038/ismej.2011.41 21472016 PMC3176514

[47] Bouvier TC, del Giorgio PA. 2002. Compositional changes in free‐living bacterial communities along a salinity gradient in two temperate estuaries. Limnol Oceanogr 47:453–470. doi:10.4319/lo.2002.47.2.0453

[48] Choi CJ, Bachy C, Jaeger GS, Poirier C, Sudek L, Sarma V, Mahadevan A, Giovannoni SJ, Worden AZ. 2017. Newly discovered deep-branching marine plastid lineages are numerically rare but globally distributed. Curr Biol 27:R15–R16. doi:10.1016/j.cub.2016.11.032 28073013

[49] Hoadley KD, Hamilton M, Poirier CL, Choi CJ, Yung C-M, Worden AZ. 2021. Selective uptake of pelagic microbial community members by caribbean reef corals. Appl Environ Microbiol 87:e03175-20. doi:10.1128/AEM.03175-20 33674432 PMC8091028

[50] Wang H, Chen F, Zhang C, Wang M, Kan J. 2021. Estuarine gradients dictate spatiotemporal variations of microbiome networks in the Chesapeake Bay. Environ Microbiome 16:22. doi:10.1186/s40793-021-00392-z 34838139 PMC8627074

[51] Da Silva RRP, White CA, Bowman JP, Bodrossy L, Bissett A, Revill A, Eriksen R, Ross DJ. 2022. Network and machine learning analyses of estuarine microbial communities along a freshwater-marine mixed gradient. Estuar Coast Shelf Sci 277:108026. doi:10.1016/j.ecss.2022.108026

[52] Blandón LM, Marín MA, Quintero M, Jutinico-Shubach LM, Montoya-Giraldo M, Santos-Acevedo M, Gómez-León J. 2022. Diversity of cultivable bacteria from deep-sea sediments of the colombian caribbean and their potential in bioremediation. Antonie Van Leeuwenhoek 115:421–431. doi:10.1007/s10482-021-01706-4 35066712

[53] Contreras-Fernández S, Florez-Leiva L, Bernal-Sánchez MC, Pacheco-Paternina W, Bedoya-Valestt S, Portillo-Cogollo L. 2022. Gulf of Urabá (Caribbean Colombia), a tropical estuary: a review with some general lessons About how it works. Ocean Sci J 57:556–575. doi:10.1007/s12601-022-00093-9

[54] Henríquez-Castillo C, Plominsky AM, Ramírez-Flandes S, Bertagnolli AD, Stewart FJ, Ulloa O. 2022. Metaomics unveils the contribution of alteromonas bacteria to carbon cycling in marine oxygen minimum zones. Front Mar Sci 9:993667. doi:10.3389/fmars.2022.993667

[55] Math RK, Jin HM, Kim JM, Hahn Y, Park W, Madsen EL, Jeon CO. 2012. Comparative genomics reveals adaptation by alteromonas sp. SN2 to marine tidal-flat conditions: cold tolerance and aromatic hydrocarbon metabolism. PLoS One 7:e35784. doi:10.1371/journal.pone.0035784 22563400 PMC3338528

[56] Kimata N, Nishino T, Suzuki S, Kogure K. 2004. Pseudomonas aeruginosa isolated from marine environments in Tokyo Bay. Microb Ecol 47:41–47. doi:10.1007/s00248-003-1032-9 15259268

[57] Diaz KE, Remold SK, Onyiri O, Bozeman M, Raymond PA, Turner PE. 2018. Generalized growth of estuarine, household and clinical Isolates of Pseudomonas aeruginosa. Front Microbiol 9:305. doi:10.3389/fmicb.2018.00305 29599754 PMC5863524

[58] Ghaderpour A, Mohd Nasori KN, Chew LL, Chong VC, Thong KL, Chai LC. 2014. Detection of multiple potentially pathogenic bacteria in matang mangrove estuaries, Malaysia. Mar Pollut Bull 83:324–330. doi:10.1016/j.marpolbul.2014.04.029 24820641

[59] Huang J, Zhu J, Liu S, Luo Y, Zhao R, Guo F, Li B. 2022. Estuarine salinity gradient governs sedimentary bacterial community but not antibiotic resistance gene profile. Sci Total Environ 806:151390. doi:10.1016/j.scitotenv.2021.151390 34740654

[60] Adebusoye SA, Amund OO, Ilori MO, Domeih DO, Okpuzor J. 2008. Growth and biosurfactant synthesis by nigerian hydrocarbon-degrading estuarine bacteria. Rev Biol Trop 56:1603–1611. doi:10.15517/rbt.v56i4.5746 19419068

[61] Hobeika W, Gaschet M, Ploy MC, Buelow E, Sarkis DK, Dagot C. 2022. Resistome diversity and dissemination of WHO priority antibiotic resistant pathogens in lebanese estuaries. Antibiotics (Basel) 11:306. doi:10.3390/antibiotics11030306 35326767 PMC8944630

[62] Partensky F, Hess WR, Vaulot D. 1999. Prochlorococcus, a marine photosynthetic prokaryote of global significance. Microbiol Mol Biol Rev 63:106–127. doi:10.1128/MMBR.63.1.106-127.1999 10066832 PMC98958

[63] Zwirglmaier K, Jardillier L, Ostrowski M, Mazard S, Garczarek L, Vaulot D, Not F, Massana R, Ulloa O, Scanlan DJ. 2008. Global phylogeography of marine Synechococcus and Prochlorococcus reveals a distinct partitioning of lineages among oceanic biomes. Environ Microbiol 10:147–161. doi:10.1111/j.1462-2920.2007.01440.x 17900271

[64] Flombaum P, Gallegos JL, Gordillo RA, Rincón J, Zabala LL, Jiao N, Karl DM, Li WKW, Lomas MW, Veneziano D, Vera CS, Vrugt JA, Martiny AC. 2013. Present and future global distributions of the marine cyanobacteria Prochlorococcus and Synechococcus*.* Proc Natl Acad Sci U S A 110:9824–9829. doi:10.1073/pnas.1307701110 23703908 PMC3683724

[65] Dudek KL, Cruz BN, Polidoro B, Neuer S. 2020. Microbial colonization of microplastics in the Caribbean Sea. Limnol Oceanogr Lett 5:5–17. doi:10.1002/lol2.10141

[66] Rejmánková E, Komárek J, Komárková J. 2004. Cyanobacteria — a neglected component of biodiversity: patterns of species diversity in inland marshes of northern Belize (Central America). Diversity Distrib 10:189–199. doi:10.1111/j.1366-9516.2004.00077.x

[67] Weber L, González-Díaz P, Armenteros M, Ferrer VM, Bretos F, Bartels E, Santoro AE, Apprill A. 2020. Microbial signatures of protected and impacted Northern Caribbean reefs: changes from Cuba to the Florida Keys. Environ Microbiol 22:499–519. doi:10.1111/1462-2920.14870 31743949 PMC6972988

[68] Celepli N, Sundh J, Ekman M, Dupont CL, Yooseph S, Bergman B, Ininbergs K. 2017. Meta-omic analyses of Baltic Sea cyanobacteria: diversity, community structure and salt acclimation. Environ Microbiol 19:673–686. doi:10.1111/1462-2920.13592 27871145

[69] Reyna NE, Hardison AK, Liu Z. 2017. Influence of major storm events on the quantity and composition of particulate organic matter and the phytoplankton community in a subtropical estuary, Texas. Front Mar Sci 4:43. doi:10.3389/fmars.2017.00043

[70] Cai H, Wang K, Huang S, Jiao N, Chen F. 2010. Distinct patterns of picocyanobacterial communities in winter and summer in the Chesapeake Bay. Appl Environ Microbiol 76:2955–2960. doi:10.1128/AEM.02868-09 20228109 PMC2863441

[71] Bertos-Fortis M, Farnelid HM, Lindh MV, Casini M, Andersson A, Pinhassi J, Legrand C. 2016. Unscrambling cyanobacteria community dynamics related to environmental factors. Front Microbiol 7:625. doi:10.3389/fmicb.2016.00625 27242679 PMC4860504

[72] Laës-Huon A, Davy R, Thomas L, Devesa J, Hemery A, Waeles M, El Rakwe M, Riso R, Dulaquais G. 2022. Rapid and simple determination of iron-porphyrin-like complexes (Fe-Py) in estuarine and marine waters. Mar Chem 244:104139. doi:10.1016/j.marchem.2022.104139

[73] Rúa A, Liebezeit G, Grajales H, Palacio J. 2017. Analyzing sources to sedimentary organic carbon in the Gulf of Urabá, southern caribbean, using carbon stable isotopes. J S Am Earth Sci 78:134–140. doi:10.1016/j.jsames.2017.06.011

[74] Loh KC, Chua SS. 2002. Ortho pathway of benzoate degradation in Pseudomonas putida: induction of meta pathway at high substrate concentrations. Enzyme Microb Technol 30:620–626. doi:10.1016/S0141-0229(02)00016-9

[75] Silva BSO, Nobrega MS, Leomil L, Tschoeke DA, Garcia GD, Dias G, Thompson CC, Thompson FL. 2017. Draft genome sequence of Pseudoalteromonas sp. Strain PAB 2.2 Isolated from abrolhos bank (Brazil). Genome Announc 5:e00016-17. doi:10.1128/genomeA.00016-17 28280012 PMC5347232

[76] Koch H, Germscheid N, Freese HM, Noriega-Ortega B, Lücking D, Berger M, Qiu G, Marzinelli EM, Campbell AH, Steinberg PD, Overmann J, Dittmar T, Simon M, Wietz M. 2020. Genomic, metabolic and phenotypic variability shapes ecological differentiation and intraspecies interactions of Alteromonas macleodii. Sci Rep 10:809. doi:10.1038/s41598-020-57526-5 31964928 PMC6972757

[77] Montoya Jaramillo LJ, Toro Botero M, Gomez-Giraldo A. 2017. Study of Atrato river plume in a tropical estuary: effects of the wind and tidal regime on the Gulf of Uraba, Colombia. DYNA 84:367–375. doi:10.15446/dyna.v84n200.55040

[78] Harrison TD, Whitfield AK. 2006. Temperature and salinity as primary determinants influencing the biogeography of fishes in South African estuaries. Estuar Coast Shelf Sci 66:335–345. doi:10.1016/j.ecss.2005.09.010

[79] Vasconcelos RP, Henriques S, França S, Pasquaud S, Cardoso I, Laborde M, Cabral HN. 2015. Global patterns and predictors of fish species richness in estuaries. J Anim Ecol 84:1331–1341. doi:10.1111/1365-2656.12372 25788236

[80] Liu B, Zheng Y, Wang X, Qi L, Zhou J, An Z, Wu L, Chen F, Lin Z, Yin G, Dong H, Li X, Liang X, Han P, Liu M, Hou L. 2024. Active dark carbon fixation evidenced by 14C isotope assimilation and metagenomic data across the estuarine-coastal continuum. Sci Total Environ 914:169833. doi:10.1016/j.scitotenv.2023.169833 38190922

[81] Callieri C, Cronberg G, Stockner JG. 2012. Freshwater picocyanobacteria: single cells, microcolonies and colonial forms, p 229–269. In Whitton B (ed), Ecology of cyanobacteria II. Springer, Dordrecht.

[82] Newton RJ, McLellan SL. 2015. A unique assemblage of cosmopolitan freshwater bacteria and higher community diversity differentiate an urbanized estuary from oligotrophic Lake Michigan. Front Microbiol 6:1028. doi:10.3389/fmicb.2015.01028 26483766 PMC4586452

[83] Hahn MW, Lang E, Brandt U, Lünsdorf H, Wu QL, Stackebrandt E. 2010. Polynucleobacter cosmopolitanus sp. nov., free-living planktonic bacteria inhabiting freshwater lakes and rivers. Int J Syst Evol Microbiol 60:166–173. doi:10.1099/ijs.0.010595-0 19648339 PMC2957076

[84] Ohore OE, Wei Y, Wang Y, Nwankwegu AS, Wang Z. 2022. Tracking the influence of antibiotics, antibiotic resistomes, and salinity gradient in modulating microbial community assemblage of surface water and the ecological consequences. Chemosphere 305:135428. doi:10.1016/j.chemosphere.2022.135428 35760129

[85] Zhao Z, Zhang K, Wu N, Li W, Xu W, Zhang Y, Niu Z. 2020. Estuarine sediments are key hotspots of intracellular and extracellular antibiotic resistance genes: a high-throughput analysis in Haihe Estuary in China. Environ Int 135:105385. doi:10.1016/j.envint.2019.105385 31855802

[86] Zhou L, Xu P, Gong J, Huang S, Chen W, Fu B, Zhao Z, Huang X. 2022. Metagenomic profiles of the resistome in subtropical estuaries: co-occurrence patterns, indicative genes, and driving factors. Sci Total Environ 810:152263. doi:10.1016/j.scitotenv.2021.152263 34896510

[87] Na G, Lu Z, Gao H, Zhang L, Li Q, Li R, Yang F, Huo C, Yao Z. 2018. The effect of environmental factors and migration dynamics on the prevalence of antibiotic-resistant Escherichia coli in estuary environments. Sci Rep 8:1663. doi:10.1038/s41598-018-20077-x 29374235 PMC5786026

[88] Abdelhamid AG, Yousef AE. 2024. Untargeted metabolomics unveiled the role of butanoate metabolism in the development of Pseudomonas aeruginosa hypoxic biofilm. Front Cell Infect Microbiol 14:1346813. doi:10.3389/fcimb.2024.1346813 38435305 PMC10904581

[89] Ruiz-Fernández P, Ramírez-Flandes S, Rodríguez-León E, Ulloa O. 2020. Autotrophic carbon fixation pathways along the redox gradient in oxygen-depleted oceanic waters. Environ Microbiol Rep 12:334–341. doi:10.1111/1758-2229.12837 32202395 PMC7318340

[90] Kothe E. 2011. Microbial degradation, p 596–599. In Reitner J, Thiel V (ed), Encyclopedia of geobiology. Encyclopedia of earth sciences series. Springer, Dordrecht.

[91] Langenheder S, Bulling MT, Solan M, Prosser JI. 2016. Bacterial biodiversity-ecosystem functioning relations are modified by environmental complexity. PLoS ONE 5:e10834. doi:10.1371/journal.pone.0010834 PMC2877076 20520808

[92] Delgado‐Baquerizo M, Giaramida L, Reich PB, Khachane AN, Hamonts K, Edwards C, Lawton LA, Singh BK. 2016. Lack of functional redundancy in the relationship between microbial diversity and ecosystem functioning. J Ecol 104:936–946. doi:10.1111/1365-2745.12585

[93] Louca S, Polz MF, Mazel F, Albright MBN, Huber JA, O’Connor MI, Ackermann M, Hahn AS, Srivastava DS, Crowe SA, Doebeli M, Parfrey LW. 2018. Function and functional redundancy in microbial systems. Nat Ecol Evol 2:936–943. doi:10.1038/s41559-018-0519-1 29662222

[94] Royalty TM, Steen AD. 2021. Functional redundancy in Ocean microbiomes controls trait stability. bioRxiv. doi:10.1101/2021.06.18.448980

[95] Cheng WH, Hsieh CH, Chang CW, Shiah FK, Miki T. 2022. New index of functional specificity to predict the redundancy of ecosystem functions in microbial communities. FEMS Microbiol Ecol 98:fiac058. doi:10.1093/femsec/fiac058 35568503

[96] Galand PE, Pereira O, Hochart C, Auguet JC, Debroas D. 2018. A strong link between marine microbial community composition and function challenges the idea of functional redundancy. ISME J 12:2470–2478. doi:10.1038/s41396-018-0158-1 29925880 PMC6155072

[97] Goswami M, Bhattacharyya P, Mukherjee I, Tribedi P. 2017. Functional diversity: an important measure of ecosystem functioning. Adv Microbiol 07:82–93. doi:10.4236/aim.2017.71007

[98] Muscarella R, Uriarte M. 2016. Do community-weighted mean functional traits reflect optimal strategies? Proc R Soc B 283:20152434. doi:10.1098/rspb.2015.2434 PMC4822452 27030412

[99] Castañeda LE, Barbosa O. 2017. Metagenomic analysis exploring taxonomic and functional diversity of soil microbial communities in chilean vineyards and surrounding native forests. PeerJ 5:e3098. doi:10.7717/peerj.3098 28382231 PMC5376117

[100] Song W, Liu J, Qin W, Huang J, Yu X, Xu M, Stahl D, Jiao N, Zhou J, Tu Q. 2022. Functional traits resolve mechanisms governing the assembly and distribution of nitrogen-cycling microbial communities in the global Ocean. mBio 13:e0383221. doi:10.1128/mbio.03832-21 35285696 PMC9040759

[101] Hastenrath S. 1990. Diagnostics and prediction of anomalous River discharge in northern South America. J Climate 3:1080–1096. doi:10.1175/1520-0442(1990)003<1080:DAPOAR>2.0.CO;2

[102] Dickson AG. 1994. Determination of dissolved oxygen in sea water by Winkler titration, p 1–14. In WOCE hydrographic program, operations and methods manual. Woods Hole Oceanographic Institute, Woods Hole, Mass.

[103] Callahan BJ, McMurdie PJ, Rosen MJ, Han AW, Johnson AJA, Holmes SP. 2016. DADA2: high-resolution sample inference from Illumina amplicon data. Nat Methods 13:581–583. doi:10.1038/nmeth.3869 27214047 PMC4927377

[104] Edgar RC, Flyvbjerg H. 2015. Error filtering, pair assembly and error correction for next-generation sequencing reads. Bioinformatics 31:3476–3482. doi:10.1093/bioinformatics/btv401 26139637

[105] Wang Q, Garrity GM, Tiedje JM, Cole JR. 2007. Naive bayesian classifier for rapid assignment of rRNA sequences into the new bacterial taxonomy. Appl Environ Microbiol 73:5261–5267. doi:10.1128/AEM.00062-07 17586664 PMC1950982

[106] McMurdie PJ, Holmes S. 2013. Phyloseq: an R package for reproducible interactive analysis and graphics of microbiome census data. PLoS One 8:e61217. doi:10.1371/journal.pone.0061217 23630581 PMC3632530

[107] Nagpal S, Haque MM, Singh R, Mande SS. 2018. iVikodak-A platform and standard workflow for inferring, analyzing, comparing, and visualizing the functional potential of microbial communities. Front Microbiol 9:3336. doi:10.3389/fmicb.2018.03336 30692979 PMC6339920

[108] Cornwell WK, Schwilk DW, Ackerly DD. 2006. A trait-based test for habitat filtering: convex hull volume. Ecology 87:1465–1471. doi:10.1890/0012-9658(2006)87[1465:ATTFHF]2.0.CO;2 16869422

[109] Villéger S, Mason NWH, Mouillot D. 2008. New multidimensional functional diversity indices for a multifaceted framework in functional ecology. Ecology 89:2290–2301. doi:10.1890/07-1206.1 18724739

[110] Magneville C, Loiseau N, Albouy C, Casajus N, Claverie T, Escalas A, Leprieur F, Maire E, Mouillot D, Villéger S. 2022. mFD: an R package to compute and illustrate the multiple facets of functional diversity. Ecography 2022:e05904. doi:10.1111/ecog.05904

[111] Weiher E, Clarke GDP, Keddy PA. 1998. Community assembly rules, morphological dispersion, and the coexistence of plant species. Oikos 81:309. doi:10.2307/3547051

[112] Podani J, Schmera D. 2006. On dendrogram‐based measures of functional diversity. Oikos 115:179–185. doi:10.1111/j.2006.0030-1299.15048.x

[113] Machado KB, Teresa FB, Nabout JC. 2017. Assessing the spatial variation of functional diversity estimates based on dendrograms in phytoplankton communities. Acta Bot Bras 31:571–582. doi:10.1590/0102-33062017abb0018

[114] Kang S, Ma WJ, Li FY, Zhang Q, Niu JM, Ding Y, Han F, Sun X. 2015. Functional redundancy instead of species redundancy determines community stability in a typical steppe of inner mongolia. PLoS One 10:e0145605. doi:10.1371/journal.pone.0145605 26699133 PMC4689422

[115] Ricotta C, de Bello F, Moretti M, Caccianiga M, Cerabolini BEL, Pavoine S. 2016. Measuring the functional redundancy of biological communities: a quantitative guide. Methods Ecol Evol 7:1386–1395. doi:10.1111/2041-210X.12604

[116] Herrera DL, Navarrete SA, Labra FA, Castillo SP, Opazo Mella L. 2023. Functional biogeography of coastal marine invertebrates along the south‐eastern Pacific coast reveals latitudinally divergent drivers of taxonomic versus functional diversity. Ecography 2023. doi:10.1111/ecog.06476

[117] Oksanen J, Blanchet FG, Kindt R, Legendre P, Minchin PR, O’Hara RB, Simpson GL, Solymos P, Stevens MHH, Wagner H. 2015. R package “vegan”: community ecology package. R package version 2.3-0. Available from: http://CRAN.Rproject.org/package=vegan

[118] R Core Team. 2023. R: A language and environment for statistical computing (version 4.3.0). R foundation for statistical computing. Available from: https://www.r-project.org/

[119] Laliberté E, Legendre P. 2010. A distance‐based framework for measuring functional diversity from multiple traits. Ecology 91:299–305. doi:10.1890/08-2244.1 20380219

[120] Laliberté E, Legendre P, Shipley B. 2022. Measuring functional diversity (FD) from multiple traits, and other tools for functional ecology. R Package version 1.0-12.1. Available from: https://CRAN.R-project.org/package=FD 10.1890/08-2244.1 20380219

[121] Anderson MJ. 2001. A new method for non‐parametric multivariate analysis of variance. Austral Ecol 26:32–46. doi:10.1111/j.1442-9993.2001.01070.pp.x

[122] Legendre P, Gallagher ED. 2001. Ecologically meaningful transformations for ordination of species data. Oecologia 129:271–280. doi:10.1007/s004420100716 28547606

[123] Legendre P, Oksanen J, ter Braak CJF. 2011. Testing the significance of canonical axes in redundancy analysis. Methods Ecol Evol 2:269–277. doi:10.1111/j.2041-210X.2010.00078.x

[124] Dray S, Choler P, Dolédec S, Peres-Neto PR, Thuiller W, Pavoine S, ter Braak CJF. 2014. Combining the fourth-corner and the RLQ methods for assessing trait responses to environmental variation. Ecology 95:14–21. doi:10.1890/13-0196.1 24649641

[125] Dray S, Dufour AB. 2007. The ade4 package: implementing the duality diagram for ecologists. J Stat Soft 22:1–20. doi:10.18637/jss.v022.i04

