# Supplementary material for: Estimating taxonomic and functional structure along a tropical estuary: linking metabolic traits and aspects of ecosystem functioning
Source: Microbiol Spectr. 2024 Aug 20;12(10):e03886-23. doi: 10.1128/spectrum.03886-23 (PMC11448197; doi:10.1128/spectrum.03886-23)
Supplement: Supplemental material — Tables S1 to S6; Fig. S1 to S5. [file spectrum.03886-23-s0001.docx]

Supplementary Material

Estimating taxonomic and functional structure along a tropical estuary: Linking metabolic traits and aspects of ecosystem functioning

Héctor A. Levipan*, L. Felipe Opazo, Sara Arenas-Uribe, Hernán Wicki, Francisca Marchant, Lennin Florez-Leiva, and Ruben Avendaño-Herrera

*** Correspondence:** [hector.levipan@upla.cl](mailto:hector.levipan@upla.cl)

# Supplementary tables: 6

**Table S1. Homogeneous groups based on the Tukey HSD test for genera richness and functional facets of the prokaryote communities.** Averages that do not share a letter are significantly different at a significance level of 0.0001 (= ****).

| Variable | Station | Depth (m) | Average value | a | b | c | d | e | f |
| --- | --- | --- | --- | --- | --- | --- | --- | --- | --- |
| Genera richness | 2 | 11 | 105 |  | **** |  |  |  |  |
|  | 7 | 0.5 | 102 | **** | **** |  |  |  |  |
|  | 7 | 11 | 97 | **** | **** |  |  |  |  |
|  | 13 | 0.5 | 92 | **** | **** |  | **** |  |  |
|  | 13 | 11 | 88 | **** | **** | **** | **** |  |  |
|  | 15 | 11 | 85 | **** | **** | **** | **** |  |  |
|  | 4 | 11 | 73 | **** | **** | **** | **** | **** |  |
|  | 9 | 11 | 71 | **** |  | **** | **** | **** |  |
|  | 2 | 0.5 | 64 |  |  | **** | **** | **** |  |
|  | 4 | 0.5 | 55 |  |  | **** |  | **** | **** |
|  | 15 | 0.5 | 49 |  |  |  |  | **** | **** |
|  | 9 | 0.5 | 31 |  |  |  |  |  | **** |
|  |  |  |  |  |  |  |  |  |  |
| Functional richness | 9 | 0.5 | 0.110 | **** |  |  |  |  |  |
|  | 2 | 0.5 | 0.112 | **** |  |  |  |  |  |
|  | 4 | 0.5 | 0.183 | **** | **** |  |  |  |  |
|  | 7 | 11 | 0.235 | **** | **** |  |  |  |  |
|  | 15 | 0.5 | 0.254 | **** | **** |  |  |  |  |
|  | 13 | 11 | 0.286 | **** | **** |  |  |  |  |
|  | 9 | 11 | 0.324 | **** | **** |  |  |  |  |
|  | 15 | 11 | 0.416 | **** | **** |  |  |  |  |
|  | 13 | 0.5 | 0.438 | **** | **** |  |  |  |  |
|  | 4 | 11 | 0.471 | **** | **** |  |  |  |  |
|  | 2 | 11 | 0.485 | **** | **** |  |  |  |  |
|  | 7 | 0.5 | 0.520 |  | **** |  |  |  |  |
|  |  |  |  |  |  |  |  |  |  |
| Functional Mean Pairwise Distance (FMPD) | 7 | 11 | 0.188 | **** |  |  |  |  |  |
|  | 9 | 11 | 0.192 | **** |  |  |  |  |  |
|  | 7 | 0.5 | 0.211 | **** | **** |  |  |  |  |
|  | 13 | 11 | 0.214 | **** | **** |  |  |  |  |
|  | 2 | 11 | 0.224 | **** | **** | **** |  |  |  |
|  | 2 | 0.5 | 0.226 | **** | **** | **** |  |  |  |
|  | 4 | 0.5 | 0.227 | **** | **** | **** |  |  |  |
|  | 4 | 11 | 0.241 |  | **** | **** | **** |  |  |
|  | 15 | 11 | 0.256 |  | **** | **** | **** |  |  |
|  | 13 | 0.5 | 0.260 |  |  | **** | **** |  |  |
|  | 9 | 0.5 | 0.267 |  |  | **** | **** |  |  |
|  | 15 | 0.5 | 0.287 |  |  |  | **** |  |  |
|  |  |  |  |  |  |  |  |  |  |
| Functional redundancy | 15 | 0.5 – 11 | 0.204 | **** |  |  |  |  |  |
|  | 13 | 0.5 – 11 | 0.223 | **** |  |  |  |  |  |
|  | 9 | 0.5 – 11 | 0.243 | **** |  |  |  |  |  |
|  | 7 | 0.5 – 11 | 0.416 | **** | **** |  |  |  |  |
|  | 4 | 0.5 – 11 | 0.537 | **** | **** |  |  |  |  |
|  | 2 | 0.5 – 11 | 0.743 |  | **** |  |  |  |  |
|  |  |  |  |  |  |  |  |  |  |
| Functional evenness | 13 | 0.5 | 0.312 |  | **** |  |  |  |  |
|  | 13 | 11 | 0.334 |  | **** | **** |  |  |  |
|  | 7 | 0.5 | 0.420 | **** | **** | **** |  |  |  |
|  | 15 | 11 | 0.424 | **** | **** | **** |  |  |  |
|  | 7 | 11 | 0.425 | **** | **** | **** |  |  |  |
|  | 9 | 11 | 0.425 | **** | **** | **** |  |  |  |
|  | 2 | 0.5 | 0.445 | **** | **** | **** |  |  |  |
|  | 4 | 0.5 | 0.466 | **** | **** | **** |  |  |  |
|  | 9 | 0.5 | 0.482 | **** |  | **** |  |  |  |
|  | 2 | 11 | 0.505 | **** |  |  |  |  |  |
|  | 4 | 11 | 0.509 | **** |  |  |  |  |  |
|  | 15 | 0.5 | 0.546 | **** |  |  |  |  |  |

**Table S2. SIMPER results for prokaryote community composition of sample clusters along the transect.** M_group1_ = mean abundance by genus at group 1 (St2 to St7), M_group2_ = mean abundance by genus at group 2 (St9 to St15). AD = average dissimilarity of typifying genera in each sample, contribution (C%) = percentage contribution to dissimilarity for each genus, cumulative (Cu%) = cumulative percentage of dissimilarity. The overall average dissimilarity was equal to 81%.

| **Genera** | **M_group1_** | **M_group2_** | **AD** | **C%** | **Cu%** |
| --- | --- | --- | --- | --- | --- |
| *Alteromonas* | 0.06 | 0 | 10.93 | 14.72 | 14.72 |
| *Synechococcus* | 0.05 | 0.07 | 10.72 | 14.44 | 29.16 |
| *Cyanobium* | 0.01 | 0.04 | 6.63 | 8.92 | 38.08 |
| *Prochlorococcus* | 0.01 | 0.03 | 4.40 | 5.93 | 44.01 |
| *Pseudomonas* | 0.02 | 0 | 3.54 | 4.77 | 48.78 |
| *Pseudoalteromonas* | 0.02 | 0 | 3.35 | 4.51 | 53.29 |
| *Polynucleobacter* | 0 | 0.01 | 2.48 | 3.35 | 56.64 |
| *Fluviicola* | 0 | 0.01 | 2.48 | 3.34 | 59.98 |
| *Roseivivax* | 0 | 0.01 | 2.28 | 3.07 | 63.04 |
| *Magnetospira* | 0.01 | 0 | 1.5 | 2.02 | 65.06 |
| *Saprospira* | 0.01 | 0 | 1.49 | 2 | 67.06 |
| *Aureispira* | 0.01 | 0 | 1.45 | 1.95 | 69.02 |
| *Limnohabitans* | 0 | 0.01 | 1.42 | 1.91 | 70.93 |
| *Methylomonas* | 0 | 0.01 | 1.17 | 1.58 | 72.51 |
| *Nitrospina* | 0.01 | 0 | 1.04 | 1.39 | 73.9 |
| *Comamonas* | 0 | 0 | 0.91 | 1.23 | 75.13 |
| *Aestuariibacter* | 0 | 0 | 0.91 | 1.22 | 76.35 |
| *Acinetobacter* | 0 | 0 | 0.87 | 1.17 | 77.52 |
| *Litorimicrobium* | 0 | 0 | 0.81 | 1.09 | 78.61 |
| *Alcanivorax* | 0 | 0 | 0.79 | 1.06 | 79.67 |
| *Grimontia* | 0 | 0 | 0.76 | 1.02 | 80.69 |
| *Idiomarina* | 0 | 0 | 0.71 | 0.96 | 81.65 |
| *Ketogulonicigenium* | 0 | 0 | 0.70 | 0.95 | 82.6 |
| *Microbulbifer* | 0 | 0 | 0.66 | 0.89 | 83.49 |
| *Balneola* | 0 | 0 | 0.63 | 0.85 | 84.34 |
| *Marinobacterium* | 0 | 0 | 0.6 | 0.81 | 85.15 |
| *Crocinitomix* | 0 | 0 | 0.55 | 0.74 | 85.89 |
| *Limnobacter* | 0 | 0 | 0.52 | 0.70 | 86.59 |
| *Candidatus Puniceispirillum* | 0 | 0 | 0.51 | 0.69 | 87.29 |
| *Ramlibacter* | 0 | 0 | 0.39 | 0.53 | 87.82 |
| *Rheinheimera* | 0 | 0 | 0.38 | 0.52 | 88.33 |
| *Coxiella* | 0 | 0 | 0.37 | 0.50 | 88.84 |
| *Sediminibacterium* | 0 | 0 | 0.34 | 0.46 | 89.30 |
| *Ottowia* | 0 | 0 | 0.31 | 0.42 | 89.71 |
| *Winogradskyella* | 0 | 0 | 0.31 | 0.42 | 90.13 |
| *Tenacibaculum* | 0 | 0 | 0.28 | 0.38 | 90.52 |
| *Marinomonas* | 0 | 0 | 0.26 | 0.34 | 90.86 |
| *Flavobacterium* | 0 | 0 | 0.24 | 0.32 | 91.18 |
| *Lautropia* | 0 | 0 | 0.20 | 0.27 | 91.45 |
| *Oceanobacter* | 0 | 0 | 0.19 | 0.26 | 91.71 |
| *Arcobacter* | 0 | 0 | 0.19 | 0.26 | 91.97 |
| *Bdellovibrio* | 0 | 0 | 0.18 | 0.24 | 92.21 |
| *Salinimonas* | 0 | 0 | 0.17 | 0.23 | 92.43 |
| *Vibrio* | 0 | 0 | 0.16 | 0.22 | 92.65 |
| *Chthoniobacter* | 0 | 0 | 0.16 | 0.22 | 92.87 |
| *Terrimonas* | 0 | 0 | 0.15 | 0.20 | 93.07 |
| *Curvibacter* | 0 | 0 | 0.14 | 0.19 | 93.26 |
| *Noviherbaspirillum* | 0 | 0 | 0.14 | 0.18 | 93.45 |
| *Halomonas* | 0 | 0 | 0.13 | 0.18 | 93.62 |
| *Lutibacter* | 0 | 0 | 0.13 | 0.18 | 93.8 |
| *Tropicimonas* | 0 | 0 | 0.12 | 0.16 | 93.96 |
| *Candidatus Aquiluna* | 0 | 0 | 0.12 | 0.16 | 94.11 |
| *Candidatus Endoecteinascidia* | 0 | 0 | 0.12 | 0.16 | 94.27 |
| *Salinimicrobium* | 0 | 0 | 0.11 | 0.15 | 94.42 |
| *Bryobacter* | 0 | 0 | 0.11 | 0.14 | 94.56 |
| *Pseudarcicella* | 0 | 0 | 0.10 | 0.14 | 94.7 |
| *Alishewanella* | 0 | 0 | 0.10 | 0.14 | 94.83 |
| *Thalassolituus* | 0 | 0 | 0.10 | 0.13 | 94.96 |
| *Occidentia* | 0 | 0 | 0.09 | 0.13 | 95.09 |
| *Oleiphilus* | 0 | 0 | 0.09 | 0.12 | 95.21 |
| *Maricaulis* | 0 | 0 | 0.09 | 0.12 | 95.33 |
| *Bermanella* | 0 | 0 | 0.09 | 0.12 | 95.46 |
| *Methylomarinum* | 0 | 0 | 0.09 | 0.12 | 95.58 |
| *Erythrobacter* | 0 | 0 | 0.09 | 0.12 | 95.70 |
| *Novosphingobium* | 0 | 0 | 0.08 | 0.10 | 95.80 |
| *Bauldia* | 0 | 0 | 0.07 | 0.10 | 95.90 |
| *Weissella* | 0 | 0 | 0.07 | 0.10 | 96.00 |
| *Robiginitalea* | 0 | 0 | 0.07 | 0.10 | 96.09 |
| *Lewinella* | 0 | 0 | 0.07 | 0.09 | 96.19 |
| *Sphingomonas* | 0 | 0 | 0.06 | 0.09 | 96.27 |
| *Hydrogenophaga* | 0 | 0 | 0.06 | 0.08 | 96.35 |
| *Reyranella* | 0 | 0 | 0.06 | 0.08 | 96.43 |
| *Bacillus* | 0 | 0 | 0.06 | 0.08 | 96.51 |
| *Candidatus Solibacter* | 0 | 0 | 0.06 | 0.08 | 96.59 |
| *Staphylococcus* | 0 | 0 | 0.05 | 0.07 | 96.66 |
| *Truepera* | 0 | 0 | 0.05 | 0.07 | 96.73 |
| *Endozoicomonas* | 0 | 0 | 0.05 | 0.07 | 96.79 |
| *Algoriphagus* | 0 | 0 | 0.05 | 0.06 | 96.86 |
| *Algicola* | 0 | 0 | 0.05 | 0.06 | 96.92 |
| *Massilia* | 0 | 0 | 0.04 | 0.06 | 96.98 |
| *Marinobacter* | 0 | 0 | 0.04 | 0.06 | 97.04 |
| *Rhodobacter* | 0 | 0 | 0.04 | 0.06 | 97.10 |
| *Zunongwangia* | 0 | 0 | 0.04 | 0.06 | 97.15 |
| *Draconibacterium* | 0 | 0 | 0.04 | 0.06 | 97.21 |
| *Formosa* | 0 | 0 | 0.04 | 0.06 | 97.27 |
| *Nitrospira* | 0 | 0 | 0.04 | 0.06 | 97.33 |
| *Salinihabitans* | 0 | 0 | 0.04 | 0.06 | 97.38 |
| *Roseomonas* | 0 | 0 | 0.04 | 0.05 | 97.44 |
| *Verrucomicrobium* | 0 | 0 | 0.04 | 0.05 | 97.49 |
| *Coraliomargarita* | 0 | 0 | 0.04 | 0.05 | 97.54 |
| *Acholeplasma* | 0 | 0 | 0.04 | 0.05 | 97.59 |
| *Pontibacter* | 0 | 0 | 0.04 | 0.05 | 97.64 |
| *Mesonia* | 0 | 0 | 0.04 | 0.05 | 97.69 |
| *Salegentibacter* | 0 | 0 | 0.04 | 0.05 | 97.74 |
| *Lacibacter* | 0 | 0 | 0.03 | 0.05 | 97.79 |
| *Tranquillimonas* | 0 | 0 | 0.03 | 0.04 | 97.83 |
| *Haliscomenobacter* | 0 | 0 | 0.03 | 0.04 | 97.87 |
| *Neptunomonas* | 0 | 0 | 0.03 | 0.04 | 97.92 |
| *Rhodoferax* | 0 | 0 | 0.03 | 0.04 | 97.96 |
| *Opitutus* | 0 | 0 | 0.03 | 0.04 | 98.00 |
| *Sphingobium* | 0 | 0 | 0.03 | 0.04 | 98.04 |
| *Shewanella* | 0 | 0 | 0.03 | 0.04 | 98.09 |
| *Enterovibrio* | 0 | 0 | 0.03 | 0.04 | 98.13 |
| *Rhodovulum* | 0 | 0 | 0.03 | 0.04 | 98.17 |
| *Azospirillum* | 0 | 0 | 0.03 | 0.04 | 98.20 |
| *Empedobacter* | 0 | 0 | 0.03 | 0.04 | 98.24 |
| *Delftia* | 0 | 0 | 0.03 | 0.04 | 98.28 |
| *Filomicrobium* | 0 | 0 | 0.03 | 0.04 | 98.32 |
| *Streptococcus* | 0 | 0 | 0.03 | 0.04 | 98.35 |
| *Tropicibacter* | 0 | 0 | 0.03 | 0.04 | 98.39 |
| *Geobacter* | 0 | 0 | 0.03 | 0.04 | 98.43 |
| *Fangia* | 0 | 0 | 0.03 | 0.04 | 98.46 |
| *Anaeromyxobacter* | 0 | 0 | 0.03 | 0.04 | 98.50 |
| *Paracoccus* | 0 | 0 | 0.03 | 0.03 | 98.53 |
| *Marivita* | 0 | 0 | 0.03 | 0.03 | 98.56 |
| *Methylocystis* | 0 | 0 | 0.02 | 0.03 | 98.60 |
| *Brevundimonas* | 0 | 0 | 0.02 | 0.03 | 98.63 |
| *Sulfitobacter* | 0 | 0 | 0.02 | 0.03 | 98.66 |
| *Aquimarina* | 0 | 0 | 0.02 | 0.03 | 98.69 |
| *Bacteroides* | 0 | 0 | 0.02 | 0.03 | 98.72 |
| *Terasakiella* | 0 | 0 | 0.02 | 0.03 | 98.75 |
| *Methylocaldum* | 0 | 0 | 0.02 | 0.03 | 98.78 |
| *Gramella* | 0 | 0 | 0.02 | 0.03 | 98.81 |
| *Rhodopirellula* | 0 | 0 | 0.02 | 0.03 | 98.84 |
| *Saccharophagus* | 0 | 0 | 0.02 | 0.03 | 98.86 |
| *Roseobacter* | 0 | 0 | 0.02 | 0.03 | 98.89 |
| *Methylocella* | 0 | 0 | 0.02 | 0.03 | 98.92 |
| *Kordiimonas* | 0 | 0 | 0.02 | 0.03 | 98.94 |
| *Thalassococcus* | 0 | 0 | 0.02 | 0.02 | 98.97 |
| *Altererythrobacter* | 0 | 0 | 0.02 | 0.02 | 98.99 |
| *Psychrobacter* | 0 | 0 | 0.02 | 0.02 | 99.02 |
| *Sphingobacterium* | 0 | 0 | 0.02 | 0.02 | 99.04 |
| *Hyphomicrobium* | 0 | 0 | 0.02 | 0.02 | 99.06 |
| *Geothrix* | 0 | 0 | 0.02 | 0.02 | 99.09 |
| *Caulobacter* | 0 | 0 | 0.02 | 0.02 | 99.11 |
| *Ferriphaselus* | 0 | 0 | 0.02 | 0.02 | 99.13 |
| *Beggiatoa* | 0 | 0 | 0.02 | 0.02 | 99.15 |
| *Neptuniibacter* | 0 | 0 | 0.01 | 0.02 | 99.17 |
| *Salinisphaera* | 0 | 0 | 0.01 | 0.02 | 99.19 |
| *Ideonella* | 0 | 0 | 0.01 | 0.02 | 99.21 |
| *Sandaracinus* | 0 | 0 | 0.01 | 0.02 | 99.23 |
| *Erysipelotrichaceae* | 0 | 0 | 0.01 | 0.02 | 99.25 |
| *Reinekea* | 0 | 0 | 0.01 | 0.02 | 99.26 |
| *Croceicoccus* | 0 | 0 | 0.01 | 0.02 | 99.28 |
| *Polaromonas* | 0 | 0 | 0.01 | 0.02 | 99.30 |
| *Aeromonas* | 0 | 0 | 0.01 | 0.02 | 99.31 |
| *Sphaerotilus* | 0 | 0 | 0.01 | 0.02 | 99.33 |
| *Sulfuricurvum* | 0 | 0 | 0.01 | 0.02 | 99.34 |
| *Cetobacterium* | 0 | 0 | 0.01 | 0.02 | 99.36 |
| *Microscilla* | 0 | 0 | 0.01 | 0.02 | 99.38 |
| *Actibacterium* | 0 | 0 | 0.01 | 0.01 | 99.39 |
| *Thalassotalea* | 0 | 0 | 0.01 | 0.01 | 99.40 |
| *Magnetospirillum* | 0 | 0 | 0.01 | 0.01 | 99.42 |
| *Propionigenium* | 0 | 0 | 0.01 | 0.01 | 99.43 |
| *Pedobacter* | 0 | 0 | 0.01 | 0.01 | 99.44 |
| *Phenylobacterium* | 0 | 0 | 0.01 | 0.01 | 99.46 |
| *Henriciella* | 0 | 0 | 0.01 | 0.01 | 99.47 |
| *Lysinibacillus* | 0 | 0 | 0.01 | 0.01 | 99.48 |
| *Leptospira* | 0 | 0 | 0.01 | 0.01 | 99.49 |
| *Sideroxydans* | 0 | 0 | 0.01 | 0.01 | 99.50 |
| *Ulvibacter* | 0 | 0 | 0.01 | 0.01 | 99.52 |
| *Pseudoruegeria* | 0 | 0 | 0.01 | 0.01 | 99.53 |
| *Cellvibrio* | 0 | 0 | 0.01 | 0.01 | 99.54 |
| *Chryseobacterium* | 0 | 0 | 0.01 | 0.01 | 99.55 |
| *Niveispirillum* | 0 | 0 | 0.01 | 0.01 | 99.56 |
| *Candidatus Symbiobacter* | 0 | 0 | 0.01 | 0.01 | 99.57 |
| *Thalassospira* | 0 | 0 | 0.01 | 0.01 | 99.58 |
| *Thiothrix* | 0 | 0 | 0.01 | 0.01 | 99.59 |
| *Aquabacterium* | 0 | 0 | 0.01 | 0.01 | 99.60 |
| *Rubrivivax* | 0 | 0 | 0.01 | 0.01 | 99.61 |
| *Legionella* | 0 | 0 | 0.01 | 0.01 | 99.62 |
| *Stenotrophomonas* | 0 | 0 | 0.01 | 0.01 | 99.63 |
| *Blastopirellula* | 0 | 0 | 0.01 | 0.01 | 99.64 |
| *Sulfurovum* | 0 | 0 | 0.01 | 0.01 | 99.64 |
| *Zoogloea* | 0 | 0 | 0.01 | 0.01 | 99.65 |
| *Sulfurospirillum* | 0 | 0 | 0.01 | 0.01 | 99.66 |
| *Synechocystis* | 0 | 0 | 0.01 | 0.01 | 99.67 |
| *Chromohalobacter* | 0 | 0 | 0.01 | 0.01 | 99.68 |
| *Leisingera* | 0 | 0 | 0.01 | 0.01 | 99.69 |
| *Methyloglobulus* | 0 | 0 | 0.01 | 0.01 | 99.69 |
| *Thiovulum* | 0 | 0 | 0.01 | 0.01 | 99.70 |
| *Exiguobacterium* | 0 | 0 | 0.01 | 0.01 | 99.71 |
| *Epibacterium* | 0 | 0 | 0.01 | 0.01 | 99.72 |
| *Rhodoluna* | 0 | 0 | 0.01 | 0.01 | 99.72 |
| *Domibacillus* | 0 | 0 | 0.01 | 0.01 | 99.73 |
| *Desulfopila* | 0 | 0 | 0.01 | 0.01 | 99.74 |
| *Oceanospirillum* | 0 | 0 | 0.01 | 0.01 | 99.74 |
| *Sulfurimonas* | 0 | 0 | 0 | 0.01 | 99.75 |
| *Alkanindiges* | 0 | 0 | 0 | 0.01 | 99.76 |
| *Simiduia* | 0 | 0 | 0 | 0.01 | 99.76 |
| *Catenovulum* | 0 | 0 | 0 | 0.01 | 99.77 |
| *Candidatus Amoebophilus* | 0 | 0 | 0 | 0.01 | 99.78 |
| *Emticicia* | 0 | 0 | 0 | 0.01 | 99.78 |
| *Cloacibacterium* | 0 | 0 | 0 | 0.01 | 99.79 |
| *Paludibacter* | 0 | 0 | 0 | 0.01 | 99.79 |
| *Flectobacillus* | 0 | 0 | 0 | 0.01 | 99.80 |
| *Oceanicaulis* | 0 | 0 | 0 | 0.01 | 99.80 |
| *Bacteriovorax* | 0 | 0 | 0 | 0 | 99.81 |
| *Bradyrhizobium* | 0 | 0 | 0 | 0 | 99.81 |
| *Gemella* | 0 | 0 | 0 | 0 | 99.82 |
| *Neochlamydia* | 0 | 0 | 0 | 0 | 99.82 |
| *Paenibacillus* | 0 | 0 | 0 | 0 | 99.83 |
| *Pseudorhodobacter* | 0 | 0 | 0 | 0 | 99.83 |
| *Spirochaeta* | 0 | 0 | 0 | 0 | 99.83 |
| *Cobetia* | 0 | 0 | 0 | 0 | 99.84 |
| *Marinovum* | 0 | 0 | 0 | 0 | 99.84 |
| *Anaerococcus* | 0 | 0 | 0 | 0 | 99.85 |
| *Gallaecimonas* | 0 | 0 | 0 | 0 | 99.85 |
| *Simkania* | 0 | 0 | 0 | 0 | 99.85 |
| *Escherichia / Shigella* | 0 | 0 | 0 | 0 | 99.86 |
| *Rhodocista* | 0 | 0 | 0 | 0 | 99.86 |
| *Desulfotignum* | 0 | 0 | 0 | 0 | 99.87 |
| *Herbaspirillum* | 0 | 0 | 0 | 0 | 99.87 |
| *Uliginosibacterium* | 0 | 0 | 0 | 0 | 99.87 |
| *Haliangium* | 0 | 0 | 0 | 0 | 99.88 |
| *Jeotgalibacillus* | 0 | 0 | 0 | 0 | 99.88 |
| *Marmoricola* | 0 | 0 | 0 | 0 | 99.88 |
| *Chlorobaculum* | 0 | 0 | 0 | 0 | 99.89 |
| *Perlucidibaca* | 0 | 0 | 0 | 0 | 99.89 |
| *Spongiibacter* | 0 | 0 | 0 | 0 | 99.89 |
| *Bergeyella* | 0 | 0 | 0 | 0 | 99.90 |
| *Desulfomonile* | 0 | 0 | 0 | 0 | 99.90 |
| *Syntrophus* | 0 | 0 | 0 | 0 | 99.90 |
| *Amphritea* | 0 | 0 | 0 | 0 | 99.91 |
| *Desulfovibrio* | 0 | 0 | 0 | 0 | 99.91 |
| *Leptothrix* | 0 | 0 | 0 | 0 | 99.91 |
| *Xanthomonas* | 0 | 0 | 0 | 0 | 99.91 |
| *Alistipes* | 0 | 0 | 0 | 0 | 99.92 |
| *Hyphomonas* | 0 | 0 | 0 | 0 | 99.92 |
| *Phycisphaera* | 0 | 0 | 0 | 0 | 99.92 |
| *Prevotellaceae* | 0 | 0 | 0 | 0 | 99.92 |
| *Celeribacter* | 0 | 0 | 0 | 0 | 99.93 |
| *Flammeovirga* | 0 | 0 | 0 | 0 | 99.93 |
| *Mucilaginibacter* | 0 | 0 | 0 | 0 | 99.93 |
| *Rhizobacter* | 0 | 0 | 0 | 0 | 99.93 |
| *Candidatus Paracaedibacter* | 0 | 0 | 0 | 0 | 99.94 |
| *Desulfobulbus* | 0 | 0 | 0 | 0 | 99.94 |
| *Lactobacillus* | 0 | 0 | 0 | 0 | 99.94 |
| *Teredinibacter* | 0 | 0 | 0 | 0 | 99.94 |
| *Aerococcus* | 0 | 0 | 0 | 0 | 99.95 |
| *Fusobacterium* | 0 | 0 | 0 | 0 | 99.95 |
| *Neisseria* | 0 | 0 | 0 | 0 | 99.95 |
| *Phaeospirillum* | 0 | 0 | 0 | 0 | 99.95 |
| *Solitalea* | 0 | 0 | 0 | 0 | 99.95 |
| *Achromobacter* | 0 | 0 | 0 | 0 | 99.96 |
| *Asticcacaulis* | 0 | 0 | 0 | 0 | 99.96 |
| *Cytophaga* | 0 | 0 | 0 | 0 | 99.96 |
| *Hirschia* | 0 | 0 | 0 | 0 | 99.96 |
| *Turneriella* | 0 | 0 | 0 | 0 | 99.96 |
| *Alloprevotella* | 0 | 0 | 0 | 0 | 99.97 |
| *Campylobacter* | 0 | 0 | 0 | 0 | 99.97 |
| *Epulopiscium* | 0 | 0 | 0 | 0 | 99.97 |
| *Luteimonas* | 0 | 0 | 0 | 0 | 99.97 |
| *Pirellula* | 0 | 0 | 0 | 0 | 99.97 |
| *Sedimenticola* | 0 | 0 | 0 | 0 | 99.97 |
| *Xylophilus* | 0 | 0 | 0 | 0 | 99.97 |
| *Labrenzia* | 0 | 0 | 0 | 0 | 99.98 |
| *Marinococcus* | 0 | 0 | 0 | 0 | 99.98 |
| *Methylophaga* | 0 | 0 | 0 | 0 | 99.98 |
| *Methylotenera* | 0 | 0 | 0 | 0 | 99.98 |
| *Mycobacterium* | 0 | 0 | 0 | 0 | 99.98 |
| *Polycyclovorans* | 0 | 0 | 0 | 0 | 99.98 |
| *Prevotella* | 0 | 0 | 0 | 0 | 99.98 |
| *Thalassobaculum* | 0 | 0 | 0 | 0 | 99.98 |
| *Candidatus Nitrosopumilus* | 0 | 0 | 0 | 0 | 99.99 |
| *Corynebacterium* | 0 | 0 | 0 | 0 | 99.99 |
| *Dysgonomonas* | 0 | 0 | 0 | 0 | 99.99 |
| *Kiloniella* | 0 | 0 | 0 | 0 | 99.99 |
| *Kordia* | 0 | 0 | 0 | 0 | 99.99 |
| *Methylomicrobium* | 0 | 0 | 0 | 0 | 99.99 |
| *Nannocystis* | 0 | 0 | 0 | 0 | 99.99 |
| *Nevskia* | 0 | 0 | 0 | 0 | 99.99 |
| *Simplicispira* | 0 | 0 | 0 | 0 | 99.99 |
| *Afipia* | 0 | 0 | 0 | 0 | 100 |
| *Candidatus Odyssella* | 0 | 0 | 0 | 0 | 100 |
| *Cellulosimicrobium* | 0 | 0 | 0 | 0 | 100 |
| *Desulfosarcina* | 0 | 0 | 0 | 0 | 100 |
| *Francisella* | 0 | 0 | 0 | 0 | 100 |
| *Fulvivirga* | 0 | 0 | 0 | 0 | 100 |
| *Leeuwenhoekiella* | 0 | 0 | 0 | 0 | 100 |
| *Lysobacter* | 0 | 0 | 0 | 0 | 100 |
| *Magnetococcus* | 0 | 0 | 0 | 0 | 100 |
| *Peptoniphilus* | 0 | 0 | 0 | 0 | 100 |
| *Selenomonas* | 0 | 0 | 0 | 0 | 100 |
| *Succinivibrio* | 0 | 0 | 0 | 0 | 100 |

**Table S3. SIMPER results for prokaryote community composition of sample clusters over depth.** M_B_ = mean abundance by genus at bottom aphotic samples (11 m depth), M_S_ = mean abundance by genus at surface photic samples (0.5 m depth). AD = Average dissimilarity of typifying genera in each sample. Contribution (C%) = percentage contribution to dissimilarity for each genus. Cumulative (Cu%) = cumulative percentage of dissimilarity. The overall average dissimilarity was equal to 74%.

| **Genera** | **M_B_** | **M_S_** | **AD** | **C%** | **Cu%** |
| --- | --- | --- | --- | --- | --- |
| *Synechococcus* | 0.02 | 0.10 | 19.23 | 26.02 | 26.02 |
| *Cyanobium* | 0.05 | 0.01 | 9.02 | 12.2 | 38.22 |
| *Prochlorococcus* | 0 | 0.03 | 6.71 | 9.08 | 47.31 |
| *Alteromonas* | 0.03 | 0.03 | 4.92 | 6.66 | 53.97 |
| *Fluviicola* | 0.02 | 0 | 2.96 | 4.01 | 57.98 |
| *Pseudomonas* | 0.01 | 0.01 | 2.44 | 3.30 | 61.27 |
| *Polynucleobacter* | 0.01 | 0 | 2.34 | 3.16 | 64.44 |
| *Roseivivax* | 0.01 | 0.01 | 1.60 | 2.16 | 66.60 |
| *Aureispira* | 0.01 | 0 | 1.51 | 2.04 | 68.63 |
| *Limnohabitans* | 0.01 | 0 | 1.39 | 1.88 | 70.51 |
| *Pseudoalteromonas* | 0.01 | 0.01 | 1.31 | 1.78 | 72.29 |
| *Saprospira* | 0 | 0.01 | 1.26 | 1.70 | 73.99 |
| *Methylomonas* | 0.01 | 0 | 1.20 | 1.63 | 75.62 |
| *Comamonas* | 0.01 | 0 | 1.10 | 1.49 | 77.11 |
| *Magnetospira* | 0.01 | 0 | 0.85 | 1.15 | 78.26 |
| *Acinetobacter* | 0 | 0 | 0.75 | 1.02 | 79.28 |
| *Alcanivorax* | 0 | 0 | 0.69 | 0.94 | 80.21 |
| *Nitrospina* | 0 | 0 | 0.63 | 0.85 | 81.07 |
| *Litorimicrobium* | 0 | 0 | 0.62 | 0.84 | 81.91 |
| *Balneola* | 0 | 0 | 0.60 | 0.82 | 82.73 |
| *Marinobacterium* | 0 | 0 | 0.58 | 0.79 | 83.52 |
| *Grimontia* | 0 | 0 | 0.57 | 0.77 | 84.29 |
| *Microbulbifer* | 0 | 0 | 0.56 | 0.75 | 85.04 |
| *Limnobacter* | 0 | 0 | 0.54 | 0.73 | 85.77 |
| *Ketogulonicigenium* | 0 | 0 | 0.51 | 0.69 | 86.46 |
| *Candidatus Puniceispirillum* | 0 | 0 | 0.46 | 0.62 | 87.08 |
| *Crocinitomix* | 0 | 0 | 0.43 | 0.59 | 87.67 |
| *Aestuariibacter* | 0 | 0 | 0.42 | 0.57 | 88.24 |
| *Coxiella* | 0 | 0 | 0.41 | 0.56 | 88.80 |
| *Rheinheimera* | 0 | 0 | 0.35 | 0.48 | 89.28 |
| *Ramlibacter* | 0 | 0 | 0.35 | 0.47 | 89.75 |
| *Sediminibacterium* | 0 | 0 | 0.34 | 0.46 | 90.21 |
| *Idiomarina* | 0 | 0 | 0.30 | 0.41 | 90.62 |
| *Winogradskyella* | 0 | 0 | 0.30 | 0.40 | 91.02 |
| *Ottowia* | 0 | 0 | 0.26 | 0.35 | 91.37 |
| *Flavobacterium* | 0 | 0 | 0.24 | 0.32 | 91.69 |
| *Oceanobacter* | 0 | 0 | 0.21 | 0.28 | 91.97 |
| *Lautropia* | 0 | 0 | 0.20 | 0.28 | 92.25 |
| *Arcobacter* | 0 | 0 | 0.19 | 0.26 | 92.50 |
| *Bdellovibrio* | 0 | 0 | 0.18 | 0.25 | 92.75 |
| *Chthoniobacter* | 0 | 0 | 0.17 | 0.23 | 92.98 |
| *Terrimonas* | 0 | 0 | 0.15 | 0.2 | 93.18 |
| *Tenacibaculum* | 0 | 0 | 0.15 | 0.2 | 93.39 |
| *Marinomonas* | 0 | 0 | 0.14 | 0.19 | 93.58 |
| *Noviherbaspirillum* | 0 | 0 | 0.14 | 0.19 | 93.76 |
| *Curvibacter* | 0 | 0 | 0.14 | 0.18 | 93.95 |
| *Vibrio* | 0 | 0 | 0.13 | 0.17 | 94.12 |
| *Lutibacter* | 0 | 0 | 0.11 | 0.15 | 94.27 |
| *Bryobacter* | 0 | 0 | 0.11 | 0.15 | 94.42 |
| *Candidatus Aquiluna* | 0 | 0 | 0.11 | 0.15 | 94.56 |
| *Tropicimonas* | 0 | 0 | 0.11 | 0.15 | 94.71 |
| *Candidatus Endoecteinascidia* | 0 | 0 | 0.11 | 0.14 | 94.85 |
| *Erythrobacter* | 0 | 0 | 0.10 | 0.14 | 94.99 |
| *Thalassolituus* | 0 | 0 | 0.10 | 0.14 | 95.12 |
| *Occidentia* | 0 | 0 | 0.09 | 0.13 | 95.25 |
| *Bermanella* | 0 | 0 | 0.09 | 0.12 | 95.37 |
| *Oleiphilus* | 0 | 0 | 0.09 | 0.12 | 95.49 |
| *Alishewanella* | 0 | 0 | 0.09 | 0.12 | 95.61 |
| *Methylomarinum* | 0 | 0 | 0.08 | 0.11 | 95.72 |
| *Robiginitalea* | 0 | 0 | 0.08 | 0.11 | 95.83 |
| *Sphingomonas* | 0 | 0 | 0.07 | 0.10 | 95.92 |
| *Maricaulis* | 0 | 0 | 0.07 | 0.09 | 96.01 |
| *Reyranella* | 0 | 0 | 0.07 | 0.09 | 96.11 |
| *Novosphingobium* | 0 | 0 | 0.06 | 0.09 | 96.19 |
| *Lewinella* | 0 | 0 | 0.06 | 0.08 | 96.28 |
| *Pseudarcicella* | 0 | 0 | 0.06 | 0.08 | 96.36 |
| *Weissella* | 0 | 0 | 0.06 | 0.08 | 96.44 |
| *Truepera* | 0 | 0 | 0.06 | 0.08 | 96.51 |
| *Candidatus Solibacter* | 0 | 0 | 0.06 | 0.08 | 96.59 |
| *Salinimicrobium* | 0 | 0 | 0.05 | 0.07 | 96.66 |
| *Bauldia* | 0 | 0 | 0.05 | 0.07 | 96.73 |
| *Halomonas* | 0 | 0 | 0.05 | 0.06 | 96.79 |
| *Formosa* | 0 | 0 | 0.05 | 0.06 | 96.86 |
| *Filomicrobium* | 0 | 0 | 0.05 | 0.06 | 96.92 |
| *Salinihabitans* | 0 | 0 | 0.05 | 0.06 | 96.98 |
| *Hydrogenophaga* | 0 | 0 | 0.04 | 0.06 | 97.04 |
| *Algoriphagus* | 0 | 0 | 0.04 | 0.06 | 97.10 |
| *Bacillus* | 0 | 0 | 0.04 | 0.06 | 97.16 |
| *Roseomonas* | 0 | 0 | 0.04 | 0.06 | 97.22 |
| *Massilia* | 0 | 0 | 0.04 | 0.05 | 97.27 |
| *Draconibacterium* | 0 | 0 | 0.04 | 0.05 | 97.32 |
| *Verrucomicrobium* | 0 | 0 | 0.04 | 0.05 | 97.37 |
| *Staphylococcus* | 0 | 0 | 0.04 | 0.05 | 97.42 |
| *Rhodobacter* | 0 | 0 | 0.04 | 0.05 | 97.47 |
| *Acholeplasma* | 0 | 0 | 0.03 | 0.05 | 97.52 |
| *Lacibacter* | 0 | 0 | 0.03 | 0.05 | 97.57 |
| *Nitrospira* | 0 | 0 | 0.03 | 0.05 | 97.61 |
| *Coraliomargarita* | 0 | 0 | 0.03 | 0.04 | 97.66 |
| *Haliscomenobacter* | 0 | 0 | 0.03 | 0.04 | 97.70 |
| *Opitutus* | 0 | 0 | 0.03 | 0.04 | 97.74 |
| *Paracoccus* | 0 | 0 | 0.03 | 0.04 | 97.79 |
| *Rhodoferax* | 0 | 0 | 0.03 | 0.04 | 97.83 |
| *Endozoicomonas* | 0 | 0 | 0.03 | 0.04 | 97.87 |
| *Algicola* | 0 | 0 | 0.03 | 0.04 | 97.91 |
| *Methylocystis* | 0 | 0 | 0.03 | 0.04 | 97.96 |
| *Salinimonas* | 0 | 0 | 0.03 | 0.04 | 98.00 |
| *Rhodovulum* | 0 | 0 | 0.03 | 0.04 | 98.04 |
| *Tropicibacter* | 0 | 0 | 0.03 | 0.04 | 98.08 |
| *Sphingobium* | 0 | 0 | 0.03 | 0.04 | 98.12 |
| *Delftia* | 0 | 0 | 0.03 | 0.04 | 98.16 |
| *Zunongwangia* | 0 | 0 | 0.03 | 0.04 | 98.20 |
| *Empedobacter* | 0 | 0 | 0.03 | 0.04 | 98.23 |
| *Fangia* | 0 | 0 | 0.03 | 0.04 | 98.27 |
| *Streptococcus* | 0 | 0 | 0.03 | 0.04 | 98.31 |
| *Geobacter* | 0 | 0 | 0.03 | 0.04 | 98.34 |
| *Anaeromyxobacter* | 0 | 0 | 0.03 | 0.04 | 98.38 |
| *Azospirillum* | 0 | 0 | 0.03 | 0.03 | 98.41 |
| *Tranquillimonas* | 0 | 0 | 0.03 | 0.03 | 98.45 |
| *Marivita* | 0 | 0 | 0.03 | 0.03 | 98.48 |
| *Pontibacter* | 0 | 0 | 0.02 | 0.03 | 98.51 |
| *Enterovibrio* | 0 | 0 | 0.02 | 0.03 | 98.55 |
| *Methylocaldum* | 0 | 0 | 0.02 | 0.03 | 98.58 |
| *Shewanella* | 0 | 0 | 0.02 | 0.03 | 98.61 |
| *Marinobacter* | 0 | 0 | 0.02 | 0.03 | 98.64 |
| *Sulfitobacter* | 0 | 0 | 0.02 | 0.03 | 98.66 |
| *Altererythrobacter* | 0 | 0 | 0.02 | 0.03 | 98.69 |
| *Aquimarina* | 0 | 0 | 0.02 | 0.03 | 98.72 |
| *Roseobacter* | 0 | 0 | 0.02 | 0.03 | 98.74 |
| *Bacteroides* | 0 | 0 | 0.02 | 0.03 | 98.77 |
| *Methylocella* | 0 | 0 | 0.02 | 0.03 | 98.80 |
| *Hyphomicrobium* | 0 | 0 | 0.02 | 0.03 | 98.82 |
| *Mesonia* | 0 | 0 | 0.02 | 0.03 | 98.85 |
| *Neptunomonas* | 0 | 0 | 0.02 | 0.02 | 98.87 |
| *Thalassococcus* | 0 | 0 | 0.02 | 0.02 | 98.90 |
| *Sphingobacterium* | 0 | 0 | 0.02 | 0.02 | 98.92 |
| *Psychrobacter* | 0 | 0 | 0.02 | 0.02 | 98.95 |
| *Terasakiella* | 0 | 0 | 0.02 | 0.02 | 98.97 |
| *Geothrix* | 0 | 0 | 0.02 | 0.02 | 98.99 |
| *Salegentibacter* | 0 | 0 | 0.02 | 0.02 | 99.02 |
| *Brevundimonas* | 0 | 0 | 0.02 | 0.02 | 99.04 |
| *Kordiimonas* | 0 | 0 | 0.02 | 0.02 | 99.06 |
| *Ferriphaselus* | 0 | 0 | 0.02 | 0.02 | 99.08 |
| *Beggiatoa* | 0 | 0 | 0.02 | 0.02 | 99.10 |
| *Rhodopirellula* | 0 | 0 | 0.01 | 0.02 | 99.12 |
| *Saccharophagus* | 0 | 0 | 0.01 | 0.02 | 99.14 |
| *Caulobacter* | 0 | 0 | 0.01 | 0.02 | 99.16 |
| *Propionigenium* | 0 | 0 | 0.01 | 0.02 | 99.18 |
| *Erysipelotrichaceae* | 0 | 0 | 0.01 | 0.02 | 99.20 |
| *Reinekea* | 0 | 0 | 0.01 | 0.02 | 99.22 |
| *Sandaracinus* | 0 | 0 | 0.01 | 0.02 | 99.23 |
| *Polaromonas* | 0 | 0 | 0.01 | 0.02 | 99.25 |
| *Cetobacterium* | 0 | 0 | 0.01 | 0.02 | 99.27 |
| *Aeromonas* | 0 | 0 | 0.01 | 0.02 | 99.28 |
| *Actibacterium* | 0 | 0 | 0.01 | 0.02 | 99.30 |
| *Gramella* | 0 | 0 | 0.01 | 0.02 | 99.31 |
| *Sphaerotilus* | 0 | 0 | 0.01 | 0.02 | 99.33 |
| *Ulvibacter* | 0 | 0 | 0.01 | 0.02 | 99.35 |
| *Microscilla* | 0 | 0 | 0.01 | 0.02 | 99.36 |
| *Neptuniibacter* | 0 | 0 | 0.01 | 0.01 | 99.38 |
| *Sulfuricurvum* | 0 | 0 | 0.01 | 0.01 | 99.39 |
| *Ideonella* | 0 | 0 | 0.01 | 0.01 | 99.41 |
| *Croceicoccus* | 0 | 0 | 0.01 | 0.01 | 99.42 |
| *Sideroxydans* | 0 | 0 | 0.01 | 0.01 | 99.43 |
| *Phenylobacterium* | 0 | 0 | 0.01 | 0.01 | 99.44 |
| *Pedobacter* | 0 | 0 | 0.01 | 0.01 | 99.46 |
| *Leptospira* | 0 | 0 | 0.01 | 0.01 | 99.47 |
| *Lysinibacillus* | 0 | 0 | 0.01 | 0.01 | 99.48 |
| *Salinisphaera* | 0 | 0 | 0.01 | 0.01 | 99.49 |
| *Pseudoruegeria* | 0 | 0 | 0.01 | 0.01 | 99.50 |
| *Magnetospirillum* | 0 | 0 | 0.01 | 0.01 | 99.52 |
| *Cellvibrio* | 0 | 0 | 0.01 | 0.01 | 99.53 |
| *Thalassotalea* | 0 | 0 | 0.01 | 0.01 | 99.54 |
| *Niveispirillum* | 0 | 0 | 0.01 | 0.01 | 99.55 |
| *Legionella* | 0 | 0 | 0.01 | 0.01 | 99.56 |
| *Henriciella* | 0 | 0 | 0.01 | 0.01 | 99.57 |
| *Candidatus Symbiobacter* | 0 | 0 | 0.01 | 0.01 | 99.58 |
| *Thiothrix* | 0 | 0 | 0.01 | 0.01 | 99.59 |
| *Chryseobacterium* | 0 | 0 | 0.01 | 0.01 | 99.60 |
| *Aquabacterium* | 0 | 0 | 0.01 | 0.01 | 99.61 |
| *Rubrivivax* | 0 | 0 | 0.01 | 0.01 | 99.62 |
| *Sulfurovum* | 0 | 0 | 0.01 | 0.01 | 99.62 |
| *Thalassospira* | 0 | 0 | 0.01 | 0.01 | 99.63 |
| *Synechocystis* | 0 | 0 | 0.01 | 0.01 | 99.64 |
| *Stenotrophomonas* | 0 | 0 | 0.01 | 0.01 | 99.65 |
| *Methyloglobulus* | 0 | 0 | 0.01 | 0.01 | 99.66 |
| *Sulfurospirillum* | 0 | 0 | 0.01 | 0.01 | 99.67 |
| *Chromohalobacter* | 0 | 0 | 0.01 | 0.01 | 99.68 |
| *Leisingera* | 0 | 0 | 0.01 | 0.01 | 99.68 |
| *Blastopirellula* | 0 | 0 | 0.01 | 0.01 | 99.69 |
| *Sulfurimonas* | 0 | 0 | 0.01 | 0.01 | 99.70 |
| *Thiovulum* | 0 | 0 | 0.01 | 0.01 | 99.71 |
| *Zoogloea* | 0 | 0 | 0.01 | 0.01 | 99.71 |
| *Rhodoluna* | 0 | 0 | 0.01 | 0.01 | 99.72 |
| *Domibacillus* | 0 | 0 | 0.01 | 0.01 | 99.73 |
| *Desulfopila* | 0 | 0 | 0 | 0.01 | 99.74 |
| *Oceanospirillum* | 0 | 0 | 0 | 0.01 | 99.74 |
| *Epibacterium* | 0 | 0 | 0 | 0.01 | 99.75 |
| *Alkanindiges* | 0 | 0 | 0 | 0.01 | 99.76 |
| *Exiguobacterium* | 0 | 0 | 0 | 0.01 | 99.76 |
| *Candidatus Amoebophilus* | 0 | 0 | 0 | 0.01 | 99.77 |
| *Simiduia* | 0 | 0 | 0 | 0.01 | 99.77 |
| *Cloacibacterium* | 0 | 0 | 0 | 0.01 | 99.78 |
| *Emticicia* | 0 | 0 | 0 | 0.01 | 99.78 |
| *Bacteriovorax* | 0 | 0 | 0 | 0 | 99.79 |
| *Oceanicaulis* | 0 | 0 | 0 | 0.01 | 99.79 |
| *Bradyrhizobium* | 0 | 0 | 0 | 0 | 99.80 |
| *Flectobacillus* | 0 | 0 | 0 | 0 | 99.80 |
| *Gemella* | 0 | 0 | 0 | 0 | 99.81 |
| *Spirochaeta* | 0 | 0 | 0 | 0 | 99.81 |
| *Paludibacter* | 0 | 0 | 0 | 0 | 99.82 |
| *Pseudorhodobacter* | 0 | 0 | 0 | 0 | 99.82 |
| *Catenovulum* | 0 | 0 | 0 | 0 | 99.83 |
| *Paenibacillus* | 0 | 0 | 0 | 0 | 99.83 |
| *Escherichia / Shigella* | 0 | 0 | 0 | 0 | 99.84 |
| *Marinovum* | 0 | 0 | 0 | 0 | 99.84 |
| *Neochlamydia* | 0 | 0 | 0 | 0 | 99.84 |
| *Cobetia* | 0 | 0 | 0 | 0 | 99.85 |
| *Rhodocista* | 0 | 0 | 0 | 0 | 99.85 |
| *Anaerococcus* | 0 | 0 | 0 | 0 | 99.86 |
| *Desulfotignum* | 0 | 0 | 0 | 0 | 99.86 |
| *Simkania* | 0 | 0 | 0 | 0 | 99.86 |
| *Gallaecimonas* | 0 | 0 | 0 | 0 | 99.87 |
| *Herbaspirillum* | 0 | 0 | 0 | 0 | 99.87 |
| *Haliangium* | 0 | 0 | 0 | 0 | 99.88 |
| *Jeotgalibacillus* | 0 | 0 | 0 | 0 | 99.88 |
| *Uliginosibacterium* | 0 | 0 | 0 | 0 | 99.88 |
| *Chlorobaculum* | 0 | 0 | 0 | 0 | 99.89 |
| *Marmoricola* | 0 | 0 | 0 | 0 | 99.89 |
| *Spongiibacter* | 0 | 0 | 0 | 0 | 99.89 |
| *Bergeyella* | 0 | 0 | 0 | 0 | 99.90 |
| *Flammeovirga* | 0 | 0 | 0 | 0 | 99.90 |
| *Perlucidibaca* | 0 | 0 | 0 | 0 | 99.90 |
| *Xanthomonas* | 0 | 0 | 0 | 0 | 99.90 |
| *Hyphomonas* | 0 | 0 | 0 | 0 | 99.91 |
| *Mucilaginibacter* | 0 | 0 | 0 | 0 | 99.91 |
| *Prevotellaceae* | 0 | 0 | 0 | 0 | 99.91 |
| *Syntrophus* | 0 | 0 | 0 | 0 | 99.91 |
| *Alistipes* | 0 | 0 | 0 | 0 | 99.92 |
| *Amphritea* | 0 | 0 | 0 | 0 | 99.92 |
| *Leptothrix* | 0 | 0 | 0 | 0 | 99.92 |
| *Celeribacter* | 0 | 0 | 0 | 0 | 99.93 |
| *Desulfobulbus* | 0 | 0 | 0 | 0 | 99.93 |
| *Desulfomonile* | 0 | 0 | 0 | 0 | 99.93 |
| *Rhizobacter* | 0 | 0 | 0 | 0 | 99.93 |
| *Aerococcus* | 0 | 0 | 0 | 0 | 99.94 |
| *Desulfovibrio* | 0 | 0 | 0 | 0 | 99.94 |
| *Lactobacillus* | 0 | 0 | 0 | 0 | 99.94 |
| *Phycisphaera* | 0 | 0 | 0 | 0 | 99.94 |
| *Teredinibacter* | 0 | 0 | 0 | 0 | 99.94 |
| *Candidatus Paracaedibacter* | 0 | 0 | 0 | 0 | 99.95 |
| *Fusobacterium* | 0 | 0 | 0 | 0 | 99.95 |
| *Phaeospirillum* | 0 | 0 | 0 | 0 | 99.95 |
| *Solitalea* | 0 | 0 | 0 | 0 | 99.95 |
| *Turneriella* | 0 | 0 | 0 | 0 | 99.95 |
| *Achromobacter* | 0 | 0 | 0 | 0 | 99.96 |
| *Asticcacaulis* | 0 | 0 | 0 | 0 | 99.96 |
| *Campylobacter* | 0 | 0 | 0 | 0 | 99.96 |
| *Cytophaga* | 0 | 0 | 0 | 0 | 99.96 |
| *Neisseria* | 0 | 0 | 0 | 0 | 99.96 |
| *Sedimenticola* | 0 | 0 | 0 | 0 | 99.96 |
| *Alloprevotella* | 0 | 0 | 0 | 0 | 99.97 |
| *Epulopiscium* | 0 | 0 | 0 | 0 | 99.97 |
| *Hirschia* | 0 | 0 | 0 | 0 | 99.97 |
| *Luteimonas* | 0 | 0 | 0 | 0 | 99.97 |
| *Methylotenera* | 0 | 0 | 0 | 0 | 99.97 |
| *Pirellula* | 0 | 0 | 0 | 0 | 99.97 |
| *Labrenzia* | 0 | 0 | 0 | 0 | 99.98 |
| *Marinococcus* | 0 | 0 | 0 | 0 | 99.98 |
| *Methylophaga* | 0 | 0 | 0 | 0 | 99.98 |
| *Mycobacterium* | 0 | 0 | 0 | 0 | 99.98 |
| *Polycyclovorans* | 0 | 0 | 0 | 0 | 99.98 |
| *Prevotella* | 0 | 0 | 0 | 0 | 99.98 |
| *Thalassobaculum* | 0 | 0 | 0 | 0 | 99.98 |
| *Xylophilus* | 0 | 0 | 0 | 0 | 99.98 |
| *Candidatus Nitrosopumilus* | 0 | 0 | 0 | 0 | 99.99 |
| *Corynebacterium* | 0 | 0 | 0 | 0 | 99.99 |
| *Dysgonomonas* | 0 | 0 | 0 | 0 | 99.99 |
| *Kiloniella* | 0 | 0 | 0 | 0 | 99.99 |
| *Kordia* | 0 | 0 | 0 | 0 | 99.99 |
| *Methylomicrobium* | 0 | 0 | 0 | 0 | 99.99 |
| *Nannocystis* | 0 | 0 | 0 | 0 | 99.99 |
| *Nevskia* | 0 | 0 | 0 | 0 | 99.99 |
| *Simplicispira* | 0 | 0 | 0 | 0 | 99.99 |
| *Afipia* | 0 | 0 | 0 | 0 | 100 |
| *Candidatus Odyssella* | 0 | 0 | 0 | 0 | 100 |
| *Cellulosimicrobium* | 0 | 0 | 0 | 0 | 100 |
| *Desulfosarcina* | 0 | 0 | 0 | 0 | 100 |
| *Francisella* | 0 | 0 | 0 | 0 | 100 |
| *Fulvivirga* | 0 | 0 | 0 | 0 | 100 |
| *Leeuwenhoekiella* | 0 | 0 | 0 | 0 | 100 |
| *Lysobacter* | 0 | 0 | 0 | 0 | 100 |
| *Magnetococcus* | 0 | 0 | 0 | 0 | 100 |
| *Peptoniphilus* | 0 | 0 | 0 | 0 | 100 |
| *Selenomonas* | 0 | 0 | 0 | 0 | 100 |
| *Succinivibrio* | 0 | 0 | 0 | 0 | 100 |

**Table S4. SIMPER results for community-weighted means (CWMs) of metabolic traits of sample clusters along the transect.** M_group1_ = mean of CWM values by trait at group 1 (St2 to St7), M_group2_ = mean of CWM values by trait at group 2 (St9 to St15). AD = average dissimilarity of typifying traits in each sample, contribution (C%) = percentage contribution to dissimilarity for each trait, cumulative (Cu%) = cumulative percentage of dissimilarity. The overall average dissimilarity was equal to 8.33%.

| **CMWs** | **M_Group1_** | **M_Group2_** | **AD** | **C%** | **Cu%** |
| --- | --- | --- | --- | --- | --- |
| Porphyrin and chlorophyll metabolism (PCHlM) | 4.06 | 6.03 | 1.15 | 13.82 | 13.82 |
| Benzoate degradation (BD) | 2.27 | 1.48 | 0.50 | 5.96 | 19.78 |
| Valine leucine and isoleucine degradation (VLID) | 2.91 | 2.12 | 0.49 | 5.83 | 25.61 |
| Carbon fixation pathways in prokaryotes (CFPP) | 3.61 | 2.93 | 0.41 | 4.90 | 30.51 |
| Butanoate metabolism (BM) | 3.04 | 2.38 | 0.38 | 4.60 | 35.10 |
| Oxidative phosphorylation (OP) | 3.93 | 4.22 | 0.38 | 4.58 | 39.69 |
| Fatty acid degradation (FAD) | 1.71 | 1.17 | 0.31 | 3.76 | 43.45 |
| Tryptophan metabolism (TM) | 1.53 | 1.02 | 0.30 | 3.58 | 47.03 |
| Lipopolysaccharide biosynthesis (LB) | 2.28 | 1.82 | 0.29 | 3.46 | 50.50 |
| Pyruvate metabolism (PM) | 3.96 | 3.54 | 0.29 | 3.43 | 53.93 |
| Phenylalanine metabolism (PAM) | 2.01 | 1.55 | 0.28 | 3.33 | 57.26 |
| Ubiquinone and other terpenoid quinone biosynthesis (UTQB) | 1.68 | 2.14 | 0.27 | 3.21 | 60.47 |
| Amino sugar and nucleotide sugar metabolism (ASNSM) | 3.96 | 4.44 | 0.27 | 3.19 | 63.66 |
| Starch and sucrose metabolism (SSM) | 2.01 | 2.41 | 0.24 | 2.87 | 66.53 |
| Aminoacyl tRNA biosynthesis (ATB) | 3.19 | 3.57 | 0.24 | 2.86 | 69.39 |
| Carbon fixation in photosynthetic organisms (CFPO) | 2.14 | 2.46 | 0.2 | 2.43 | 71.82 |
| Cationic antimicrobial peptide CAMP resistance (CAPCR) | 3.00 | 2.73 | 0.19 | 2.33 | 74.15 |
| Cell cycle (CC) | 2.22 | 2.00 | 0.16 | 1.90 | 76.05 |
| Phenylalanine tyrosine and tryptophan biosynthesis (PTTB) | 2.57 | 2.86 | 0.15 | 1.81 | 77.85 |
| Beta lactam resistance (BLR) | 2.17 | 1.97 | 0.14 | 1.66 | 79.51 |
| Folate biosynthesis (FB) | 2.01 | 2.28 | 0.14 | 1.63 | 81.15 |
| Base excision repair (BER) | 2.29 | 2.50 | 0.13 | 1.57 | 82.71 |
| Sulfur metabolism (SM) | 3.14 | 3.27 | 0.12 | 1.48 | 84.20 |
| Cysteine and methionine metabolism (CMM) | 4.29 | 4.46 | 0.12 | 1.46 | 85.65 |
| Quorum sensing (QS) | 4.20 | 4.20 | 0.11 | 1.32 | 86.98 |
| Citrate cycle TCA cycle (CCTC) | 3.87 | 4.03 | 0.11 | 1.30 | 88.28 |
| Fatty acid biosynthesis (FAB) | 2.37 | 2.29 | 0.10 | 1.21 | 89.49 |
| Alanine aspartate and glutamate metabolism (AAGM) | 2.83 | 3.00 | 0.10 | 1.21 | 90.69 |
| Peptidoglycan biosynthesis (PB) | 1.88 | 1.98 | 0.10 | 1.16 | 91.85 |
| Arginine and proline metabolism (APM) | 2.57 | 2.68 | 0.09 | 1.11 | 92.96 |
| ABC transporters (AT) | 1.78 | 1.68 | 0.09 | 1.10 | 94.06 |
| Pantothenate and CoA biosynthesis (PCB) | 1.97 | 2.13 | 0.09 | 1.03 | 95.09 |
| Nitrogen metabolism (NM) | 2.21 | 2.05 | 0.08 | 0.99 | 96.08 |
| mRNA surveillance pathway (MSP) | 1.61 | 1.64 | 0.07 | 0.85 | 96.94 |
| Nicotinate and nicotinamide metabolism (NNM) | 2.30 | 2.42 | 0.07 | 0.82 | 97.76 |
| Terpenoid backbone biosynthesis (TBB) | 1.91 | 1.95 | 0.06 | 0.78 | 98.54 |
| Sulfur relay system (SRS) | 1.63 | 1.68 | 0.06 | 0.74 | 99.28 |
| Methane metabolism (MM) | 2.89 | 2.88 | 0.06 | 0.72 | 100 |

**Table S5. SIMPER results for community-weighted means (CWMs) of metabolic traits of sample clusters over depth.** M_B_ = mean of CWM values by trait at 11 m depth, M_S_ = mean of CWM values by trait at 0.5 m depth. AD = average dissimilarity of typifying traits in each sample, contribution (C%) = percentage contribution to dissimilarity for each trait, cumulative (Cu%) = cumulative percentage of dissimilarity. The overall average dissimilarity = 8.14%.

| **CMWs** | **M_B_** | **M_S_** | **AD** | **C%** | **Cu%** |
| --- | --- | --- | --- | --- | --- |
| Porphyrin and chlorophyll metabolism (PCHlM) | 4.32 | 5.77 | 0.90 | 11.06 | 11.06 |
| Oxidative phosphorylation (OP) | 3.45 | 4.70 | 0.68 | 8.38 | 19.44 |
| Benzoate degradation (BD) | 2.07 | 1.68 | 0.43 | 5.23 | 24.67 |
| Valine leucine and isoleucine degradation (VLID) | 2.74 | 2.29 | 0.40 | 4.96 | 29.62 |
| Cationic antimicrobial peptide CAMP resistance (CAPCR) | 3.20 | 2.53 | 0.34 | 4.16 | 33.78 |
| Butanoate metabolism (BM) | 2.89 | 2.53 | 0.34 | 4.12 | 37.9 |
| Aminoacyl tRNA biosynthesis (ATB) | 3.08 | 3.68 | 0.31 | 3.84 | 41.74 |
| Carbon fixation in photosynthetic organisms (CFPO) | 2.04 | 2.57 | 0.29 | 3.56 | 45.30 |
| Quorum sensing (QS) | 4.48 | 3.93 | 0.27 | 3.37 | 48.67 |
| Beta lactam resistance (BLR) | 2.34 | 1.80 | 0.27 | 3.31 | 51.98 |
| Fatty acid degradation (FAD) | 1.63 | 1.25 | 0.27 | 3.26 | 55.24 |
| Carbon fixation pathways in prokaryotes (CFPP) | 3.36 | 3.18 | 0.26 | 3.18 | 58.42 |
| Phenylalanine metabolism (PAM) | 1.93 | 1.63 | 0.25 | 3.02 | 61.44 |
| Tryptophan metabolism (TM) | 1.44 | 1.11 | 0.24 | 2.90 | 64.34 |
| Pyruvate metabolism (PM) | 3.70 | 3.80 | 0.23 | 2.84 | 67.18 |
| Ubiquinone and other terpenoid quinone biosynthesis (UTQB) | 1.80 | 2.03 | 0.21 | 2.53 | 69.72 |
| Lipopolysaccharide biosynthesis (LB) | 2.14 | 1.97 | 0.19 | 2.33 | 72.04 |
| Starch and sucrose metabolism (SSM) | 2.16 | 2.25 | 0.19 | 2.28 | 74.32 |
| Amino sugar and nucleotide sugar metabolism (ASNSM) | 4.15 | 4.25 | 0.18 | 2.26 | 76.58 |
| Terpenoid backbone biosynthesis (TBB) | 1.76 | 2.10 | 0.17 | 2.11 | 78.69 |
| Sulfur metabolism (SM) | 3.14 | 3.27 | 0.13 | 1.65 | 80.34 |
| Citrate cycle TCA cycle (CCTC) | 4.07 | 3.83 | 0.13 | 1.64 | 81.97 |
| Peptidoglycan biosynthesis (PB) | 1.83 | 2.03 | 0.12 | 1.50 | 83.48 |
| Alanine aspartate and glutamate metabolism (AAGM) | 3.03 | 2.80 | 0.12 | 1.42 | 84.89 |
| Cysteine and methionine metabolism (CMM) | 4.35 | 4.40 | 0.11 | 1.39 | 86.28 |
| ABC transporters (AT) | 1.64 | 1.82 | 0.11 | 1.34 | 87.62 |
| mRNA surveillance pathway (MSP) | 1.72 | 1.53 | 0.11 | 1.31 | 88.93 |
| Folate biosynthesis (FB) | 2.21 | 2.08 | 0.11 | 1.31 | 90.23 |
| Cell cycle (CC) | 2.19 | 2.03 | 0.10 | 1.23 | 91.46 |
| Phenylalanine tyrosine and tryptophan biosynthesis (PTTB) | 2.69 | 2.74 | 0.10 | 1.18 | 92.64 |
| Fatty acid biosynthesis (FAB) | 2.30 | 2.36 | 0.10 | 1.17 | 93.81 |
| Arginine and proline metabolism (APM) | 2.70 | 2.55 | 0.09 | 1.15 | 94.96 |
| Base excision repair (BER) | 2.43 | 2.36 | 0.08 | 1.03 | 95.99 |
| Methane metabolism (MM) | 2.82 | 2.94 | 0.08 | 1.02 | 97.01 |
| Sulfur relay system (SRS) | 1.71 | 1.60 | 0.07 | 0.86 | 97.87 |
| Pantothenate and CoA biosynthesis (PCB) | 2.00 | 2.11 | 0.07 | 0.85 | 98.72 |
| Nitrogen metabolism (NM) | 2.13 | 2.13 | 0.05 | 0.65 | 99.37 |
| Nicotinate and nicotinamide metabolism (NNM) | 2.36 | 2.36 | 0.05 | 0.63 | 100 |

**Table S6. Results of the fourth-corner analysis. All bivariate associations between the environmental variables and traits are given.** Positive and negative significant associations are indicated by asterisks (P_adjusted_ < 0.05 = *, P_adjusted_ < 0.01 = **). Not statistically significant (n.s.) associations are also shown. DO = dissolved oxygen.

| **Traits** | **Environ. Variable** | **Obs.** | **Std. Obs.** | **Alter** | **P_adj_** |
| --- | --- | --- | --- | --- | --- |
| Methane metabolism (MM) | Station | r = 0.371 | 2.846 | two-sided | 0.049* |
|  | Temp | r = 0.021 | 0.234 | two-sided | 0.880^n.s.^ |
|  | Depth | r = 0.109 | 0.926 | two-sided | 0.441^n.s.^ |
|  | Salinity | r = 0.375 | 2.861 | two-sided | 0.009** |
|  | DO | r = -0.230 | -3.224 | two-sided | 0.054^n.s.^ |
| Carbon fixation in photosynthetic organisms (CFPO) | Station | r = 0.410 | 3.425 | two-sided | 0.012* |
|  | Temp | r = -0.017 | -0.158 | two-sided | 0.908^n.s.^ |
|  | Depth | r = 0.009 | 0.072 | two-sided | 0.951^n.s.^ |
|  | Salinity | r = 0.350 | 2.880 | two-sided | 0.010* |
|  | DO | r = -0.235 | -3.600 | two-sided | 0.049* |
| Carbon fixation pathways in prokaryotes (CFPP) | Station | r = 0.249 | 1.863 | two-sided | 0.163^n.s.^ |
|  | Temp | r = 0.095 | 1.164 | two-sided | 0.357^n.s.^ |
|  | Depth | r = 0.260 | 2.113 | two-sided | 0.082^n.s.^ |
|  | Salinity | r = 0.345 | 2.546 | two-sided | 0.023* |
|  | DO | r = -0.135 | -1.640 | two-sided | 0.157^n.s.^ |
| Porphyrin and chlorophyll metabolism (PCHlM) | Station | r = 0.446 | 3.836 | two-sided | 0.009** |
|  | Temp | r = -0.061 | -0.546 | two-sided | 0.688^n.s.^ |
|  | Depth | r = -0.053 | -0.466 | two-sided | 0.723^n.s.^ |
|  | Salinity | r = 0.350 | 3.179 | two-sided | 0.009** |
|  | DO | r = -0.280 | -2.536 | two-sided | 0.036* |
| Nitrogen metabolism (NM) | Station | r = 0.318 | 2.518 | two-sided | 0.066^n.s.^ |
|  | Temp | r = 0.052 | 0.605 | two-sided | 0.669^n.s.^ |
|  | Depth | r = 0.160 | 1.382 | two-sided | 0.226^n.s.^ |
|  | Salinity | r = 0.367 | 2.865 | two-sided | 0.009** |
|  | DO | r = -0.202 | -2.926 | two-sided | 0.063^n.s.^ |
| Sulfur metabolism (SM) | Station | r = 0.369 | 2.954 | two-sided | 0.049* |
|  | Temp | r = 0.027 | 0.299 | two-sided | 0.848^n.s.^ |
|  | Depth | r = 0.114 | 0.991 | two-sided | 0.398^n.s.^ |
|  | Salinity | r = 0.382 | 3.012 | two-sided | 0.007** |
|  | DO | r = -0.233 | -3.394 | two-sided | 0.051^n.s.^ |
| Sulfur relay system (SRS) | Station | r = 0.339 | 2.789 | two-sided | 0.057^n.s.^ |
|  | Temp | r = 0.053 | 0.599 | two-sided | 0.672^n.s.^ |
|  | Depth | r = 0.172 | 1.547 | two-sided | 0.166^n.s.^ |
|  | Salinity | r = 0.379 | 3.058 | two-sided | 0.007** |
|  | DO | r = -0.209 | -3.155 | two-sided | 0.061^n.s.^ |
| Cell cycle (CC) | Station | r = 0.306 | 2.352 | two-sided | 0.079^n.s.^ |
|  | Temp | r = 0.078 | 0.893 | two-sided | 0.495^n.s.^ |
|  | Depth | r = 0.227 | 1.913 | two-sided | 0.098^n.s.^ |
|  | Salinity | r = 0.381 | 2.891 | two-sided | 0.009** |
|  | DO | r = -0.179 | -2.524 | two-sided | 0.082^n.s.^ |
| Ubiquinone and other terpenoid quinone biosynthesis (UTQB) | Station | r = 0.418 | 3.599 | two-sided | 0.009** |
|  | Temp | r = -0.016 | -0.142 | two-sided | 0.912^n.s.^ |
|  | Depth | r = 0.040 | 0.364 | two-sided | 0.787^n.s.^ |
|  | Salinity | r = 0.376 | 3.191 | two-sided | 0.007** |
|  | DO | r = -0.264 | -2.575 | two-sided | 0.032* |
| Oxidative phosphorylation (OP) | Station | r = 0.387 | 3.147 | two-sided | 0.034* |
|  | Temp | r = -0.047 | -0.446 | two-sided | 0.752^n.s.^ |
|  | Depth | r = -0.062 | -0.566 | two-sided | 0.666^n.s.^ |
|  | Salinity | r = 0.299 | 2.434 | two-sided | 0.038* |
|  | DO | r = -0.262 | -2.536 | two-sided | 0.034* |
| Starch and sucrose metabolism (SSM) | Station | r = 0.383 | 3.350 | two-sided | 0.036* |
|  | Temp | r = 0.017 | 0.192 | two-sided | 0.901^n.s.^ |
|  | Depth | r = 0.087 | 0.818 | two-sided | 0.495^n.s.^ |
|  | Salinity | r = 0.378 | 3.220 | two-sided | 0.006** |
|  | DO | r = -0.243 | -3.856 | two-sided | 0.045* |
| Pyruvate metabolism (PM) | Station | r = 0.300 | 2.315 | two-sided | 0.083^n.s.^ |
|  | Temp | r = 0.075 | 0.896 | two-sided | 0.495^n.s.^ |
|  | Depth | r = 0.203 | 1.702 | two-sided | 0.129^n.s.^ |
|  | Salinity | r = 0.367 | 2.785 | two-sided | 0.011* |
|  | DO | r = -0.157 | -1.855 | two-sided | 0.105^n.s.^ |
| Butanoate metabolism (BM) | Station | r = 0.219 | 1.650 | two-sided | 0.199^n.s.^ |
|  | Temp | r = 0.110 | 1.339 | two-sided | 0.268^n.s.^ |
|  | Depth | r = 0.301 | 2.428 | two-sided | 0.063^n.s.^ |
|  | Salinity | r = 0.348 | 2.549 | two-sided | 0.018* |
|  | DO | r = -0.111 | -1.339 | two-sided | 0.261^n.s.^ |
| Pantothenate and CoA biosynthesis (PCB) | Station | r = 0.400 | 3.114 | two-sided | 0.021* |
|  | Temp | r = 0.016 | 0.172 | two-sided | 0.908^n.s.^ |
|  | Depth | r = 0.118 | 1.009 | two-sided | 0.390^n.s.^ |
|  | Salinity | r = 0.398 | 3.084 | two-sided | 0.006** |
|  | DO | r = -0.237 | -3.384 | two-sided | 0.049* |
| Alanine aspartate and glutamate metabolism (AAGM) | Station | r = 0.354 | 2.820 | two-sided | 0.054^n.s.^ |
|  | Temp | r = 0.056 | 0.615 | two-sided | 0.666^n.s.^ |
|  | Depth | r = 0.198 | 1.730 | two-sided | 0.121^n.s.^ |
|  | Salinity | r = 0.406 | 4.395 | two-sided | 0.006** |
|  | DO | r = -0.206 | -3.014 | two-sided | 0.061^n.s.^ |
| Cysteine and methionine metabolism (CMM) | Station | r = 0.371 | 2.967 | two-sided | 0.049* |
|  | Temp | r = 0.035 | 0.382 | two-sided | 0.802^n.s.^ |
|  | Depth | r = 0.139 | 1.220 | two-sided | 0.287^n.s.^ |
|  | Salinity | r = 0.391 | 3.091 | two-sided | 0.007** |
|  | DO | r = -0.229 | -3.339 | two-sided | 0.054^n.s.^ |
| Valine leucine and isoleucine degradation (VLID) | Station | r = 0.136 | 1.086 | two-sided | 0.298^n.s.^ |
|  | Temp | r = 0.136 | 1.644 | two-sided | 0.159^n.s.^ |
|  | Depth | r = 0.343 | 2.793 | two-sided | 0.039* |
|  | Salinity | r = 0.295 | 2.212 | two-sided | 0.051^n.s.^ |
|  | DO | r = -0.051 | -0.615 | two-sided | 0.656^n.s.^ |
| Arginine and proline metabolism (APM) | Station | r = 0.345 | 2.751 | two-sided | 0.058^n.s.^ |
|  | Temp | r = 0.068 | 0.742 | two-sided | 0.591^n.s.^ |
|  | Depth | r = 0.197 | 1.709 | two-sided | 0.121^n.s.^ |
|  | Salinity | r = 0.415 | 4.483 | two-sided | 0.006** |
|  | DO | r = -0.206 | -3.007 | two-sided | 0.063^n.s.^ |
| Phenylalanine metabolism (PAM) | Station | r = 0.177 | 1.376 | two-sided | 0.245^n.s.^ |
|  | Temp | r = 0.124 | 1.501 | two-sided | 0.202^n.s.^ |
|  | Depth | r = 0.328 | 2.688 | two-sided | 0.049* |
|  | Salinity | r = 0.322 | 2.406 | two-sided | 0.036* |
|  | DO | r = -0.080 | -0.960 | two-sided | 0.444^n.s.^ |
| Tryptophan metabolism (TM) | Station | r = 0.079 | 0.954 | two-sided | 0.453^n.s.^ |
|  | Temp | r = 0.153 | 1.830 | two-sided | 0.119^n.s.^ |
|  | Depth | r = 0.362 | 2.986 | two-sided | 0.017* |
|  | Salinity | r = 0.263 | 2.003 | two-sided | 0.093^n.s.^ |
|  | DO | r = -0.019 | -0.218 | two-sided | 0.880^n.s.^ |
| Phenylalanine tyrosine and tryptophan biosynthesis (PTTB) | Station | r = 0.396 | 3.133 | two-sided | 0.023* |
|  | Temp | r = 0.024 | 0.261 | two-sided | 0.868^n.s.^ |
|  | Depth | r = 0.138 | 1.206 | two-sided | 0.292^n.s.^ |
|  | Salinity | r = 0.406 | 3.187 | two-sided | 0.006** |
|  | DO | r = -0.233 | -3.369 | two-sided | 0.051^n.s.^ |
| Benzoate degradation (BD) | Station | r = 0.069 | 0.845 | two-sided | 0.512^n.s.^ |
|  | Temp | r = 0.144 | 1.711 | two-sided | 0.141^n.s.^ |
|  | Depth | r = 0.372 | 3.005 | two-sided | 0.011* |
|  | Salinity | r = 0.259 | 1.953 | two-sided | 0.103^n.s.^ |
|  | DO | r = 0.003 | 0.043 | two-sided | 0.967^n.s.^ |
| Beta Lactam resistance (BLR) | Station | r = 0.238 | 1.968 | two-sided | 0.147^n.s.^ |
|  | Temp | r = 0.118 | 1.376 | two-sided | 0.253^n.s.^ |
|  | Depth | r = 0.304 | 2.701 | two-sided | 0.053^n.s.^ |
|  | Salinity | r = 0.377 | 3.010 | two-sided | 0.007** |
|  | DO | r = -0.136 | -1.578 | two-sided | 0.170^n.s.^ |
| Cationic antimicrobial peptide CAMP resistance (CAPCR) | Station | r = 0.253 | 2.047 | two-sided | 0.135^n.s.^ |
|  | Temp | r = 0.113 | 1.305 | two-sided | 0.285^n.s.^ |
|  | Depth | r = 0.299 | 2.614 | two-sided | 0.054^n.s.^ |
|  | Salinity | r = 0.388 | 3.046 | two-sided | 0.006** |
|  | DO | r = -0.143 | -1.649 | two-sided | 0.152^n.s.^ |
| Nicotinate and nicotinamide metabolism (NNM) | Station | r = 0.378 | 2.964 | two-sided | 0.045* |
|  | Temp | r = 0.034 | 0.372 | two-sided | 0.808^n.s.^ |
|  | Depth | r = 0.146 | 1.259 | two-sided | 0.277^n.s.^ |
|  | Salinity | r = 0.399 | 3.112 | two-sided | 0.006** |
|  | DO | r = -0.229 | -3.278 | two-sided | 0.054^n.s.^ |
| Folate biosynthesis (FB) | Station | r = 0.377 | 3.132 | two-sided | 0.045* |
|  | Temp | r = 0.044 | 0.471 | two-sided | 0.752^n.s.^ |
|  | Depth | r = 0.177 | 1.610 | two-sided | 0.147^n.s.^ |
|  | Salinity | r = 0.409 | 4.304 | two-sided | 0.006** |
|  | DO | r = -0.223 | -3.374 | two-sided | 0.054^n.s.^ |
| Aminoacyl tRNA biosynthesis (ATB) | Station | r = 0.425 | 3.366 | two-sided | 0.013* |
|  | Temp | r = -0.019 | -0.172 | two-sided | 0.903^n.s.^ |
|  | Depth | r = 0.037 | 0.316 | two-sided | 0.822^n.s.^ |
|  | Salinity | r = 0.375 | 2.959 | two-sided | 0.007** |
|  | DO | r = -0.262 | -2.554 | two-sided | 0.034* |
| Quorum sensing (QS) | Station | r = 0.313 | 2.525 | two-sided | 0.068^n.s.^ |
|  | Temp | r = 0.08 | 0.908 | two-sided | 0.494^n.s.^ |
|  | Depth | r = 0.237 | 2.086 | two-sided | 0.079^n.s.^ |
|  | Salinity | r = 0.399 | 4.480 | two-sided | 0.006** |
|  | DO | r = -0.182 | -2.042 | two-sided | 0.072^n.s.^ |
| mRNA surveillance pathway (MSP) | Station | r = 0.312 | 2.611 | two-sided | 0.064^n.s.^ |
|  | Temp | r = 0.07 | 0.810 | two-sided | 0.542^n.s.^ |
|  | Depth | r = 0.209 | 1.902 | two-sided | 0.094^n.s.^ |
|  | Salinity | r = 0.376 | 3.073 | two-sided | 0.007** |
|  | DO | r = -0.19 | -2.918 | two-sided | 0.063^n.s.^ |
| Base excision repair (BER) | Station | r = 0.355 | 2.870 | two-sided | 0.054^n.s.^ |
|  | Temp | r = 0.056 | 0.611 | two-sided | 0.666^n.s.^ |
|  | Depth | r = 0.199 | 1.757 | two-sided | 0.115^n.s.^ |
|  | Salinity | r = 0.399 | 3.172 | two-sided | 0.006** |
|  | DO | r = -0.199 | -2.958 | two-sided | 0.063^n.s.^ |
| Citrate cycle TCA cycle (CCTC) | Station | r = 0.347 | 2.818 | two-sided | 0.054^n.s.^ |
|  | Temp | r = 0.052 | 0.578 | two-sided | 0.684^n.s.^ |
|  | Depth | r = 0.177 | 1.574 | two-sided | 0.154^n.s.^ |
|  | Salinity | r = 0.392 | 4.284 | two-sided | 0.006** |
|  | DO | r = -0.212 | -3.142 | two-sided | 0.058^n.s.^ |
| Fatty acid biosynthesis (FAB) | Station | r = 0.384 | 2.865 | two-sided | 0.039* |
|  | Temp | r = 0.026 | 0.285 | two-sided | 0.859^n.s.^ |
|  | Depth | r = 0.138 | 1.141 | two-sided | 0.325^n.s.^ |
|  | Salinity | r = 0.401 | 2.991 | two-sided | 0.006** |
|  | DO | r = -0.229 | -3.133 | two-sided | 0.054^n.s.^ |
| Fatty acid degradation (FAD) | Station | r = 0.089 | 0.756 | two-sided | 0.404^n.s.^ |
|  | Temp | r = 0.155 | 1.827 | two-sided | 0.119^n.s.^ |
|  | Depth | r = 0.369 | 2.990 | two-sided | 0.012* |
|  | Salinity | r = 0.279 | 2.087 | two-sided | 0.068^n.s.^ |
|  | DO | r = -0.023 | -0.260 | two-sided | 0.859^n.s.^ |
| Amino sugar and nucleotide sugar metabolism (ASNSM) | Station | r = 0.397 | 3.207 | two-sided | 0.018* |
|  | Temp | r = 0.018 | 0.196 | two-sided | 0.901^n.s.^ |
|  | Depth | r = 0.114 | 1.015 | two-sided | 0.385^n.s.^ |
|  | Salinity | r = 0.398 | 4.073 | two-sided | 0.006** |
|  | DO | r = -0.242 | -3.568 | two-sided | 0.045* |
| Lipopolysaccharide biosynthesis (LB) | Station | r = 0.259 | 1.957 | two-sided | 0.151^n.s.^ |
|  | Temp | r = 0.094 | 1.133 | two-sided | 0.371^n.s.^ |
|  | Depth | r = 0.261 | 2.149 | two-sided | 0.076^n.s.^ |
|  | Salinity | r = 0.359 | 2.672 | two-sided | 0.016* |
|  | DO | r = -0.149 | -1.789 | two-sided | 0.119^n.s.^ |
| Peptidoglycan biosynthesis (PB) | Station | r = 0.384 | 3.118 | two-sided | 0.040* |
|  | Temp | r = 0.014 | 0.152 | two-sided | 0.912^n.s.^ |
|  | Depth | r = 0.086 | 0.766 | two-sided | 0.525^n.s.^ |
|  | Salinity | r = 0.375 | 3.008 | two-sided | 0.007** |
|  | DO | r = -0.243 | -3.586 | two-sided | 0.045* |
| Terpenoid backbone biosynthesis (TBB) | Station | r = 0.396 | 3.097 | two-sided | 0.022* |
|  | Temp | r = 0.000 | 0.012 | two-sided | 0.989^n.s.^ |
|  | Depth | r = 0.069 | 0.580 | two-sided | 0.666^n.s.^ |
|  | Salinity | r = 0.368 | 2.859 | two-sided | 0.009** |
|  | DO | r = -0.243 | -3.479 | two-sided | 0.045* |
| ABC transporters (AT) | Station | r = 0.338 | 2.617 | two-sided | 0.061^n.s.^ |
|  | Temp | r = 0.029 | 0.324 | two-sided | 0.835^n.s.^ |
|  | Depth | r = 0.094 | 0.785 | two-sided | 0.525^n.s.^ |
|  | Salinity | r = 0.360 | 2.765 | two-sided | 0.012* |
|  | DO | r = -0.212 | -3.009 | two-sided | 0.063^n.s.^ |

# Supplementary figures: 5

**Fig. S1. Environmental variability and water column stratification in the Urabá Estuary.** (A) temperature, (B) salinity, and (C) Brunt-Väisälä frequency. PSU = Practical Salinity Unit.

**Fig. S2. Abundance profiling for the ten most abundant taxonomic groups along the Urabá Estuary.** The code format for all samples on x-axis provides information in the following order: station (St) number, sampling depth (at 0.5 or 11 m), and number of replicate (R1 to R3).


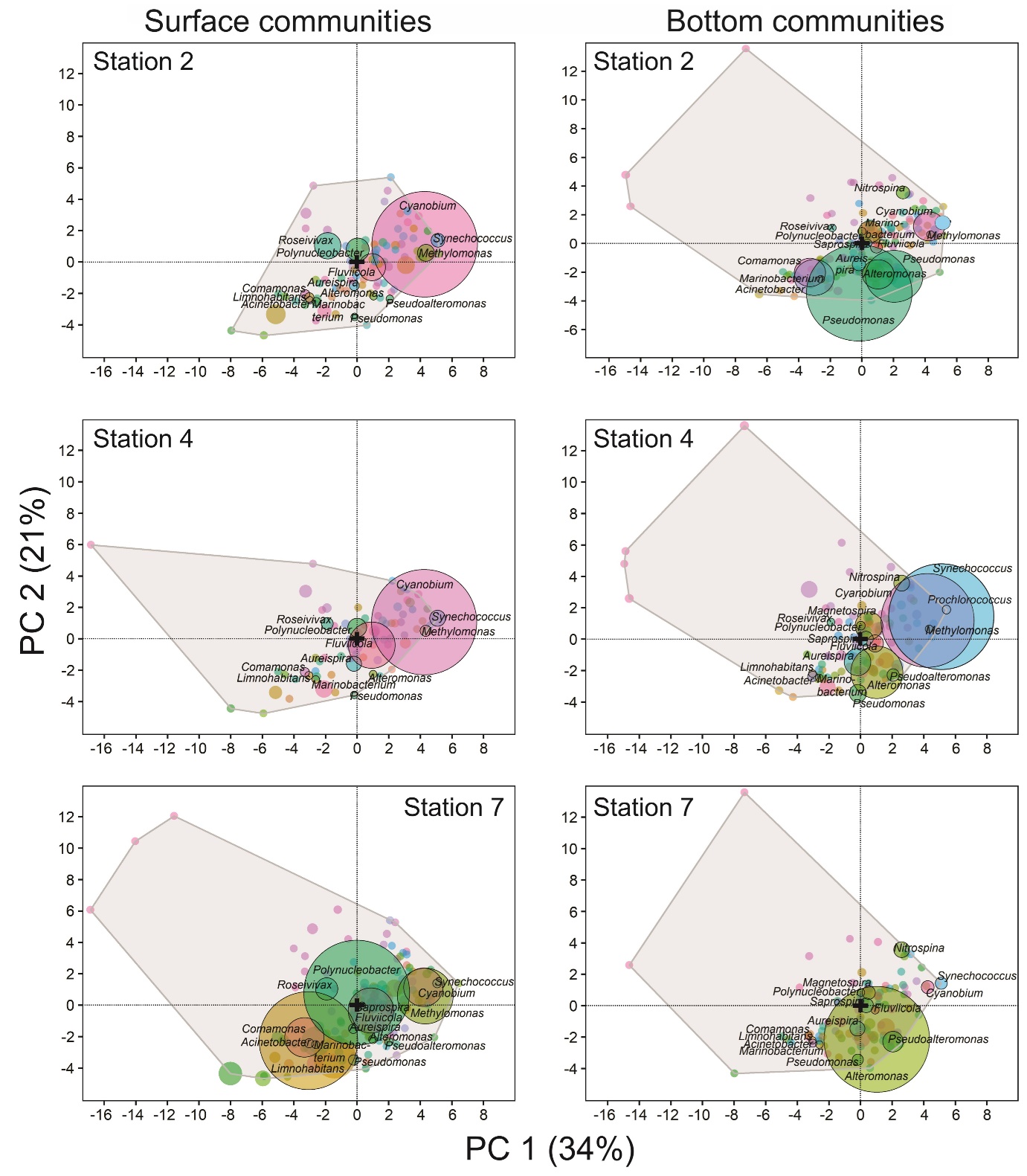


**Fig. S3. Spatial variation of the functional space of prokaryote communities along the Urabá Estuary between St2-St7.** The axes of each panel represent Principal Components 1 and 2 of the PCA space and which explain > 50% of the total variability. Each genus is plotted with circles of different colors considering their specific trait values, while the circle sizes have a proportional relationship with the relative abundance of each genus. Only genera that mostly contributed to cluster differentiation along the transect and among depths are pointed out. The grey area represents functional richness (i.e., the convex hull volumes), and the black cross represents the center of gravity of the enclosed area.


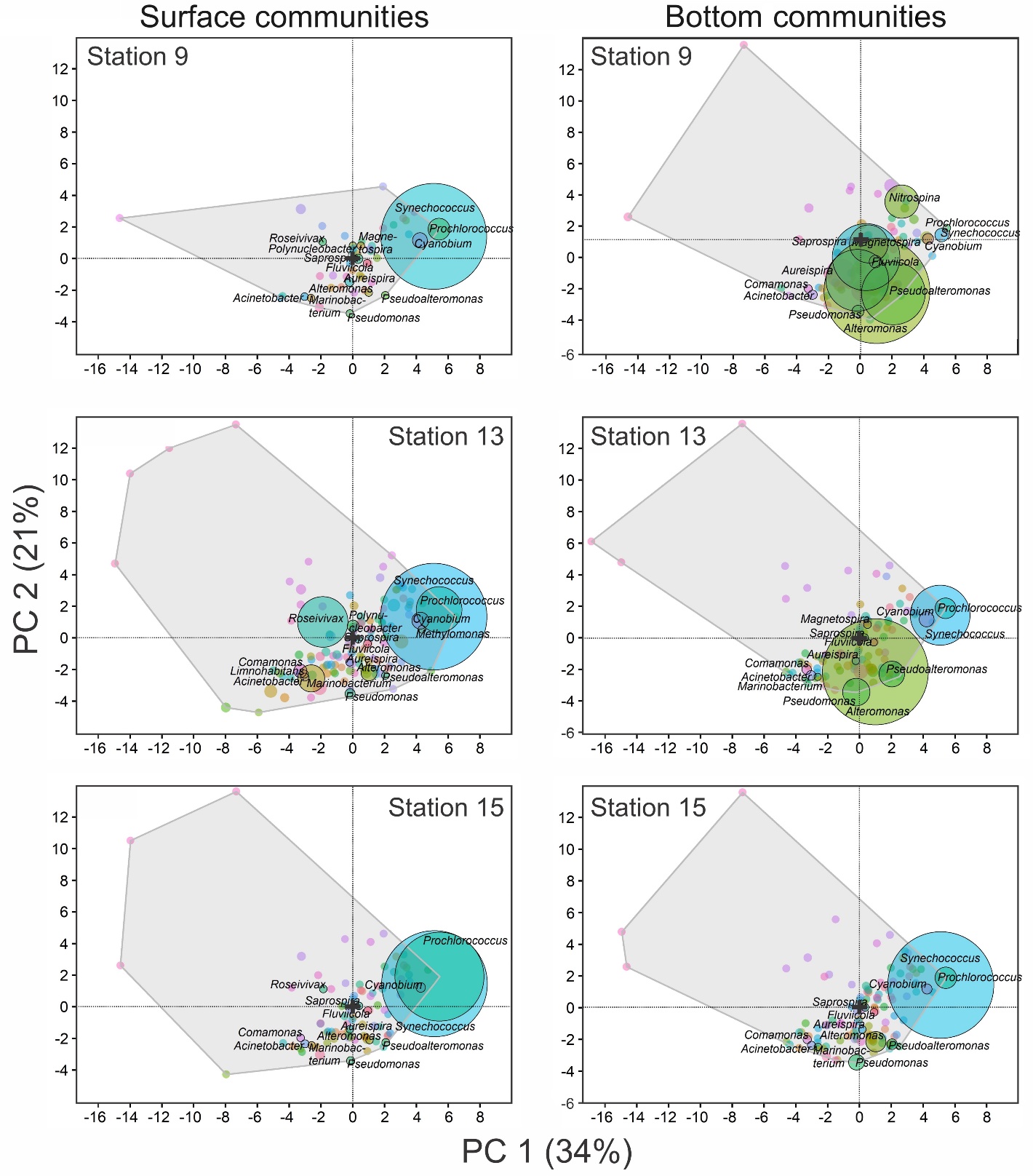


**Fig. S4. Spatial variation of the functional space of prokaryote communities along the Urabá Estuary between St9-St15.** The axes of each panel represent the Principal Components 1 and 2 of the PCA space, which explains > 50% of the total variability. Each genus is plotted with circles of different colors considering their specific trait values, while the circle sizes have a proportional relationship with the relative abundance of each genus. Only genera that mostly contributed to cluster differentiation along the transect and among depths are indicated. The grey area represents functional richness (i.e., the convex hull volumes), and the black cross represents the center of gravity of the enclosed area.


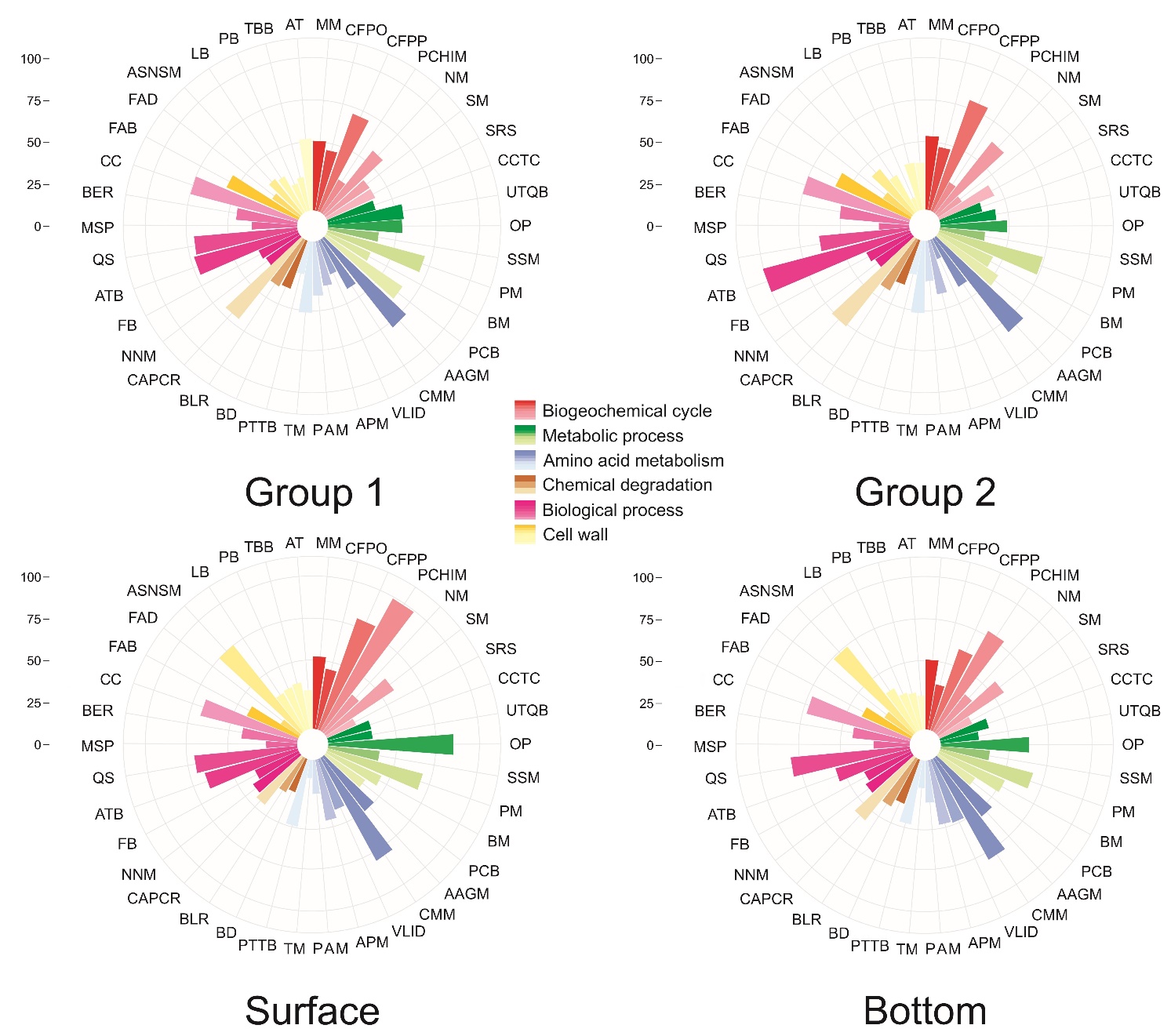


**Fig. S5. Changes in community weighted means (CWMs) of metabolic traits studied in the Urabá Estuary.** Each color scale represents a specific functional category. Traits that fall into one of the six functional categories are represented by acronyms defined in Table 4. Group 1 (St2 to St7) and Group 2 (St9 to St15) represent the homogeneous groups identified by PERMANOVA analyses based on CWMs along the transect and among depths.
